# Highly Conserved Regimes of Neighbor-Base-Dependent Mutation Generated the Background Primary-Structural Heterogeneities along Vertebrate Chromosomes

**DOI:** 10.1371/journal.pone.0002145

**Published:** 2008-05-14

**Authors:** Marcos A. Antezana, I. King Jordan

**Affiliations:** 1 Department of Ecology and Evolution, University of Chicago, Chicago, Illinois, United States of America; 2 Georgia Institute of Technology, Atlanta, Georgia, United States of America; University of Chicago, United States of America

## Abstract

The content of guanine+cytosine varies markedly along the chromosomes of homeotherms and great effort has been devoted to studying this heterogeneity and its biological implications. Already before the DNA-sequencing era, however, it was established that the dinucleotides in the DNA of mammals in particular, and of most organisms in general, show striking over- and under-representations that cannot be explained by the base composition. Here we show that in the coding regions of vertebrates both GC content and codon occurrences are strongly correlated with such “motif preferences” even though we quantify the latter using an index that is not affected by the base composition, codon usage, and protein-sequence encoding. These correlations are likely to be the result of the long-term shaping of the primary structure of genic and non-genic DNA by a regime of mutation of which central features have been maintained by natural selection. We find indeed that these preferences are conserved in vertebrates even more rigidly than codon occurrences and we show that the occurrence-preference correlations are stronger in intronic and non-genic DNA, with the R^2^s reaching 99% when GC content is ∼0.5. The mutation regime appears to be characterized by rates that depend markedly on the bases present at the site preceding and at that following each mutating site, because when we estimate such rates of neighbor-base-dependent mutation (NBDM) from substitutions retrieved from alignments of coding, intronic, and non-genic mammalian DNA sorted and grouped by GC content, they suffice to simulate DNA sequences in which motif occurrences and preferences as well as the correlations of motif preferences with GC content and with motif occurrences, are very similar to the mammalian ones. The best fit, however, is obtained with NBDM regimes lacking strand effects, which indicates that over the long term NBDM switches strands in the germline as one would expect for effects due to loosely contained background transcription. Finally, we show that human coding regions are less mutable under the estimated NBDM regimes than under matched context-independent mutation and that this entails marked differences between the spectra of amino-acid mutations that either mutation regime should generate. In the Discussion we examine the mechanisms likely to underlie NBDM heterogeneity along chromosomes and propose that it reflects how the diversity and activity of lesion-bypass polymerases (LBPs) track the landscapes of scheduled and non-scheduled genome repair, replication, and transcription during the cell cycle. We conclude that the primary structure of vertebrate genic DNA at and below the trinucleotide level has been governed over the long term by highly conserved regimes of NBDM which should be under direct natural selection because they alter drastically missense-mutation rates and hence the somatic and the germline mutational loads. Therefore, the non-coding DNA of vertebrates may have been shaped by NBDM only epiphenomenally, with non-genic DNA being affected mainly when found in the proximity of genes.

## Introduction

In mammals and birds, the amino-acid composition of proteins and the relative occurrence of synonymous codons in coding regions covary strongly with the GC content of the chromosomal regions in which genes are embedded (see reviews by Bernardi [1], [2]. However, upon closer examination GC content loses much of its explanatory appeal. Already Jukes and King [3], e.g., wondered why the occurrence of the amino acid Arginine in mammalian genes departs drastically from that expected given the GC content of genes; and more recently, Antezana and Kreitman [4] pointed out that explanations invoking GC content or G-vs.-C content cannot account for the fact that from *E.coli* to yeast, *Drosophila*, and mammals, the most frequent synonymous codon in each 4fold-degenerate codon family is often the same despite its switching across families from G-ending to C-ending. Therefore explanations invoking GC content effects in particular, and base-composition effects in general, cannot account for important primary-structural features of mammalian and avian coding regions.

Explanations that do not focus on GC content or base composition have been proposed in the past. Most notably, Ruth Nussinov's seminal papers –later often ignored or cited perfunctorily– presented clear evidence that reading-frame-independent preferences for nucleotide motifs may explain the occurrence of several codons and the SC usage in the human and viral genes available then [5], [6], [7]. Nussinov's work followed the discovery in the early 60 s that dinucleotide occurrences are often remarkably non-random in most genomes (tabulated by Setlov [8]. Nussinov introduced the use of randomizations of the placement of SCs along coding regions to estimate the statistical over-/under-representation across successive codons of di- and tri-nucleotide motifs, a randomization that does not allow the base composition, the amino acid content and sequence, and the SC usage to affect the estimates.

Antezana and Kreitman [4], unfortunately unaware of Nussinov's work, developed independently the same randomization and used it to show that in *D.melanogaster* the motifs of the most common SCs are over-represented across codons relative to those of the rarest SCs. To the best of our knowledge, all related work between Nussinov's and Antezana and Kreitman's relied on motif preferences that could be influenced by base and codon composition as well as by the amino-acid composition and sequence of encoded proteins, despite the otherwise remarkable applied-mathematical sophistication deployed at times. Karlin and Mrazek [9], e.g., “predicted” whole-genome codon occurrences in humans on the basis of the *within-codon* occurrence of 12_, _23, and 1_3 dinucleotides and of the base composition at each codon position, quantities that obviously can be affected strongly by protein encoding and mRNA translatability (123 being a codon with its three positions labelled by the numbers 1, 2, and 3).

Here we expand on Antezana and Kreitman's explanation for the phylogenetically conserved alternation of G- and C-ending codons as most-frequent SC in 4fold-degenerate codon families, and show that in vertebrates in general across-codon trinucleotide preferences correlate positively, linearly, and strongly with the whole-genome occurrence of SCs, of non-stop codons in general and, less markedly, with that of encoded amino acids, and we also show that these preferences are very similar across vertebrate genomes, to the point of being almost identical in mammals and *Gallus*. Furthermore, we show that the GC content of non-genic, intronic, and coding DNA correlates positively, linearly, and with astounding 94%+ R^2^s with the balance between the motif preferences for TA-rich motifs and those for GC-rich ones. This correlation –which was observed in the coding DNA of every warm- and cold-blooded vertebrate we could examine– indicates that the forces behind motif preferences vary drastically along chromosomes, generating GC content heterogeneity as a side effect (for neither motif preferences nor this motif-preference-derived GCvsAT “balance” can be influenced by base composition).

We then show that the trinucleotide preferences, the preference-occurrence correlations, and the trinucleotide occurrences themselves, as well as the correlation between GC content and the GCvsAT balance must be to a great extent the product of mutation rates that depend strongly on the bases present at the site preceding and the site following each mutating site. Indeed i) we find very similar motif preferences in non-genic and intronic DNA, which indicates strongly that motif preferences are the product of mutation pressure and genetic drift shaping coding and non-coding DNA alike; and ii) we recreate to a great extent the native occurrences and preferences, the native occurrence-preference correlations, and the native correlations between GC content and the GCvsAT balance, by bringing DNA sequences to equilibrium under regimes of neighbor-base-dependent mutation (NBDM) that we estimate from substitutions retrieved from alignments of non-genic, intronic, and coding mammalian DNA that were sorted and grouped by GC content.

Finally, and perhaps most importantly in terms of ultimate molecular-evolutionary causation, we show computationally that these empirically estimated regimes of NBDM generate in mammalian coding regions a spectrum of non-synonymous point mutations that is qualitatively and quantitatively drastically different from that which their matched –but counterfactual– mutation regimes lacking context-dependence would generate. Therefore NBDM may actually be a major determinant of individual fitness in vertebrates because it may reduce markedly the somatic and germline mutational loads entailed by non-synonymous point mutations, where the rigid phylogenetic conservation of motif over-/under-representations in genic DNA across cold- and warm-blooded vertebrates alike indicates that NBDM is also rigidly conserved in vertebrates and that load challenges due to non-synonymous mutations may be very similar.

## Materials and Methods

### Coding-region data

We relied primarily on vertebrate coding-region sequences (CDSs) available from the ENSEMBL website. The CDSs of the sea squirt *Ciona intestinalis* were downloaded from the DOE Joint Genome Institute's website. All results with the human CDSs from ENSEMBL were confirmed i) in a dataset of 13,000 human genes for which transcripts are known (kindly provided by A. Urrutia), and ii) in a dataset of 12,717 human CDSs with known mouse homologues [10]. We also took advantage of a dataset with 2,165 aligned homologous coding regions from human, mouse, and rat [11]. The *Drosophila melanogaster* CDSs were also from ENSEMBL and the *Drosophila pseudoobscura* CDSs were downloaded from the FLYBASE website. In some plots we included results for 90 ribosomal-protein genes which were also retrieved from ENSEMBL

### Codon occurrences

“Whole-genome” codon frequencies were calculated by dividing the number of times each codon appears in all genes in a genome by the total number of codons in all genes in the same genome, excluding stop-codons. Whole-genome SC frequencies were computed by dividing the total number of each synonymous codon by the total number of codons belonging to the same SC family (the three 6fold-degenerate families were analyzed both as single families and by separating each family into a 2fold-degenerate subfamily and a 4fold-degenerate subfamily). Whole-genome amino acid frequencies were computed by dividing the total number of codons for each amino acid by the total number of all codons.

### Base-composition predictions of occurrences

In several instances we compared empirical motif and codon occurrences to predictions derived from base composition. We mainly focussed on predictions that assume a single base composition that we estimated numerically by checking every possible base composition (with granularity 0.001) as to its ability to minimize the sum of the absolute values of the differences between predicted and observed occurrences. We did not use occurrence predictions that assume a different base composition at the 1st-, 2nd-, and 3rd-codon position, predictions that albeit attractive in terms of fitting potential are biologically absurd since they require a mechanism that lets DNA sequences mutate differently at the three codon positions.

### Non-genic DNA

We took advantage of 4,677 alignments covering a total of 9.78Mb of human, chimp, and baboon non-coding DNA curated by Julien Meunier [12]. These sequences contain no exons from known gene and therefore are likely to consist mainly of non-genic DNA. For this reason we will take the liberty of referring to them as “non-genic”. Runs of three or more identical trinucleotides were ignored. We also used the whole-genome dinucleotide proportions tabulated in Setlov [8] that had been measured until that time by several workers.

### Intronic DNA

We used a dataset of 53,792 segments of human introns that was curated and kindly provided by Fyodor Kondrashov to IKJ [13]. And we also used a dataset of aligned intron segments from humans, chimp, and macaque covering all chromosomes except for the X and the Y. This dataset was generated by IKJ as follows: *Homo*logous intronic regions from human, chimpanzee, and macaque were identified in the multiple-sequence alignments of sixteen vertebrate genome assemblies to the human genome reference sequence hg18 (NCBI build 36.1) provided by the UCSC Genome Browser (http://genome.ucsc.edu/ [14], 15. The whole-genome multiple-sequence alignments were assembled using the Multiz tool [16]. Intron coordinates were taken from the Known Genes track of the UCSC Table Browser, which stores the most reliable gene-model data from the Uniprot and Refseq databases. As it was the case for non-genic DNA, runs of three or more identical trinucleotides were ignored.

### Estimation of across-codon motif preferences in a single gene

The across-codon over-/under-representation of (“preference for”) each nucleotide motif in a given coding region was quantified by comparing the number of appearances of the motif in the two non-coding frames of the gene (i.e., its “count”, e.g., GC|A+G|CA, where “|” is the codon boundary) to the distribution of the same count in 1000 sequences of the gene in which the gene's codons were placed randomly and without replacement at any location in the original sequence at which the amino acid they encode is encoded. The motif preferences obtained from this randomization of the location of confamilial SCs are therefore independent from, and unaffected by, those properties of the coding region that are kept constant during the randomization, i.e., the base, codon, and amino acid compositions, as well as the encoded amino acid sequence. Therefore, the motif preferences we measure cannot be generated by mononucleotide-level effects like GC content, etc., although they can produce patterns that can be misinterpreted as having been caused by mononucleotide-level forces. Note, however, that the over-/under-representations can be made less marked by mutation processes lacking neighbor-base-dependence (we thank R.R. Hudson for pointing this out).

The ***motif-preference index*** that we use is an empirical *Z* value, i.e., the signed difference between the observed count and the average count; the whole divided by the standard deviation of the count, where both the average and the standard deviation are estimated over the same 1000 randomized sequences mentioned above. This index has an expected value of 0.00 when averaged over many coding regions with randomized SC positioning, i.e., every motif has an expected across-codon preference of 0.00 when confamilial synonymous codons appear at random locations along an analyzed sequence (see below). In a previous paper [4], MAA used the *p*-value of the observed motif's count as the motif's over-/under-representation score, but meanwhile it became clear to us that this index loses resolving power when *p*-values become very large or very small (i.e., close to 1.0 or 0.0). As indices we also tried a *Chi* value as well as the *Z* value that one can obtain from the p-value of each motif if one assumes that the p-value comes from a Z random variable that is N(0,1) normally distributed; but these alternative indices delivered slightly less satisfactory results.

### Estimation of motif preferences in non-coding DNA

Motif preferences in non-coding DNA were also expressed as Z values but were estimated by shuffling sites randomly along each sequence rather than by randomizing SC locations. This generates for each native non-coding sequence a set of joint empirical null distributions that controls for effects due to discreteness and for the positive and negative correlations between the over-/under-representations of many motifs in each individual sequence, effects that can mar simultaneous “analytical” estimations of multiple motif preferences in a single sequence.

### Estimation of average motif preferences over many sequences

From each genome and for each di- and tri-nucleotide motif we calculated a “whole-genome” index of across-codon preference. The “whole-genome” preference for a motif is simply the average of the preference for that motif in each gene, over all genes in a dataset. We confirmed that the null expectation of the whole-genome across-codon preference for each motif is 0.00 by randomizing the placement of SCs in each gene in the human dataset before estimating each gene's motif preferences and then using these randomized preferences to estimate a new set of average preferences. Such average preferences and null expectations were also estimated for motif preferences derived from native and site-randomized ***intronic and non-genic DNA***. In such a *non-genic sequence* (and in a large concatenated one) motif preferences as we measure them are not independent from motifs occurrences –unlike the across-codon motif preferences and codon occurrences of a single coding region– but when the preferences from multiple sequences are averaged they too have an expectation 0.00 under the null hypothesis.

### Correlations between codon occurrences and motif preferences

To evaluate heuristically the congruence of codon occurrences and across-codon motif preferences for whole genomes or large groups of genes, we plotted codon occurrences against the preferences for the corresponding trinucleotides averaged over the genes in each gene group or genome. Therefore in the plots below, *the genomic “preference” for a given codon* will be the whole-genome across-codon preference for the corresponding trinucleotide, and *the genomic “preference” for a particular amino acid* will be the sum of the whole-genome preferences for all the trinucleotide motifs of the codons encoding the amino acid of interest, divided by the sum of the whole-genome preferences for the motifs of the 61 sense (non-stop) codons. We also examined this relationship for subgroups of trinucleotide motifs defined by the degeneracy of amino acid encoding, i.e., for 2fold-, 4fold-, and 6fold-degenerate codons. These heuristic subgroups delivered richer patterning than those defined by the columns or rows of the genetic code table, but it is likely that more instructive ways to define subgroups will become apparent in the future. Additionally we also looked at each SC family separately, to minimize occurrence effects due to amino-acid selection. Finally, most of the above was also done using groups of sequences of similar GC content: We first sort the sequences in a dataset according to increasing GC content (e.g., at third codon positions) and then create equal-sized, non-overlapping groups of sequences of similar GC (across genomes such groups never contained fewer than 1000 sequences each). Then for each such subgroup we calculate the average GC content of interest, a set of average motif preferences, etc. Most of the above was also done for the ***intronic and non-genic datasets***.

### Correlations of GC content with “GCvsAT pressures” derived from motif preferences

We correlated the average 1st-, 2nd-, and 3rd-position GC content, as well as the 1st+2nd+3rd-position GC content of the aforementioned vertebrate coding regions, to heuristic GC-content “pressures” derived from trinucleotide- or dinucleotide-motif preferences. To this end, for each of the aforementioned groups of GC-sorted sequences, we calculate an average pressure towards the GC content(s) of interest. For instance, the pressure for 3rd-position GC is simply the sum of a given group's average preferences for the trinucleotide motifs ending in CorG, minus that of the preferences for the trinucleotides ending in TorA. Along the same lines, the 1st-position GCvsAT pressure is the sum of the preferences for the trinucleotides starting with CorG minus the sum for the trinucleotides starting with TorA; the 2nd-position GCvsAT pressure is the sum of the preferences for trinucleotides with CorG in their second base minus that of the preferences for trinucleotides with TorA in their second base; and the 1st+2nd+3rd-position “total” GCvsAT pressure is the sum of every trinucleotide-motif preference after multiplying the preferences by 3.0 when the trinucleotide of concern has three CorGs, by 1.0 if it has two CorGs and one TorA, by −1.0 if it has one CorG and two TorAs, or by −3.0 if it has three TorAs. Trinucleotide-level pressures were divided by 96, the maximal value the GC123 GCvsAT pressure can assume. The dinucleotide-level GCvsAT pressure was simply the sum of each average dinucleotide preference multiplied by 2.0 if the dinucleotide has two CorGs, by −2.0 if it has two TorAs, and by 0.0 otherwise. Dinucleotide-level GCvsAT pressures were divided by 16.

We will show below that these GCvsAT pressures correlate with astounding 95%+ R^2^s with the actual GC content of mammalian, avian, and teleostean coding regions (and up to 99.9% in simulated data) but not at all when SC positions are randomized before motif-preference estimation. However, we are desolate to confess that we do not know the mathematical reasons for the felicitous behavior of these heuristic GCvsAT pressures. We did, however, try to dissect non-parametrically the contribution of specific motifs or pairs thereof to these correlations by performing systematic one- and two-motif jack-knifings of the R^2^ values, but we could not find any motif or motif pairs whose exclusion sank the correlations strongly (see Results). For ***intronic and non-genic DNA*** only total GCvsAT pressures were estimated.

### Simulations to generate motif over- and under-representations

We wrote a program to simulate the action of neighbor-base-dependent mutation (NBDM) in DNA sequences. In the program, the probability of a base mutating towards one of the other bases is dependent not only on the departure base and the arrival base, but also on the bases at the site preceding and at that following the site of interest. The user provides therefore a matrix with 64×4 rates (the “no change” rate is ignored; i.e., tTt to tTt tCt tAt tGt; tTc to tTc tCc tAc tGc;… gGg to gTg gCg gAg gGg). We used this program to explore the relationship between across-codon motif preferences and actual di- and tri-nucleotide occurrences after NBDM has hit multiple times every site in a sequence. To avoid concatenation of adjacent mutation events the program first chooses a site randomly along the whole sequence and then mutates it according to the three applicable rates in the 64×4 matrix (we thank R.R. Hudson for this additional precaution). The whole is repeated until each site is hit a user-defined number of times. For the results shown below, we hit each site at least 10 times.

Additionally, to study how ***amino-acid selection*** may blur the relationship between motif preferences and codon occurrences, we added to the simulation a mutation sieve that accepts or rejects each generated mutation according to a user-provided matrix with 64×64 codon-change acceptabilities. One such 64×64 acceptability matrix was derived from the Grantham matrix [17] as follows: The acceptability of a replacement from amino acid *i* to amino acid *j* was equal to 1.0−Grantham(*i*,*j*)/[1.1*max(Grantham)], where Grantham(*i*,*j*) and max(Grantham) are the Grantham distance between amino acids *i* and *j* and the largest distance in the Grantham matrix, respectively. The conversion gives a maximum acceptability of 1.0−min(Grantham)/[1.1*Max(Grantham)], where min(Grantham) and max(Grantham) are the smallest and the largest distances in the Grantham matrix; and the multiplication by 1.1 avoids giving 0.0 acceptability to replacements involving the two amino acids separated by the largest Grantham distance. Synonymous changes were given acceptability 1.0. The matrix was generated and kindly provided by Hua Tang. We obtained comparable results with acceptability matrices derived from the EX and the Blosum100 distance matrices [18], [19]. The EX and the Blosum100 acceptability matrices were kindly provided by Arlin Stoltzfuss and are described in Yampolsky and Stoltzfuss [18].

### Estimation of neighbor-base-dependent substitution rates

We estimated 64×4 substitution/mutation matrices from the substitutions inferrable from the aforementioned alignments of non-genic, intronic, and coding DNA, with the baboon non-genic sequences, the macaque intronic sequences, and the human CDSs being used as outgroup, respectively. A substitution or mutation traced to a branch leading to a non-outgroup sequence was scored only if the outgroup base was found in the other non-outgroup sequence and if, at the same time, the outgroup bases at the two surrounding sites were also found in the non-outgroup sequence displaying the derived base. This excluded contiguous changes in the same lineage. We will use these NBDM rates to explore the relationship between motif preferences and occurrences and to look for large differences between the patterns generated by them and by the matched context-independent mutation regimes, and therefore it appears safe to disregard the subtle biases that may arise from ignoring adjacent substitutions. Such 64×4 matrices were also estimated for subsets of the alignments sorted according to the GC content of the outgroup sequence. This sorting by GC content by no means guarantees a pooling of substitutions that respects the whole gamut of similarities and differences that may relate and distinguish the various regimes of NBDM active within a genome. It would be indeed very surprising if GC content turned out to be the best summary statistic for sorting, if there is one. For this reason each estimated GC-specific matrix must be “blunted” to some extent by the fact that its estimation relied on pooled substitutions that were generated by heterogeneous mutation regimes. The number of substitutions/mutations used to estimate each matrix will be given when presenting the results.

## Results

### Relationship between codon occurrences and trinucleotide-motif preferences

**Figure 1:**
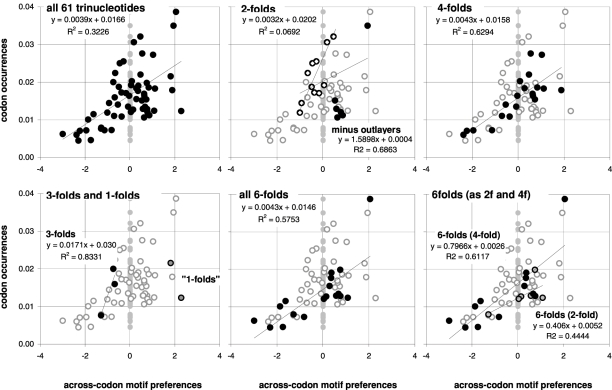
Human codon occurrences and trinucleotide-motif preferences. On the top left are the total occurrences of the 61 non-stop codons in 33,860 human coding regions (CDSs; vertical axis) plotted against the average across-codon over-/under-representation of (“preference for”) the corresponding 61 trinucleotide motifs in the same CDSs (horizontal axis). The average preference for a motif is the average over all CDSs of the Z value for the motif's 23∥1+3∥12 “off-frame” count in each CDS, where the set of 61 Z values of each CDS is estimated using 10,000 randomizations of the locations of its synonymous codons (SCs; see M&Ms). Grey dots are for “null” motifs preferences obtained by randomizing each CDS' SC locations before estimating each CDS' motif preferences and averaging over CDSs. The other plots highlight motif groups defined by the genetic code's degeneracy. In the 2-folds plot (top middle) the “minus outlayers” R^2^ and slope are for the “*3f -3aas*” group created by excluding the codons of three outlayer 2fold families (solid circles; see text).

In Figure 1 we plot the average across-codon preferences for the trinucleotide motifs of the 61 sense (non-stop) codons against the occurrence frequencies of the 61 sense codons in 33,860 human coding regions (CDSs). Identical results were obtained with the CDSs with known mouse homologues or known transcripts (not shown). The occurrence-preference R^2^ value is about 32%. The correlation disappears when the CDSs' synonymous-codon (SC) location is randomized before estimating each CDS's motifs preferences (vertical gray points). In the surrounding plots in the same figure, we highlight the points of the trinucleotides belonging to 2-, 3-, 4-, and 6-fold SC families and the two codons for non-degenerately encoded amino acids, using as faded background the other points. The R^2^ values are about 7, 83, 63, and 58%, respectively (and 44 and 61% for the 2- and 4-fold subfamilies of the 6-folds).

These occurrence-preference R^2^ values are large and point to a dependence of whole-genome codon occurrences from the forces responsible for the over-/under-representations of across-codon trinucleotide motifs (since such motif preferences cannot be influenced by standard in-frame in-frame features; see methods). Note that removing the codons for Gln, His, and Cys, i.e., the six codons for three of the nine 2fold amino acids, increases the 2fold occurrence-preference R^2^ tenfold from to 7 to 69%. These three amino acids are special biochemically, and is tempting to speculate that selection is behind their “below-expectation” occurrence (see simulation results below). We will refer to this subgroup of 2fold motifs/codons as the “2f -3aas” group.

### Evolutionary conservation of the occurrence-preference relationship

In Table 1 we present the corresponding occurrence-preference R^2^s and slopes (left, right) for *Homo*, the opossum, the platypus, *Gallus*, *Xenopus*, *Danio*, *Fugu*, and *Ciona* (the patterns for *Mus*, *Rattus*, and *Canis* are very similar to *Homo*'s unless otherwise noted). In general the trends are similar to those in Figure 1, with opossum, *Gallus*, and *Xenopus* showing the highest “all-motifs” R^2^s and with platypus, *Fugu*, and *Ciona* showing the lowest ones. The slopes are all positive and very similar across taxa if one excepts the 0.000 slope (and 0% R^2^) for *Xenopus*' 2folds. The motif groups with strongest correlations are the 4folds and 6folds. Note that the platypus R^2^s tend to be similar to *Fugu*'s as those of mammals and *Gallus* are quite similar too (which may be convergence).

**Table 1:** R^2^s and slopes of the correlation between codon occurrences and motif preferences.

	R^2^ values								slopes							
	*Homo*	oposs.	platyp.	*Gallus*	*Xenop.*	*Danio*	*Fugu*	*Ciona*	*Homo*	oposs.	platyp.	*Gallus*	*Xenop.*	*Danio*	*Fugu*	*Ciona*
all	32%	40%	23%	36%	39%	29%	24%	26%	0.004	0.004	0.005	0.004	0.004	0.004	0.006	0.007
2folds	7%	9%	14%	2%	0%	2%	11%	5%	0.003	0.003	0.006	0.002	0.000	0.001	0.006	0.004
4folds	63%	85%	23%	86%	88%	69%	22%	60%	0.004	0.004	0.004	0.005	0.005	0.005	0.004	0.008
2f -3aas	69%	51%	50%	66%	46%	63%	52%	15%	0.014	0.009	0.011	0.011	0.011	0.011	0.017	0.007
6folds	58%	81%	41%	71%	88%	62%	46%	32%	0.004	0.004	0.005	0.004	0.005	0.005	0.008	0.006
2f (6fold)	47%	75%	45%	80%	77%	68%	45%	26%	0.003	0.002	0.004	0.003	0.002	0.003	0.007	0.004
4f (6fold)	66%	84%	45%	75%	91%	65%	51%	35%	0.005	0.004	0.006	0.005	0.005	0.005	0.009	0.006

Table 2 lists the R^2^s and slopes for between-genome comparisons of codon occurrences (left) and of motif preferences (right). It shows that the evolutionary conservation of the occurrence-preference correlations is due to the conservation of both codon occurrences and motif preferences across species. Most R^2^s in Table 2 are indeed very high except for the occurrence-occurrence comparisons involving *Ciona*. However, the occurrence-occurrence R^2^s for vertebrates vs. *Ciona* are much lower than those, e.g., for vertebrates vs. *Drosophila* (and the preference-preference R^2^s for vertebrates vs. *Drosophila* are above 45%; neither is shown) so that *Ciona* appears to be exceptional. Note also that the 97%+ preference-preference R^2^s for *Gallus* vs. *Homo* are clearly higher that the corresponding occurrence-occurrence R^2^s (a result that also holds for *Gallus vs. Mus*, *Rattus*, or *Canis*; not shown). Among motif subgroups the occurrences and preferences of 6folds appear to be the most conserved.

**Table 2:** R^2^s and slopes of the correlations between codon occurrences or motif preferences.

		whole-genome codon frequencies								preferences for across-codon trinucleotides							
		*Homo*	*oposs.*	*platyp.*	*Gallus*	*Xenop.*	*Danio*	*Fugu*	*Ciona*	*Homo*	*oposs.*	*platyp.*	*Gallus*	*Xenop.*	*Danio*	*Fugu*	*Ciona*
all 61 codons	*Homo*		0.834	0.954	0.899	0.667	0.909	1.008	0.270		1.077	0.660	0.976	0.836	0.864	0.558	0.288
	*oposs.*	83%		0.891	0.995	0.842	0.977	0.888	0.574	97%		0.605	0.877	0.759	0.779	0.504	0.269
	*platyp.*	92%	68%		0.823	0.578	0.844	1.043	0.173	91%	91%		1.340	1.145	1.213	0.806	0.402
	*Gallus*	89%	91%	73%		0.798	0.990	0.950	0.513	97%	93%	88%		0.840	0.885	0.562	0.314
	*Xenop.*	67%	91%	50%	88%		1.068	0.867	0.815	97%	95%	87%	96%		1.014	0.646	0.376
	*Danio*	87%	84%	74%	94%	79%		0.988	0.450	86%	83%	82%	89%	86%		0.630	0.327
	*Fugu*	85%	55%	90%	69%	41%	78%		0.095	87%	84%	87%	87%	84%	96%		0.481
	*Ciona*	9%	35%	4%	30%	55%	24%	1%		37%	39%	35%	43%	46%	41%	37%	
2folds	*Homo*		0.797	0.927	0.903	0.584	0.939	0.973	0.188		0.947	0.673	1.049	0.809	1.110	0.689	0.293
	*oposs.*	83%		0.924	1.019	0.768	0.978	0.802	0.510	91%		0.679	0.996	0.758	1.056	0.665	0.292
	*platyp.*	96%	73%		0.925	0.571	0.973	1.084	0.170	81%	81%		1.242	0.921	1.365	0.886	0.408
	*Gallus*	94%	91%	88%		0.701	0.991	0.922	0.385	98%	86%	77%		0.769	1.016	0.626	0.310
	*Xenop.*	68%	90%	59%	85%		1.145	0.817	0.835	94%	81%	68%	95%		1.261	0.783	0.396
	*Danio*	93%	77%	90%	90%	69%		1.004	0.254	89%	79%	75%	84%	80%		0.629	0.210
	*Fugu*	77%	40%	86%	60%	27%	78%		−0.074	84%	76%	77%	77%	75%	97%		0.308
	*Ciona*	5%	27%	3%	17%	47%	8%	1%		22%	21%	24%	28%	28%	16%	14%	
4folds	*Homo*		0.806	0.920	0.708	0.575	0.683	0.846	0.032		1.058	0.632	0.931	0.825	0.764	0.503	0.262
	*oposs.*	77%		0.725	0.863	0.799	0.838	0.736	0.345	98%		0.597	0.853	0.770	0.708	0.470	0.251
	*platyp.*	79%	42%		0.441	0.310	0.447	0.825	−0.229	94%	95%		1.355	1.226	1.133	0.775	0.404
	*Gallus*	66%	83%	27%		0.865	0.954	0.747	0.525	95%	91%	86%		0.858	0.837	0.529	0.306
	*Xenop.*	52%	84%	16%	89%		0.967	0.629	0.711	96%	96%	91%	95%		0.929	0.593	0.361
	*Danio*	61%	78%	28%	90%	78%		0.803	0.525	80%	78%	75%	88%	83%		0.629	0.394
	*Fugu*	83%	53%	85%	49%	29%	58%		−0.039	81%	81%	83%	82%	80%	93%		0.558
	*Ciona*	0%	14%	8%	29%	44%	29%	0%		33%	34%	33%	41%	44%	54%	46%	
2f -3aas	*Homo*		0.807	0.901	0.844	0.508	0.897	0.942	0.155		1.088	0.965	1.074	0.698	1.011	0.637	0.540
	*oposs.*	78%		0.812	0.923	0.710	0.827	0.597	0.555	86%		0.749	0.819	0.495	0.781	0.489	0.376
	*platyp.*	94%	64%		0.853	0.461	0.939	1.115	0.096	90%	75%		1.003	0.658	1.027	0.669	0.535
	*Gallus*	91%	91%	80%		0.688	0.969	0.849	0.446	98%	78%	87%		0.653	0.923	0.580	0.556
	*Xenop.*	53%	88%	38%	77%		0.959	0.454	1.008	89%	62%	81%	92%		1.277	0.815	0.840
	*Danio*	89%	64%	85%	82%	49%		1.036	0.186	77%	64%	82%	76%	67%		0.672	0.392
	*Fugu*	66%	22%	80%	42%	7%	72%		−0.186	65%	53%	74%	64%	58%	96%		0.533
	*Ciona*	3%	30%	1%	18%	57%	4%	5%		36%	24%	36%	45%	47%	25%	22%	
6folds	*Homo*		1.965	2.426	1.857	2.104	1.746	0.052	0.525		1.011	0.985	1.028	0.947	0.659	0.293	0.182
	*oposs.*	82%		1.239	0.949	1.075	0.870	0.045	0.248	98%		0.971	1.012	0.935	0.649	0.283	0.171
	*platyp.*	96%	73%		0.752	0.844	0.690	0.041	0.207	91%	91%		1.040	0.962	0.668	0.298	0.196
	*Gallus*	95%	91%	85%		1.132	0.868	0.095	0.247	98%	97%	91%		0.910	0.632	0.306	0.189
	*Xenop.*	72%	93%	59%	87%		0.755	0.078	0.210	99%	98%	90%	98%		0.685	0.290	0.170
	*Danio*	92%	87%	81%	97%	83%		−0.008	0.309	91%	89%	88%	90%	90%		0.396	0.249
	*Fugu*	95%	68%	96%	86%	58%	86%		−0.082	92%	91%	92%	91%	91%	97%		0.567
	*Ciona*	1%	17%	0%	8%	30%	8%	0%		31%	31%	25%	37%	35%	33%	30%	
2f (6folds)	*Homo*		0.561	0.996	0.835	0.320	0.744	1.283	−0.569		1.138	0.780	1.124	0.770	0.987	0.628	0.413
	*oposs.*	60%		1.048	1.031	0.595	1.148	1.247	−0.155	94%		0.648	0.892	0.644	0.825	0.523	0.355
	*platyp.*	95%	55%		0.804	0.296	0.671	1.295	−0.547	96%	92%		1.368	0.943	1.247	0.777	0.449
	*Gallus*	95%	76%	93%		0.449	0.931	1.421	−0.429	94%	82%	88%		0.666	0.799	0.500	0.426
	*Xenop.*	45%	82%	40%	65%		1.633	1.327	0.676	95%	92%	90%	96%		1.203	0.742	0.665
	*Danio*	75%	93%	64%	86%	82%		1.133	−0.224	97%	94%	98%	86%	90%		0.627	0.356
	*Fugu*	88%	43%	93%	79%	21%	51%		−0.594	95%	91%	92%	81%	83%	95%		0.458
	*Ciona*	21%	1%	21%	9%	7%	2%	44%		34%	34%	25%	48%	54%	25%	17%	
4f (6folds)	*Homo*		0.737	1.008	0.914	0.681	0.889	1.153	0.125		1.090	0.691	0.988	0.830	0.883	0.576	0.184
	*oposs.*	86%		1.134	1.130	0.940	1.089	1.279	0.323	99%		0.633	0.900	0.754	0.799	0.522	0.168
	*platyp.*	96%	76%		0.840	0.599	0.812	1.123	0.056	90%	91%		1.305	1.089	1.184	0.790	0.225
	*Gallus*	96%	93%	86%		0.782	0.979	1.195	0.222	99%	99%	91%		0.834	0.893	0.585	0.193
	*Xenop.*	79%	95%	65%	91%		1.127	1.263	0.393	99%	98%	90%	98%		1.066	0.694	0.227
	*Danio*	93%	88%	83%	99%	88%		1.214	0.224	90%	89%	86%	91%	91%		0.640	0.215
	*Fugu*	96%	74%	96%	89%	67%	90%		0.066	92%	91%	91%	93%	93%	98%		0.326
	*Ciona*	6%	26%	1%	17%	36%	17%	2%		32%	32%	25%	35%	34%	38%	36%	

**Figure 2:**
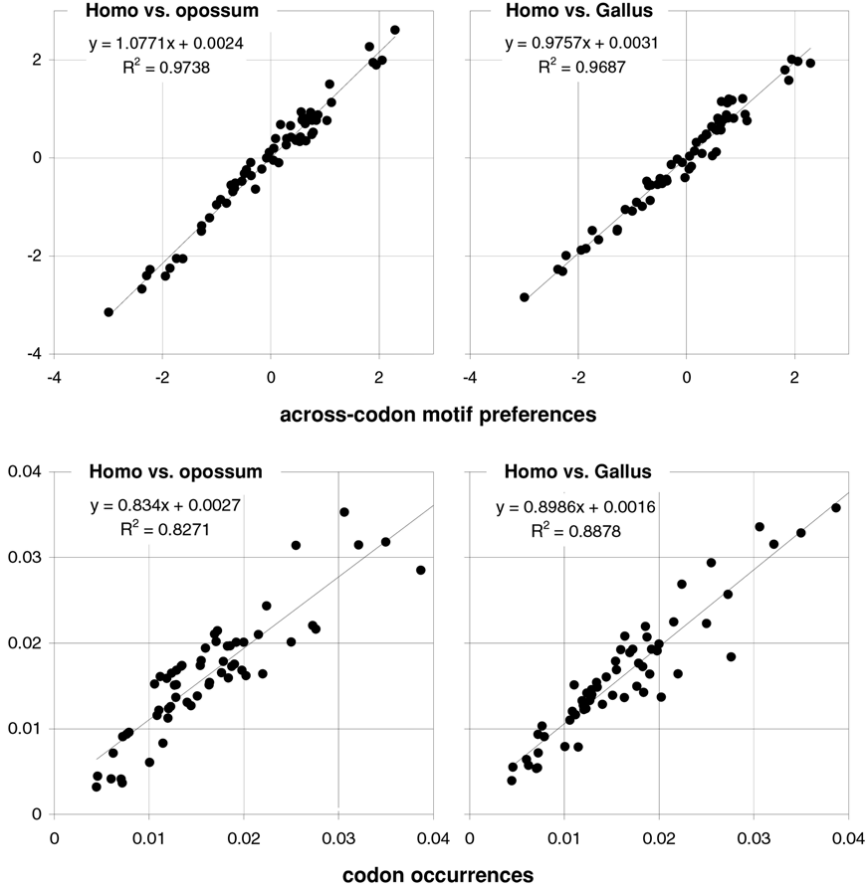
The conservation of codon occurrences and trinucleotide-motif preferences in homeotherm vertebrates. Plots of the preferences for across-codon trinucleotides in *Homo* vs. opossum or *Gallus* (left and right, top) and of whole-genome codon occurrences in *Homo* vs. opossum or *Gallus* (left and right, bottom). *Homo*'s values are on the horizontal axis.

Therefore, motif preferences appear to be more phylogenetically conserved than codon occurrences during evolution, with occurrences being especially divergent in *Ciona* (and in *D.melanogaster*; 0.4% R^2^ vs. *Homo*; not shown). Concededly, motif preferences could be the result of only a few major mutational mechanisms that selection could preserve more easily over evolutionary time than say 64 independent processes or 64 independent codon occurrences, but realizing this does not make the preferences, their structuring consequences for the genome, and the possible functional implications of them and/or of what generates them, any less conserved. In Figure 2, we plot the motif preferences of *Homo* against those of the opossum and *Gallus* (both 97% R^2^) as well as the corresponding plots for codon occurrences (83 and 89% R^2^). Note that the preference-preference R^2^s of homeotherms vs. *Xenopus* are also ∼97% but *Xenopus*' motif preferences are less extreme (i.e., the slope is not 1.0).

### Synonymous-codon usage and the occurrence-preference relationship

To evaluate occurrence-preference correlations without direct effects due to selection on amino-acid usage, we show in Table 3 the occurrence-preference R^2^s and slopes for each individual 3-, 4-, and 6-fold family of trinucleotide motifs (and for the 4fold “subfamilies” of each 6fold family) as well as occurrence-preference slopes for each 2fold family or subfamily (but no R^2^s since these are always 100% with only two points). All chordate 3-, 4-, and 6-fold slopes are positive except for Arg4 (negative but very close to zero), and the corresponding R^2^s are mostly very high in homeotherms and moderate to high in the other chordates. Not surprisingly given the occurrence-preference results with all the 61 motifs, *Gallus* and *Xenopus* R^2^s are highest. The slopes for 2fold families are mostly positive, except for His ones which are mainly negative; and *Xenopus* 2fold slopes are often negative. Therefore for 3-, 4-, and 6-folds, the relative occurrences of codons within SC families are mostly very congruent with the corresponding motif preferences.

**Table 3:** Occurrence-preference R^2^s and slopes within degeneracy-defined trinucleotide families.

		4folds								6 folds			3fold
		*Leu4*	Val	*Ser4*	Pro	Thr	Ala	*Arg4*	Gly	*Leu*	*Ser*	*Arg*	Ile
R^2^s	*Homo*	**0.869**	**0.994**	0.789	0.684	**0.861**	0.606	0.166	0.611	0.774	0.762	0.496	**0.833**
	*oposs.*	**0.960**	**0.994**	**0.871**	**0.904**	**0.941**	**0.910**	0.062	**0.977**	**0.870**	**0.856**	**0.838**	**1.000**
	*platyp.*	0.653	**0.879**	0.440	0.024	0.183	0.250	0.242	0.485	0.630	0.430	0.467	0.621
	*Gallus*	**0.876**	**0.974**	**0.887**	**0.949**	**0.989**	**0.971**	0.100	**0.962**	0.743	**0.897**	**0.850**	**0.895**
	*Xenop.*	**0.923**	**0.929**	**0.895**	**0.972**	**0.983**	**0.990**	0.309	**0.865**	**0.817**	**0.901**	**0.923**	0.601
	*Danio*	0.786	**0.828**	0.740	0.790	**0.950**	0.784	0.050	**0.973**	0.763	0.693	0.784	**0.932**
	*Fugu*	**0.816**	**0.813**	0.385	0.227	0.177	0.226	0.207	0.163	0.768	0.330	0.600	**0.858**
	*Ciona*	0.296	0.694	0.437	0.605	0.629	**0.833**	0.719	**0.928**	0.263	0.338	0.666	0.031
slopes	*Homo*	0.0107	0.0092	0.0037	0.0028	0.0035	0.0049	0.0021	0.0077	0.0086	0.0041	0.0016	0.0171
	*oposs.*	0.0065	0.0066	0.0041	0.0037	0.0039	0.0052	**−0.0008**	0.0080	0.0053	0.0042	0.0024	0.0114
	*platyp.*	0.0160	0.0145	0.0031	0.0008	0.0030	0.0052	0.0028	0.0081	0.0127	0.0036	0.0024	0.0149
	*Gallus*	0.0093	0.0070	0.0038	0.0036	0.0036	0.0052	0.0007	0.0062	0.0069	0.0040	0.0025	0.0099
	*Xenop.*	0.0068	0.0059	0.0052	0.0043	0.0050	0.0055	0.0010	0.0085	0.0055	0.0052	0.0037	0.0066
	*Danio*	0.0087	0.0090	0.0034	0.0032	0.0035	0.0047	0.0004	0.0112	0.0076	0.0035	0.0022	0.0096
	*Fugu*	0.0198	0.0180	0.0035	0.0016	0.0028	0.0048	0.0023	0.0040	0.0173	0.0042	0.0032	0.0222
	*Ciona*	0.0030	0.0062	0.0113	0.0067	0.0071	0.0127	0.0167	0.0152	0.0034	0.0102	0.0174	0.0022
		2folds											
	(R^2^ = 1)	Phe	*Leu2*	Tyr	*His*	*Gln*	Asn	Lys	Asp	Glu	*Cys*	*Ser2*	*Arg2*
slopes	*Homo*	0.010	0.003	0.033	**−0.012**	0.016	**−0.013**	0.009	0.013	0.025	0.010	0.012	0.000
	*oposs.*	**0.000**	0.003	**−0.030**	0.004	0.014	0.019	0.000	**−0.016**	**−0.005**	0.023	0.003	0.003
	*platyp.*	0.029	0.005	**−0.379**	**−0.019**	0.027	**−0.104**	0.008	0.078	0.017	0.030	0.017	**0.000**
	*Gallus*	**−0.011**	0.002	0.015	**−0.010**	0.015	0.114	0.003	**−0.132**	0.011	0.003	0.006	0.002
	*Xenop.*	0.200	0.002	0.013	0.003	0.010	**−0.046**	**−0.011**	**−0.251**	**−0.014**	0.131	0.004	0.004
	*Danio*	0.001	0.003	0.028	**−0.009**	0.012	0.010	**−0.002**	0.004	0.016	0.004	0.008	0.004
	*Fugu*	**−0.048**	0.006	0.104	**−0.057**	0.022	0.032	0.070	0.052	0.033	**−0.006**	0.028	**−0.005**
	*Ciona*	**−0.038**	0.001	0.052	**−0.017**	0.012	0.000	**−0.023**	0.027	0.022	0.025	**−0.029**	0.014

### Amino acid composition and the occurrence-preference relationship

In Table 4 we show occurrence-preference R^2^ and slopes for the twenty amino acids together and for the various motif subgroups. In *Homo* there is only a 1% R^2^ and a very small slope for the correlation of the whole-genome occurrences of the twenty amino acids with the twenty corresponding sums of motifs preferences. When the 4- and 6-fold-degenerately encoded amino acids and the corresponding motif sums are examined separately, however, *Homo* R^2^s are 57 and 95%, respectively (and 69% for the 2f-3aas group) and the slopes are much higher. Also across species do the 4fold, 2f-3aas, and 6fold groups have high R^2^s and slopes, with the 6folds of homeotherms, *Gallus*, and *Xenopus* showing very high 95%+ values, and platypus behaving unlike mammals (which the platypus patterns do also in the previous tables and figures). The high occurrence-preference R^2^s for these three groups indicate that motif preferences affect the whole-genome occurrence of encoded amino acids, i.e., not only that of codons, with the occurrences of the 6-fold-degenerate amino acids Leu, Ser, and Arg being especially correlated with the three corresponding sums of six motif preferences each.

**Table 4:** Occurrence-preference R^2^s and slopes after pooling values according to encoded amino acid.

	R^2^ values								slopes							
	*Homo*	oposs.	platyp.	*Gallus*	*Xenop.*	*Danio*	*Fugu*	*Ciona*	*Homo*	oposs.	platyp.	*Gallus*	*Xenop.*	*Danio*	*Fugu*	*Ciona*
*all*	1%	1%	0%	1%	4%	2%	2%	16%	0.001	0.001	0.001	0.001	0.002	0.002	0.002	0.008
*2folds*	1%	6%	9%	0%	0%	1%	0%	2%	0.001	0.003	0.004	0.000	−0.001	−0.001	0.001	0.002
*4folds*	57%	37%	29%	19%	1%	58%	8%	36%	0.004	0.002	0.003	0.003	0.000	0.008	0.003	0.013
*2f-3aas*	69%	67%	51%	71%	71%	59%	48%	63%	0.014	0.011	0.011	0.012	0.012	0.010	0.012	0.012
*6folds*	95%	98%	78%	93%	98%	84%	79%	78%	0.005	0.004	0.006	0.005	0.006	0.005	0.008	0.012

Table 5 shows that the twenty amino-acid occurrences and the twenty corresponding sums of preferences are quite conserved across species and that the most conserved sums are those of 2folds and 6folds, their R^2^s averaging 96%+ within vertebrates and 86%+ in chordates. The 99%+ occurrence-occurrence R^2^s for 6folds in chordates is simply astonishing given that the R^2^s for the corresponding eighteen individual-codon frequencies (rather than for the three sums) is very low at times (e.g., about 10% in *Ciona*-vertebrate comparisons), indicating that the whole-proteome 3-term ratio Leu/Ser/Arg is rigidly conserved during evolution even when the whole-genome occurrences of the corresponding eighteen 6fold codons differ drastically across genomes. The correlations of the three sums of 6fold-motif preferences show a similar if weaker trend, when compared to those for the eighteen individual preferences. For instance the *Ciona*-vertebrate preference-preference R^2^s average ∼32% for the eighteen preferences (Table 2) but ∼94% for the three sums of preferences in Table 5, indicating that the motif preferences for 6fold motifs may be also tuned to deliver similar supervenient pooled results.

**Table 5:** R^2^s and slopes of the correlations between amino-acid occurrences or between corresponding sums of preferences.

		whole-genome amino acid occurrences								sums of corresponding motif preferences							
		*Homo*	*oposs.*	*platyp.*	*Gallus*	*Xenop.*	*Danio*	*Fugu*	*Ciona*	*Homo*	*oposs.*	*platyp.*	*Gallus*	*Xenop.*	*Danio*	*Fugu*	*Ciona*
all 20 amino acids	*Homo*		0.918	0.988	0.938	0.878	0.918	0.950	0.741		1.097	0.642	1.000	0.888	0.810	0.496	0.351
	*oposs.*	98%		1.055	1.011	0.960	0.991	1.011	0.836	97%		0.579	0.875	0.788	0.705	0.436	0.313
	*platyp.*	99%	98%		0.946	0.894	0.928	0.962	0.768	89%	89%		1.356	1.206	1.143	0.735	0.485
	*Gallus*	98%	98%	98%		0.940	0.981	0.996	0.821	97%	92%	83%		0.869	0.819	0.487	0.360
	*Xenop.*	94%	97%	96%	97%		1.018	1.039	0.894	98%	95%	83%	96%		0.884	0.533	0.409
	*Danio*	97%	97%	97%	99%	97%		1.013	0.842	85%	80%	78%	89%	82%		0.595	0.415
	*Fugu*	98%	96%	99%	97%	96%	97%		0.800	86%	83%	88%	86%	81%	96%		0.666
	*Ciona*	77%	85%	82%	86%	92%	87%	83%		66%	65%	58%	71%	72%	71%	68%	
2folds	*Homo*		0.918	0.911	0.968	0.717	0.948	0.801	0.557		0.946	0.666	1.071	0.838	1.118	0.688	0.435
	*oposs.*	97%		0.972	1.034	0.780	1.002	0.840	0.641	92%		0.674	1.033	0.792	1.082	0.680	0.407
	*platyp.*	99%	97%		1.062	0.796	1.041	0.876	0.642	80%	80%		1.263	0.947	1.377	0.900	0.568
	*Gallus*	98%	97%	99%		0.751	0.981	0.820	0.617	99%	89%	76%		0.788	1.019	0.625	0.413
	*Xenop.*	93%	96%	97%	98%		1.269	1.051	0.876	95%	82%	67%	97%		1.217	0.746	0.517
	*Danio*	97%	94%	99%	99%	96%		0.838	0.619	93%	85%	78%	89%	81%		0.611	0.337
	*Fugu*	98%	93%	98%	98%	93%	99%		0.694	92%	87%	87%	88%	80%	97%		0.560
	*Ciona*	61%	70%	68%	71%	83%	70%	62%		82%	70%	78%	86%	86%	66%	70%	
4folds	*Homo*		0.458	0.851	0.610	0.156	0.244	0.547	−0.957		1.129	0.981	0.590	0.765	−0.006	0.222	−0.268
	*oposs.*	63%		1.309	0.756	0.341	0.348	0.851	−1.201	87%		0.869	0.333	0.566	−0.042	0.221	−0.163
	*platyp.*	89%	70%		0.720	0.224	0.393	0.711	−0.873	83%	96%		0.339	0.620	0.025	0.301	−0.212
	*Gallus*	45%	23%	51%		0.409	0.772	0.762	0.064	66%	31%	25%		0.925	−0.007	0.043	−0.353
	*Xenop.*	13%	21%	22%	76%		1.707	1.427	1.264	96%	77%	73%	74%		0.013	0.255	−0.329
	*Danio*	10%	7%	21%	81%	87%		0.804	0.766	0%	2%	0%	0%	0%		0.575	−0.458
	*Fugu*	54%	43%	74%	87%	67%	71%		−0.159	29%	42%	62%	1%	24%	32%		−0.506
	*Ciona*	49%	26%	33%	0%	16%	19%	1%		57%	31%	41%	52%	52%	27%	34%	
2f -3aas	*Homo*		0.931	0.874	0.917	0.643	0.881	0.765	0.421		1.057	0.953	1.111	0.741	1.043	0.678	0.615
	*Oposs.*	96%		0.912	0.958	0.695	0.906	0.777	0.512	84%		0.749	0.857	0.537	0.857	0.563	0.416
	*Platyp.*	99%	97%		1.043	0.740	1.002	0.871	0.504	90%	74%		1.040	0.695	1.054	0.712	0.611
	*Gallus*	99%	98%	99%		0.711	0.960	0.829	0.496	99%	79%	88%		0.673	0.927	0.600	0.562
	*Xenop.*	93%	99%	96%	97%		1.292	1.104	0.784	93%	65%	83%	95%		1.231	0.801	0.852
	*Danio*	98%	93%	98%	99%	93%		0.867	0.496	89%	79%	92%	87%	73%		0.665	0.505
	*Fugu*	98%	91%	98%	97%	90%	99%		0.524	83%	76%	93%	80%	68%	98%		0.746
	*Ciona*	46%	61%	51%	54%	71%	50%	43%		85%	51%	84%	88%	96%	70%	69%	
6folds	*Homo*		1.007	0.963	1.064	1.092	0.986	0.927	0.831		1.116	0.632	1.037	0.881	0.779	0.449	0.264
	*Oposs.*	100%		0.950	1.054	1.087	0.981	0.920	0.826	100%		0.565	0.928	0.790	0.694	0.400	0.235
	*Platyp.*	100%	99%		1.102	1.127	1.017	0.957	0.858	95%	95%		1.589	1.333	1.253	0.726	0.429
	*Gallus*	100%	100%	100%		1.028	0.928	0.872	0.782	100%	99%	98%		0.846	0.763	0.441	0.259
	*Xenop.*	99%	100%	98%	99%		0.903	0.846	0.760	100%	100%	96%	100%		0.886	0.511	0.300
	*Danio*	99%	100%	98%	99%	100%		0.937	0.841	92%	91%	99%	95%	92%		0.580	0.344
	*Fugu*	100%	100%	99%	100%	100%	100%		0.897	91%	90%	99%	94%	91%	100%		0.593
	*Ciona*	99%	100%	99%	100%	100%	100%	100%		89%	88%	98%	93%	89%	100%	100%	

### Statistical issues raised by the occurrence-preference relationship

The results present so far allow one to evaluate more specifically which statistical concerns are raised by the nature of our datasets, analyses, and conclusions; and which ones may be of lesser concern. This is why we chose to discuss this issue here before continuing with the presentation of results, rather than in the Materials and Methods.

Whole-genome codon frequencies are based on the whole sampling universe so that a sampling variance is counterfactual and negligible anyway given that i) most datasets contain 12,000 genes or more, i.e., millions of codons, and that ii) each “average motif preference” is based on tens of thousands of individual-gene motif preferences. Furthermore, the two human datasets of genes with known transcripts or known mouse homologues comprise ∼12,000 genes each and provide therefore a *de facto* 30% jackknifing for the conclusions drawn from the 33,860-gene human dataset; and non-surprisingly the results are identical (see also below). The evolutionary variance, on the other hand, could be a problem but this possibility is “academic” at least within mammals given that i) the various mammalian codon occurrences correlate with each other with R^2^s close to 90% (98% among placentals) and ii) that the preferences for across-codon motifs are highly correlated not only within mammals but also between the latter, *Gallus*, and *Xenopus* (98%) despite extensive 3rd-position divergence between lineages (the bases at 3rd-positions determine motif preferences). Therefore neither codon counts nor motif preferences have a sampling or an evolutionary variance that could lead to spurious patterning by chance so that their conservation or divergence must reflect deterministic effects (see, e.g., the sections below on GC content “effects”).

The independence between individual codon frequencies is quite high since there are 61 codons (and the results are identical if one uses total codon counts instead of frequencies). However, many across-codon motif preferences are instead strongly correlated with one another. It is unclear to us whether this non-independence could compromise our analyses and conclusions at least as far as the relationship between codon occurrences and across-codon motif preferences is concerned. On the contrary, it is reasonable to argue that because no plotted point has substantial estimation error on either axis, i.e., because no point has a real chance of appearing in a spot very different from the one it actually occupies in a plot, across-codon preferences can therefore be used to predict genomic codon occurrences, leaving the explanation of the remaining “variance” to unknown effects to be incorporated later. A related and possibly more serious problem is the presence of wrongly diagnosed genes in the datasets. Below, we dealt with this by comparing the full-genome results from *Homo* and *Mus* with results obtained using the two sets of 12.277 genes that are homologous in the two species. Another issue, but concerning biological interpretation, is the extent to which the sequence of each individual gene is shaped by the forces behind motif preferences; in this paper we focus on whole-genome and large-group trends and make no attempt at addressing this issue.

### The occurrence-preference relationship as a function of coding-region GC content

**Figure 3:**
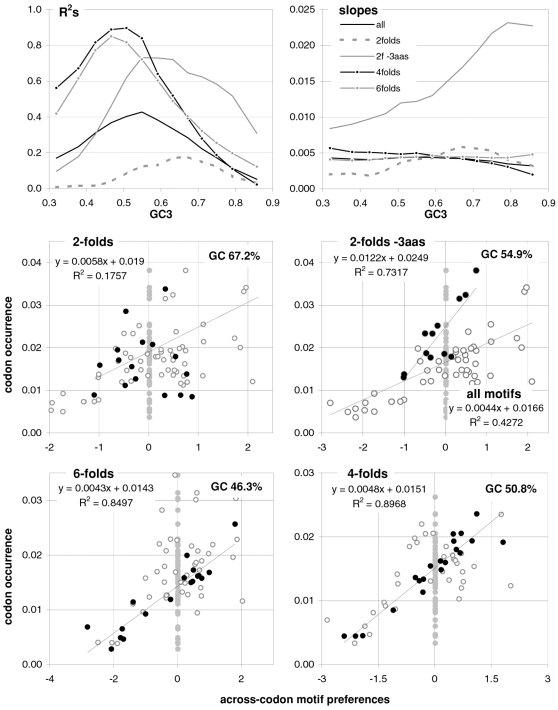
Codon occurrences vs. trinucleotide-motif preferences as a function of GC content. At the top are plotted the R^2^s and the slopes of the correlations between codon occurrences and corresponding motif preferences for given values of 3^rd^-position GC content (GC3). The other plots show the situation at the GC3 values delivering peak R^2^s for, clockwise, 2 fold, 2fold-3aas, 4fold, and 6fold codons/motifs (i.e., 0.67, 0.55, 0.46, and 0.51 GC3; the GC3 delivering the peak all-motifs R^2^ coincides with that of 2folds-3aas; see also Figure 1). The 33,860 human coding regions were sorted according to 3^rd^-position GC content and subdivided into 13 groups of equal size.

Since GC content can vary markedly along chromosomes [1], we show in Figure 3 how the correlation between codon occurrences and trinucleotide motif preferences reacts to increasing GC content in human coding regions. The 33,860 human genes were sorted by increasing GC content at their third codon positions (GC3) and separated into thirteen groups having equal number of sequences, in order to calculate occurrence-preference R^2^s within each group as it was done with the whole-genome dataset to generate Figure 1. Almost without exception the R^2^s change smoothly with increasing GC3, with the peak R^2^ for all 61 motifs being 43% at 0.55 GC3 and those for 2folds, 2f-3aas, 4folds, and 6folds being 18, 73, 90, and 85% at 0.67, 0.55, 0.51, and 0.47 GC3, respectively. The corresponding plot of the slopes vs. GC3 in the top right of the same figure, shows that only the slopes of 2folds and especially those of 2f-3aas react strongly to GC3. The occurrence-preference plots for the GC3 values that deliver the highest R^2^s show that the various relationships are similar to the whole-genome ones in Figure 1. All in all, the monotonic reaction of the R^2^s to GC3 indicates that the whole-genome patterns presented above are indeed “noisy” but in a biologically structured way.

**Figure 4:**
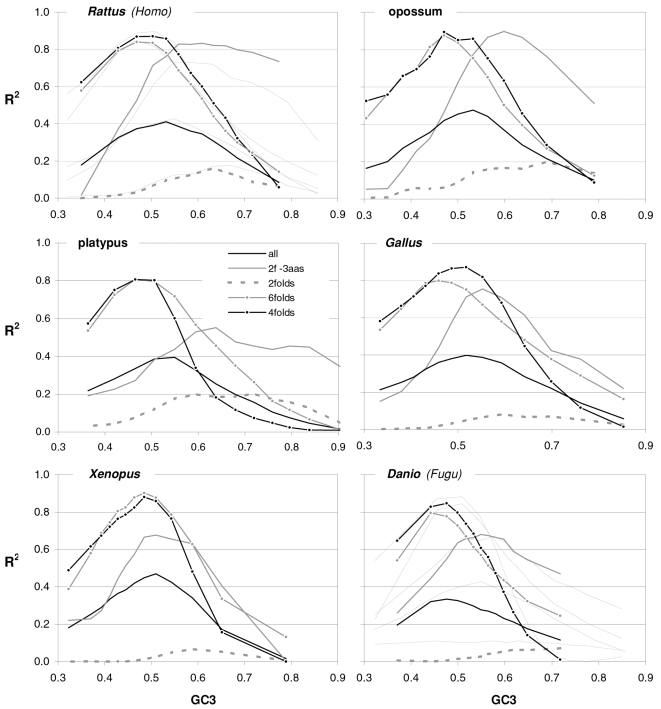
Occurrence-vs.-preference R^2^s in vertebrates as a function of GC3. The R^2^s of the correlation between codon occurrences and corresponding off-frame-motif preferences for increasing GC3 values in the genomes of *Rattus*, opossum, platypus, *Gallus*, *Xenopus*, and *Danio*. In the *Rattus* plot the *Homo* patterns from Fig. 3 are used as background and *Fugu's* are in the background in the *Danio* plot.

**Figure 5:**
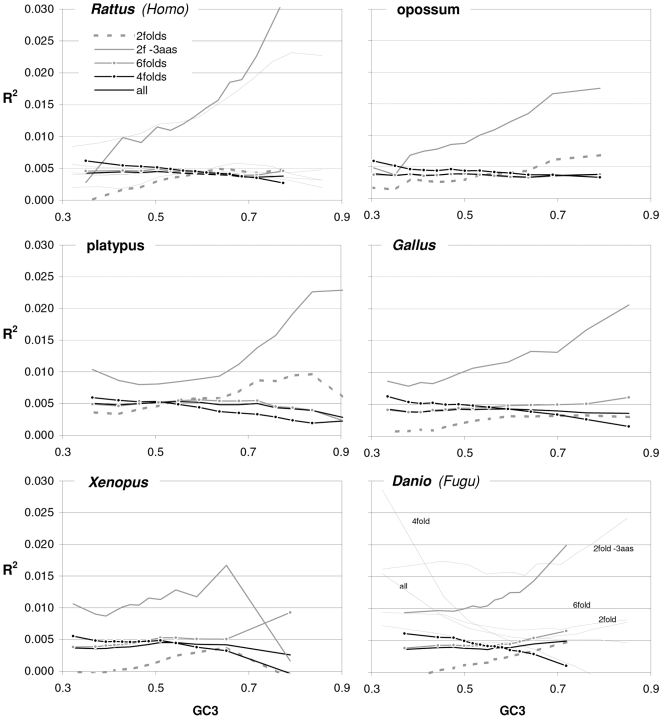
Occurrence-preference slopes in vertebrates as a function of GC3. The slopes of the correlation between codon occurrences and corresponding motif preferences for increasing GC3 values in the genomes of *Rattus*, opossum, platypus, *Gallus*, *Xenopus*, *Danio*, and *Fugu*. In the *Rattus* plot the *Homo* patterns from Fig. 3 are used as background and *Fugu*'s are in the background in the *Danio* plot.

In Figures 4 and 5 we show how occurrence-preference R^2^s and slopes change with GC3 in the other vertebrate genomes. The R^2^ patterns are remarkably similar across homeotherm vertebrates. The jagged opossum patterns may be due to the inclusion of falsely diagnosed (or newly recruited) genes in the dataset we used. Indeed similar irregularities were also seen with *Mus* and *Canis*, but in the *Mus* case disappeared when using genes with known transcripts or known human homologues (neither is shown; occurrence-preference R^2^s in non-coding DNA are almost 100% at 0.5 GC and react more strongly to GC changes, see below). Also here platypus' patterns seem fish-like to some extent. An interesting pattern is that the all-motifs and the 6fold lines cross above 0.7 GC3 in homeotherms (including *Mus* and *Canis*, not shown) but clearly below 0.7 in poikilotherms. *Danio* R^2^ patterns are more homeotherm-like than *Fugu*'s (which were relegated to the background). Note that the 2f-3aas R^2^s from high-GC rodent genes (in all rodent datasets) are much higher than those from the other vertebrates expect the opossum and may indicate a difference in genome-wide amino-acid selection (see also below). Also the slopes' reaction to GC3 in Figure 5 is remarkably similar across vertebrates albeit less strikingly so than the R^2^s; and also here are the patterns obtained with known-homologue *Rattus* and *Mus* genes clearly smoother than those from the whole-genome dataset. Remarkably, *Danio*'s slopes are homeotherm-like in many respects unlike *Fugu*'s which, like its R^2^s, are patterned unlike those of the other vertebrates. Finally, it is hard to believe that the highest-GC *Xenopus* group may not be anomalous since it delivers R^2^s and slopes unlike those of any vertebrate.

### Coding-region GC and the balance between the preferences for GC-rich and GC-poor motifs

**Figure 6:**
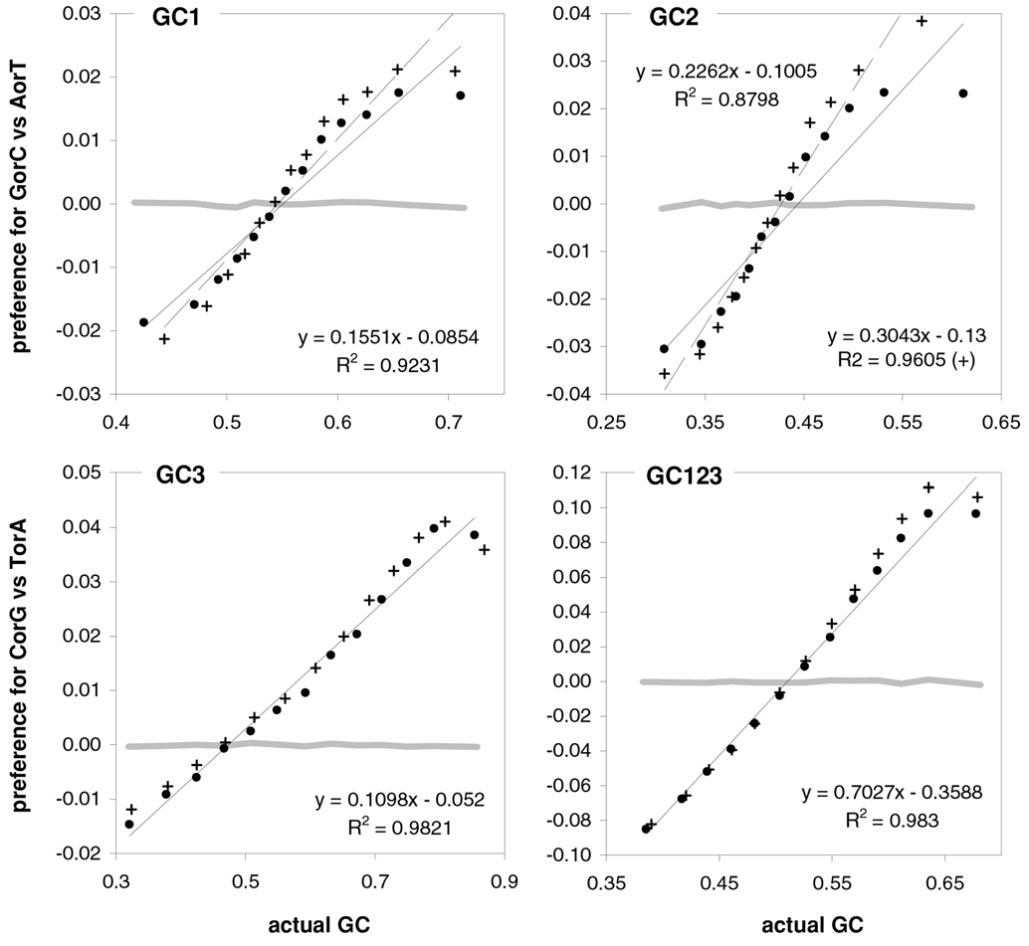
Coding-region GC vs. “GC-vs.-AT pressures” derived from motif preferences. The correlation between the GC content at the three codon positions of human genes and the sum of the preferences for across-codon trinucleotides weighted by their GC content at relevant places (see Methods). Solid dots are for the 33,860 human coding regions which were sorted by increasing GC content at the relevant codon position(s) and subdivided into 13 groups of equal size; +'s are for the 12,717 human genes with known mouse homologues. The thick horizontal grey lines are for a “null” data set created by randomizing the location of SCs in each of the 33.860 sequences before estimating motif preferences.

**Figure 7 pone-0002145-g007:**
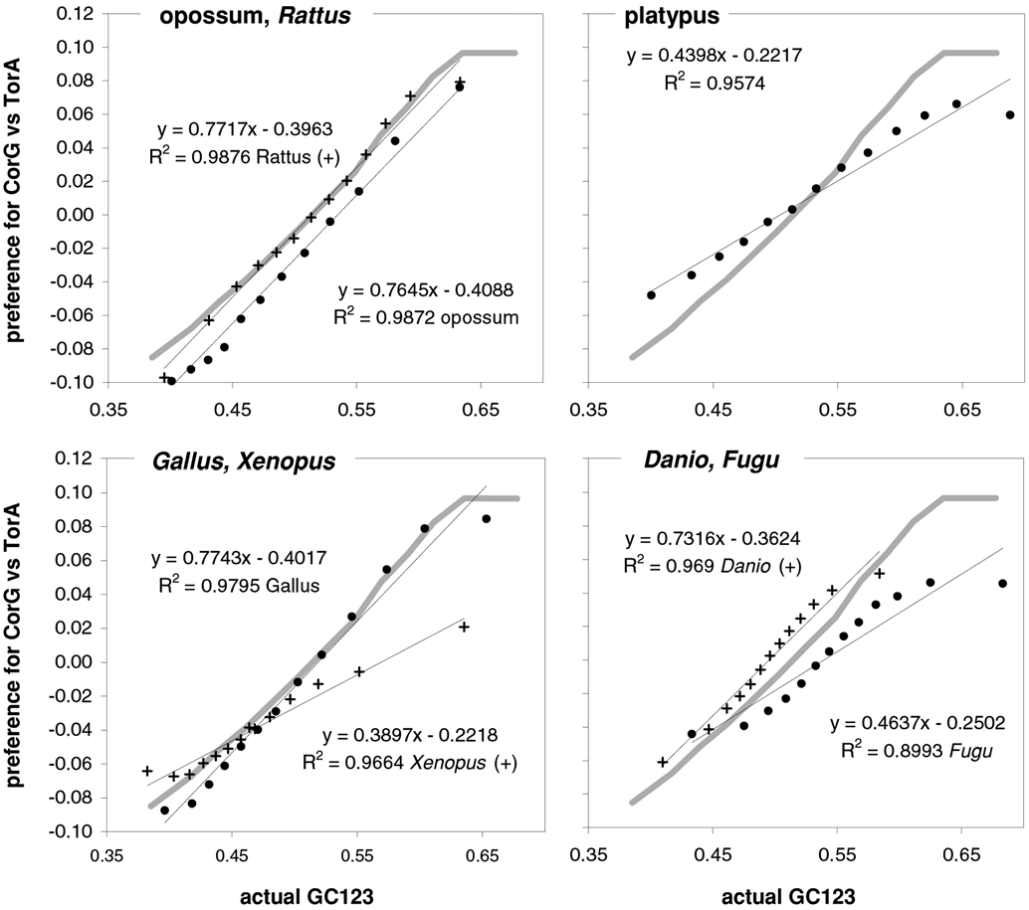
Vertebrate GC3 vs. GC3-pressures derived from motif preferences. The correlation between coding-region GC content (GC123) and the corresponding GCvsAT pressure derived from motif preferences. Clockwise from the top left are results for opossum and *Rattus* (+), platypus, *Gallus* and *Xenopus* (+), and *Fugu* and *Danio* (+). The thick gray lines are for human genes. See also Methods and the previous figure.

**Figure 8 pone-0002145-g008:**
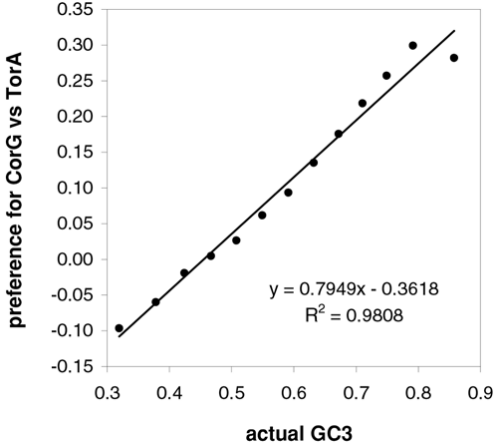
Vertebrate 3^rd^-position GC content and dinucleotide-motif preferences. The correlation in human coding regions between 3^rd^-position GC content and the GCvsAT pressure derived from dinucleotide-motif preferences (see also Methods and previous figures).

As mentioned in the Introduction and in the Materials and Methods, we found that GC content and motifs preferences are correlated strongly, positively, and linearly. In Figure 6 we plot the GC content at the three codon positions for each of the aforementioned thirteen groups of GC-sorted human genes against the corresponding GCvsAT pressures (see Materials and Methods) derived from the various preferences for the trinucleotide motifs in the same groups. The R^2^ for 3^rd^-position GC content is 98% (99% excluding the group with highest GC) and those for total, 1^st^-position, and 2^nd^-position GC are 98, 92, and 88%. The plotted horizontal “null” relationship was obtained by randomizing the SC location of each gene before motif-preference estimation, which leaves GC content unchanged but randomizes each gene's motif preferences, resulting in identical, zero-valued GC pressures in each of the thirteen groups. In Figure 7 we present the other vertebrates' plots of GC123 vs. the total GC pressure. The R^2^s are again striking, falling below 95% only in *Fugu* (90%). When using the GC pressure derived from dinucleotide preferences and genes sorted by GC3, the R^2^ is 98% (Fig. 8). Remarkably, for the human case no exclusion of individual di- or tri-nucleotide preferences –or of pairs thereof– from the GC-pressure sums lowers the R^2^ below 89%, with CC, AA, CTT, CCC, CAA delivering the top reductions (not shown).

**Figure 9 pone-0002145-g009:**
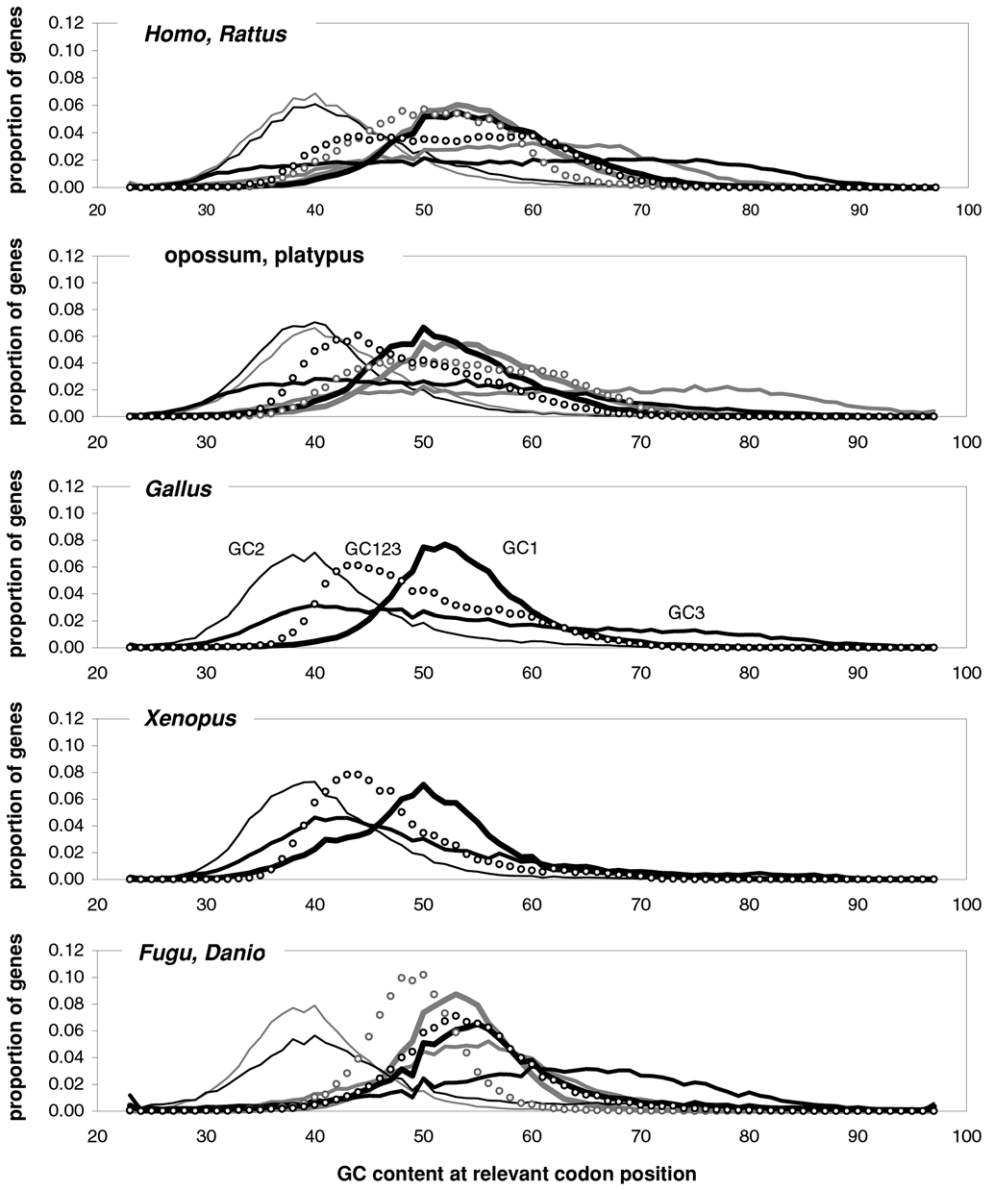
Coding-region GC content in vertebrates. The distribution of the GC content at the three codon positions in vertebrate genes. Thickest black line: first-position GC content (GC1); 2^nd^-thickest black line: GC2; thin black line: GC3; circles: GC123. Lighter lines and circles are for *Rattus*, platypus, and *Danio*, respectively.

Figure 7 shows indirectly that the GC3 excursion is quite similar across vertebrates, indicating that the GC content of vertebrate genes and their proximities may be quite similar (since GC3 is well-known to be highly correlated to the GC content of the genomic regions surrounding genes [1]). This motivated Figure 9 that shows the distribution of the various GC contents in vertebrate coding regions. Remarkably, *Xenopus* GC distributions are very homeotherm-like although its GC3 distribution lacks mass, but not presences, when GC3 is low (consistent with *Fugu*'s lack of “junk” DNA which tends to be AT-rich). *Danio*'s GC3 mass tends to be in the middle of the homeotherm range (and looks like the homeotherm GC123). In general, GC3 excursions are not very different across vertebrates although the low-GC tails tend to be thicker in homeotherms. Note also that the bimodality of human GC123 is almost identical in *Canis* and that the same applies to GC1, GC2, and GC3 (not shown; hence GC content cannot be what makes us human). Finally, note that in all vertebrates GC123 tends to fall between 0.37 and 0.70.

The above similarities among the GC content distributions of vertebrates and especially between warm- and cold-blooded vertebrates indicates that vertebrates may share an effectively similar, mechanistically homologous, and phylogenetically quite conserved distribution of mutational intensities in genic DNA (although homologous genes can have quite different GC content in different vertebrates). This together with the fact that the GC contents of genic DNA in general, and of third codon-positions in particular, is well-known to be highly correlated with the GC content of the regions in which genes are embedded [1], indicates that the existence along vertebrate chromosomes of “isochores” [1], or the lack thereof, may be a simple consequence i) of how the DNA metabolism of non-genic “junk” DNA differs across lineages (since that of genic DNA appears to be very conserved across lineages) and ii) of how much “junk” DNA in each different lineage is sufficiently interspersed with genic DNA to be subjected to near-genic DNA-metabolism. We will see in the Discussion, when dealing with the molecular-biological foundations of the heterogeneities shown by NBDM across GC contents, that major DNA-metabolism differences exist between gene-rich and gene-poor regions, and that the distribution of evolutionarily tuned mutational intensities in gene-rich DNA is very likely to be unaffected by distantly located “junk” DNA in the same or other chromosomes, or the lack thereof, exactly as indicated by the similarity of the distributions of genic vertebrate GC content shown in Figure 9 (although newly inserted junk DNA can disrupt transiently the compaction of a region's chromatin and hence also the local DNA metabolism; see Holmquist and Ashley [20] and citations therein).

The main message implied by the strikingly linear relationship between the GCvsAT pressures and GC content evident in Figures 7 and 8 is, however, that changes in a highly amorphous summary statistic like GC content –which one would be tempted to consider as “a good place to start” because of its simplicity– could be generated by forces acting in a non-uniform way on more complex entities like individual nucleotide motifs whose joint occurrence can by chance end up being concerted in a way that allows the summary statistic to behave in an appealing way, prompting unjustified efforts to find a mechanistically simple force that can explain the summary statistic's “simple” dynamics and hindering thereby the identification of the actual underlying forces. In other words, Occam's razor could be misleading in this case.

### Coding-region GC and changes in the strength of motif-preferences

**Figure 10:**
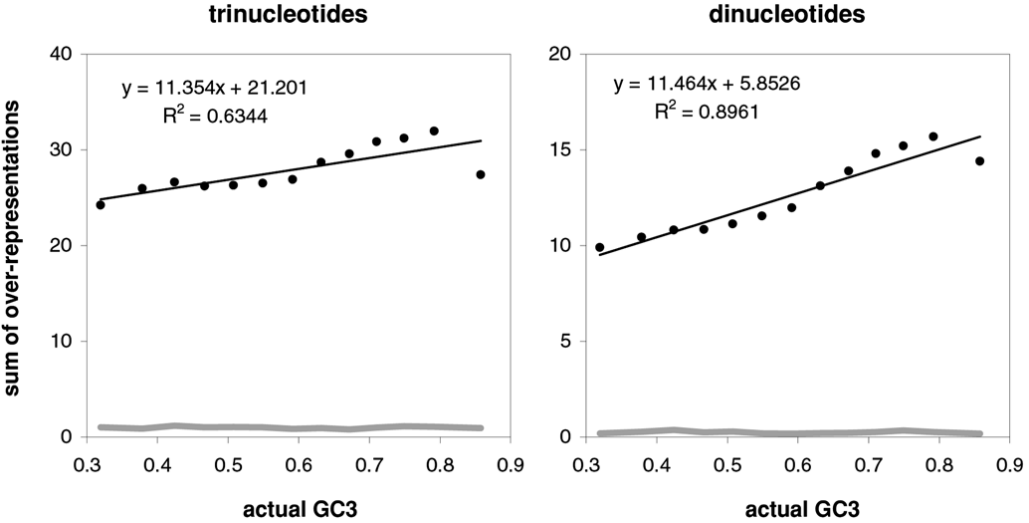
The non-randomness of motif preferences as a function of GC content. The correlation between GC3 in human genes and the sum of all the *over*-representations of tri- or dinucleotide motifs (left, right). The gray lines are the patterns from the null human data set. Note that using the sum of the absolute value of *every* motif preference delivers lower R^2^s of 53.6 and 84.3%.

The strong correlations between the preference-derived GCvsAT pressure and GC content presented above prompted us to examine additional correlations of GC content with other summary statistics that can be derived from motif preferences (strand asymmetries are shown further below). In Figure 10 we plot the summary statistic that gave the strongest relationship, i.e., the sum of the preferences for *over*-represented tri- or di-nucleotides (left, right). This sum gives very high 63 and 90% R^2^s with GC3, respectively (while the sum of the absolute values of *every* motif preference gives relatively lower 54 and 84% R^2^s). These high R^2^s are, however, not as high as those connecting preference-based GC pressures to GC content. Note furthermore that the two slopes are almost identical for tri- and di-nucleotide over-representations, perhaps indicating comparable levels on non-randomness in tri- and di-nucleotide preferences (as observed, albeit not as cleanly, when comparing the slopes of the correlations of preference-derived GC pressures with GC content).

A important indication given by Figures 7 to [Fig pone-0002145-g008] [Fig pone-0002145-g009] 10 concerns the validity of the conclusion that the forces behind motifs preferences may shape vertebrate-DNA primary structure only or mainly when GC content is intermediate, simply because occurrence-preference correlations are highest when GC content is intermediate. Figures 6–8 and 10 show instead that preference-derived summary statistics correlate with GC content over the whole range of GC contents shown, although they are fully independent from GC content. Therefore the processes that generate motif-preference heterogeneities could very well determine GC-content heterogeneities as a side effect, even when both the preferences happen not to correlate strongly with motif occurrences at the motif-by-motif level and the base composition would seem to “account for” motif occurrences quite well. Figure 10, e.g., shows that motif preferences are stronger when GC is very high, i.e., when motif occurrences and preferences do not correlate very highly. Therefore the strikingly high preference-occurrence correlations observed when GC content is close to 0.5 –that reach 99% in non-genic and intronic DNA (below)– may be due to the fact that when GC content is intermediate the primary-structural dynamics are favorable to the occurrence-preference correlation. However, as Figure 10 indicates, these high correlations do not imply at all that the preference-generating forces active in intermediate-GC DNA are stronger than those active in lower- or higher-GC DNA, at least as far as the extremeness of the individual preferences reflects the strength of these forces. Below, however, we will show that NBDM affects strongly the quality and quantity of the amino-acid replacements that point mutations generate so that it may not be a coincidence that occurrence-preference correlations are highest when GC content is intermediate, since that is the GC of most genes.

### Individual trinucleotide occurrences and preferences as a function of GC content

**Figure 11:**
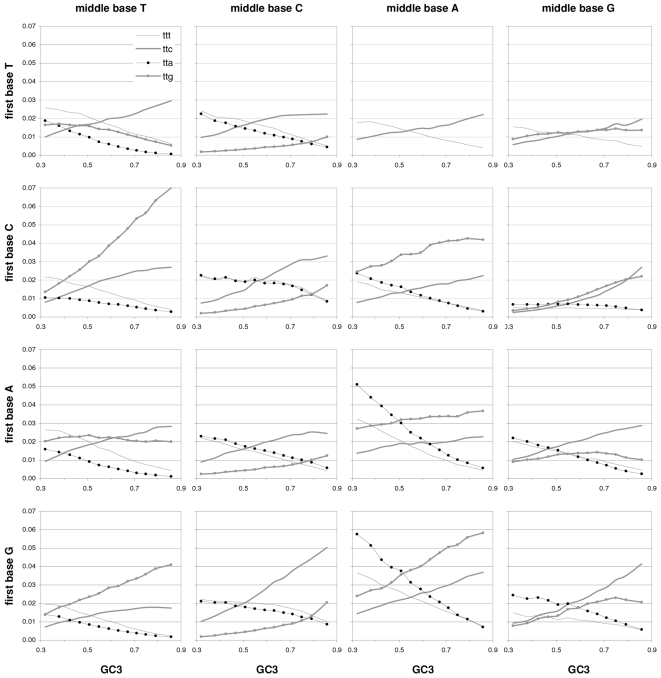
The occurrence frequency of the 61 sense codons as a function of increasing 3rd-position GC content. In each plot the third base is labelled as in the top left (stop codons not shown).

**Figure 12:**
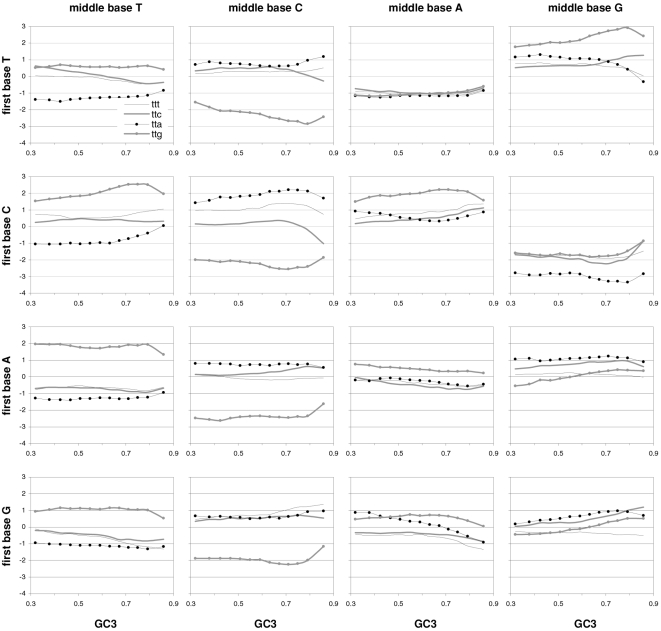
The across-codon preferences for the 64 trinucleotide motifs as a function of increasing GC3. In each plot the third base is labelled as in the top left.

**Figure 13:**
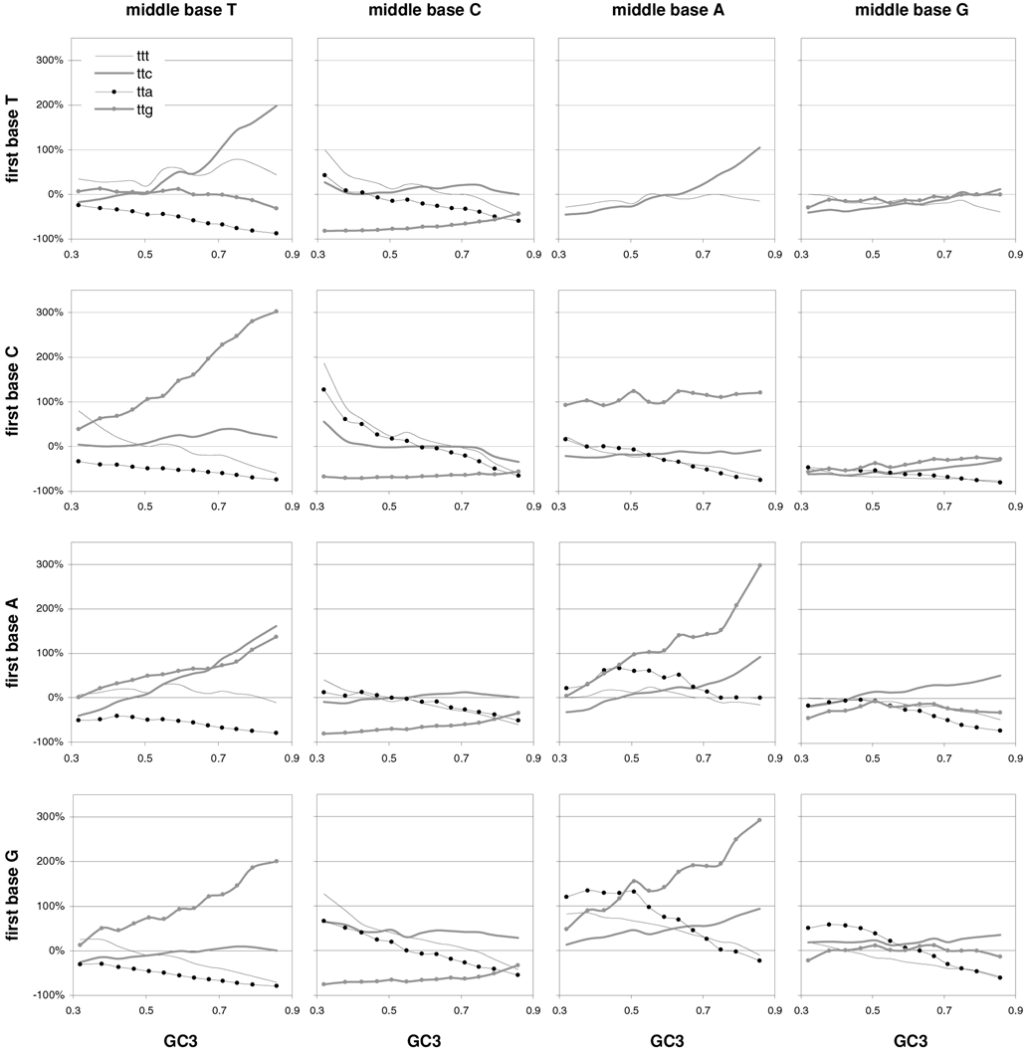
GC content and the departure of codon occurrences from base-composition expectations. The difference between the occurrence of each of the 61 sense codons from the occurrence expected given the base composition that best fits the total codon occurrences in each GC3-sorted group of coding regions, plotted by increasing GC3. In each plot the third base is labelled as in the top left.

The results above using groups of CDSs sorted by GC motivated us to study how individual codon frequencies and trinucleotide motif preferences change with increasing GC3 (Fig. 11 and 12). Additionally, to gain a better understanding of motif preferences we present in Figure 13 the departure of each individual codon occurrence from that expected given the base composition which fits best the codon occurrences in each GC3-sorted group (i.e., that minimizing the sum of the every absolute difference between an observed and a predicted codon frequency, with granularity 0.001). The lowest occurrences are those of trinucleotides containing CG and TA dinucleotides; furthermore their reaction to changing GC3 is quite restricted. Another remarkable trend is that all T- and A-ending codons decrease in frequency with GC3, CGT being the only exception (always low), which contrasts clearly with the often large differences among the increases of C- and G-ending codons. Of the latter the G-ending codons are those with most variable behavior while C-ending ones increase monotonically with higher GC3 almost without exception. The most extreme increases in occurrence in response to higher GC3 are those of CTG, GCC, and GTG, and the largest decreases are those of AAA and GAA. Note that these results for plain codon occurrences, and similar ones below for dinucleotides, suffice to conclude that neither GC content nor base composition can possibly account for the occurrence of individual codons in groups comprising many coding regions (where the fact that the contrasted groups contain many genes makes it hard to invoke constraints arising from the need of individual genes to encode peculiar proteins).

The reactions to GC3 of the occurrences of single trinucleotides are not accompanied by corresponding reactions by motif preferences. Indeed most motif preferences change only subtly with GC3, especially for high GC3 values (see below the situation at the dinucleotide level). This lack of reaction is surprising given how remarkably linearly the preference-derived GCvsAT pressures relate to GC content (see above). In contrast, the reaction to GC3 of the departure of the occurrences from their base-composition expectations is often similar to the corresponding reactions of the occurrences of single trinucleotides, even though 17 out of 61 occurrences change linearly with GC3 without concurrent changes in occurrence departure from expectation (Fig. 13). Note also the almost total lack of congruence between the way in which preferences react to GC3 and how occurrences depart from their base-composition expectations. This, together with the similar observation for dinucleotides at the end of the next section, shows that the randomization of SC location delivers motif preferences that are neither related circularly to motif occurrences nor replaceable in an obvious way by the departure of motif occurrences from their base-composition expectations.

### Individual dinucleotide occurrences and preferences as a function of GC content

**Figure 14:**
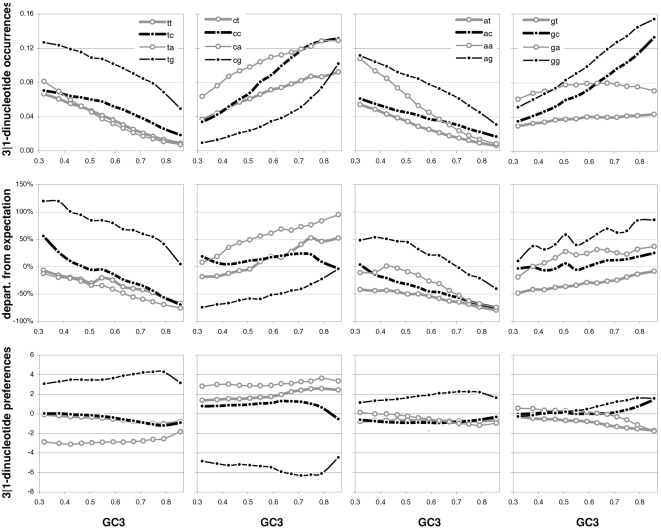
Dinucleotide occurrences and preferences as a function of GC content. From top to bottom are the occurrence frequency of the 16 across-codon (3∥1) dinucleotides, their departure from the expectation given the base composition that best fits codon occurrences (divided by the expectation), and their across-codon preferences, as a function of increasing GC3.

Changes in the occurrences of, and in the preferences for, across-codon dinucleotides as a function of GC3 are shown in Figure 14. In general the occurrence of motifs beginning with TorA decreases with increasing GC3 while that of those beginning in CorG increases, which is consistent with these being 3rd-position TorAs and CorGs. Remarkably and almost without exception, when looking at pairs of dinucleotides ending in TorA or CvsG but sharing the first nucleotide, when a dinucleotide is less preferred than its partner, its occurrence is always lower than that of its partner across all GC3 groups. As it was the case for trinucleotides, occurrence changes are not clearly mirrored by similar changes in preferences, which is surprising given that, as it was the case for trinucleotides, the GCvsAT pressure derived from dinucleotides preferences showed a remarkably linear increase with GC3 (see Fig. 8). However, occurrence changes with GC3 are mirrored by changes in the departure of the occurrences from their corresponding base-composition expectations although not always as smoothly or with similar steepness. Note again the lack of correlation that we mentioned above, between the reaction to GC3 of motif preferences and that of occurrence departures from expectation.

### Strand asymmetries of occurrences and preferences as a function of GC content

**Figure 15:**
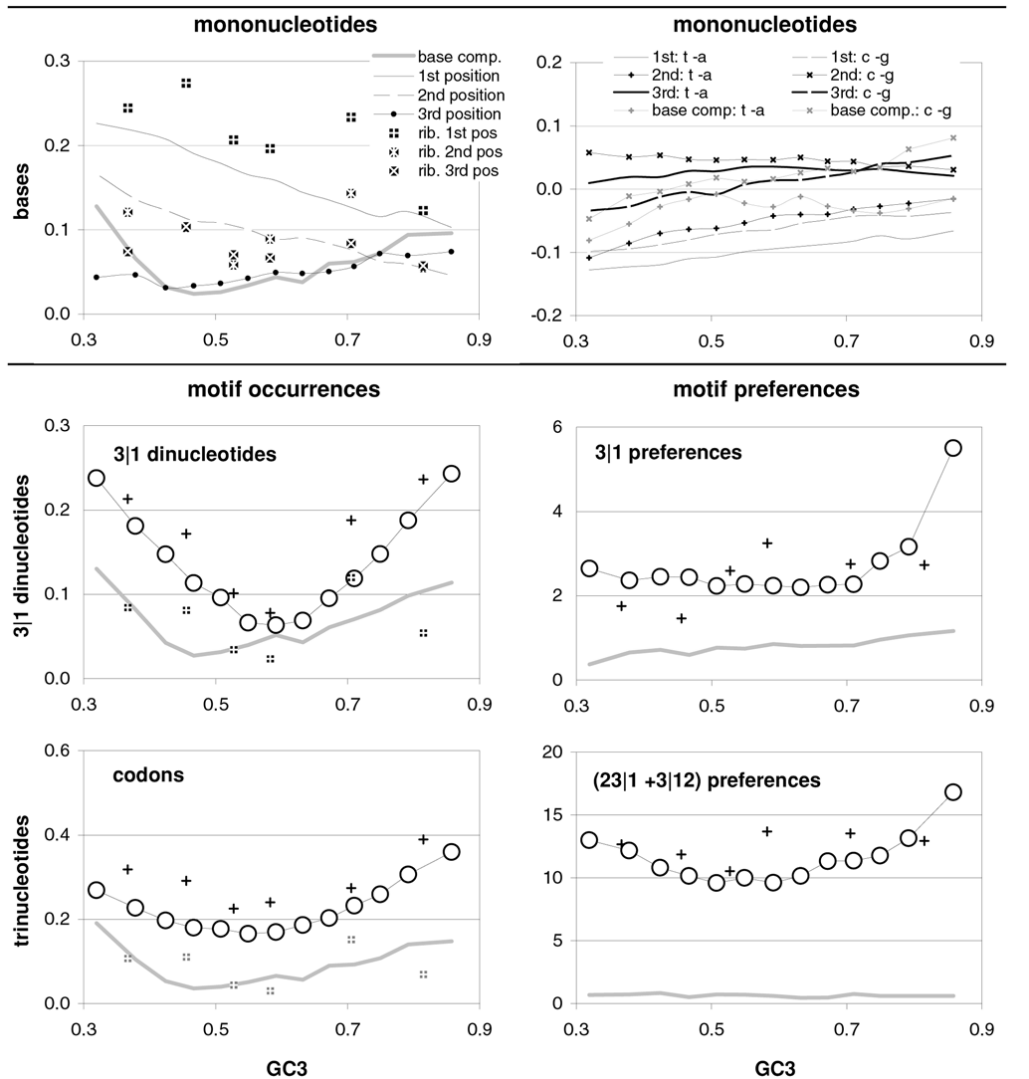
Transcribed-strand asymmetries as a function of GC3. The sum within each GC-sorted gene group of the absolute values of the differences (deltas) between values for complementary bases, 3|1 dinucleotides, and trinucleotides (top, middle, and bottom). At the top left are overall base-occurrence asymmetries at each codon position and on the right the TvsA and CvsG individual deltas. Also shown are base-composition predictions for all genes and asymmetries in 90 ribosomal-protein genes (subdivided into 6 groups, white signs, left plot only). In the middle and bottom are occurrence asymmetries and motif-preference asymmetries (left, right), for 3|1 dinucleotides and for codons or across-codon trinucleotides (see also Figure 16 and 17 for individual-motif deltas). Grey lines on the left are the base-composition expectations and on the right the “null” asymmetries of motif preferences from genes with previously randomized synonymous-codon locations. The +signs are for ribosomal-protein genes and the white +'s are the base-composition predictions.

Since transcription is well known to be mutagenic (see Discussion) and since transcription is a strand-specific process, we decided to present in Figure 15 the total strand asymmetries of coding-region base composition as well as those of the occurrences of and the preferences for dinucleotides and trinucleotides/codons. As index of total strand asymmetry we use the sum of the absolute value of the difference between the occurrences or preferences of complementary bases, dinucleotides, or trinucleotides (codons), over all such complementary pairs. Base-occurrence asymmetry decreases with GC3 and is strongest at the first codon position when GC3 is lowest. Note that these are GC-neutral base composition reactions to GC3 that are nonetheless quite marked for four of the six pairs of complementary bases at the three codon positions, with 3rd-position TvsA and 2nd-position CvsG being least reactive and 3^rd^-position TvsA showing also the least asymmetry. The occurrence asymmetries of 3|1 dinucleotides and codons peak at highest and lowest GC3 with a minimum at intermediate GC3. The departures from base-composition predictions are large and must be due to natural selection and/or NBDM. The asymmetries shown by the 90 ribosomal-protein genes are clearly noisier but appear to be higher overall, suggesting that we have to do with an effect due to transcription-associated mutation and/or repair, one which is weakest when GC3 is intermediate even when transcription is maximally high (as it is the case for ribosomal-protein genes). In the middle and the bottom left plots of Figure 15 we present the total asymmetries of the motif preferences of complementary across-codon dinucleotides or trinucleotides. These asymmetries are also structured, being lower at intermediate GC3 and higher especially at high GC3, albeit the patterns are not symmetrical, shifted as they are towards an inflection point at about 0.7 GC3. The preference asymmetries from the 90 ribosomal-protein genes are again much noisier and seem higher, although the dinucleotide asymmetries in the two groups of lowest GC3 are weaker than in non-ribosomal genes. The fourth point from the left appears to be an inflection point for some ribosomal asymmetries but more data is needed to corroborate this observation.

**Figure 16:**
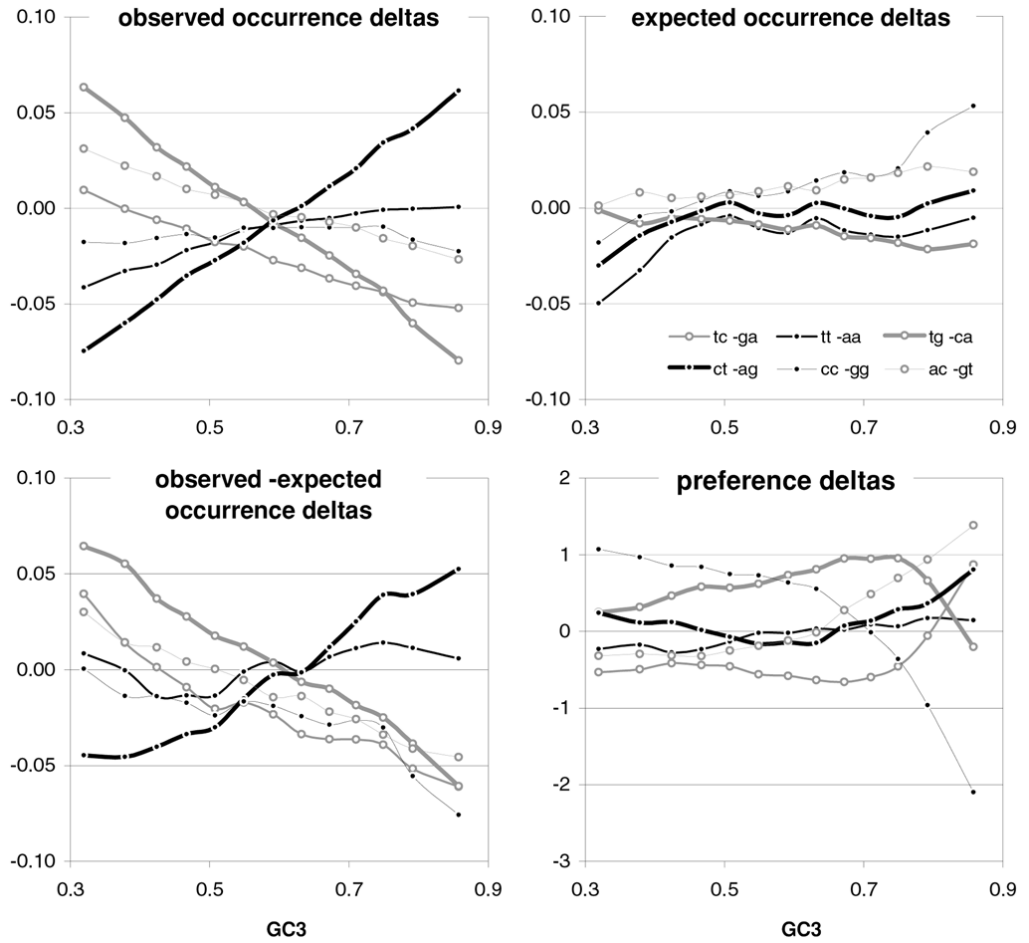
Transcribed-strand asymmetries of the occurrences and preferences of complementary 3|1 dinucleotides as a function of GC3. For each GC-sorted gene group, the asymmetries within each of the six pairs of non-identical complementary dinucleotides are expressed as the signed difference (delta) between the values of pair members. Clockwise from the top left: deltas for occurrence frequencies, for 1-composition expectations, for motif preferences, and for the difference between observed and expected occurrence deltas. In all plots pairs are labelled as in the upper right plot (where ct-ag hides tc-ga, however).

**Figure 17:**
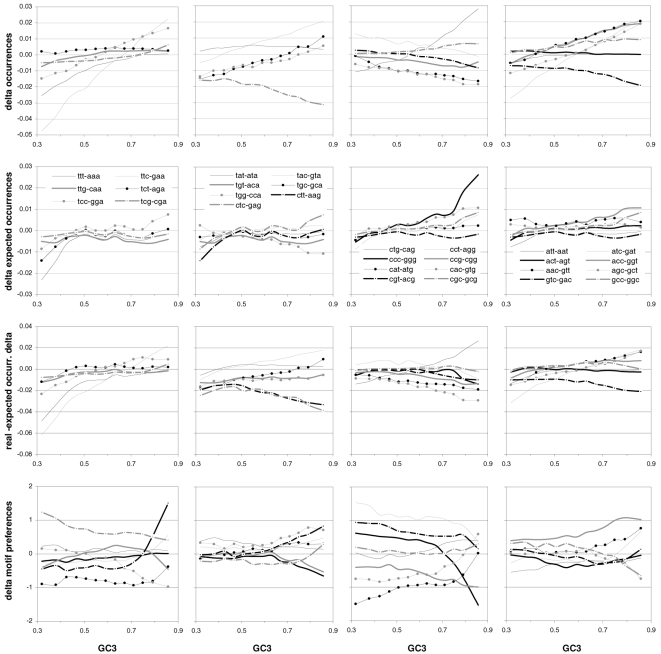
Transcribed-strand asymmetries of complementary trinucleotides as a function of GC3. From the top and for every GC-sorted gene group, the asymmetry of codon occurrences expressed as the signed difference (delta) between the frequencies of complementary codons, between expected occurrences (i.e., expected given the base composition that best fits codon occurrences), between observed and expected occurrence deltas, and between motif preferences. Occurrence asymmetries involving stop codons are not shown.

In Figure 16 we show the individual asymmetries of the occurrences of, and of the preferences for, complementary across-codon dinucleotides. In general, 3-position GorC (i.e., when the first base in a 3|1 dinucleotide is a G or a C) dominates the reaction of the asymmetries to changing GC3, but there are clear differences even when one “controls” for this variable (e.g., TGvsCA sinks more quickly with increasing GC3 than does ACvsGT). Note again the striking dissimilarities between preference asymmetries and the asymmetries of the difference between observed occurrences and base composition expectations. The trends shown by the asymmetries of individual pairs of complementary trinucleotides are even harder to interpret than those of dinucleotides but are included for completeness in Figure 17. In general, therefore, there is no obvious relationship between preference asymmetries and occurrence asymmetries or between preference asymmetries and those shown by the departures of the occurrences from base-composition expectations.

**Figure 18:**
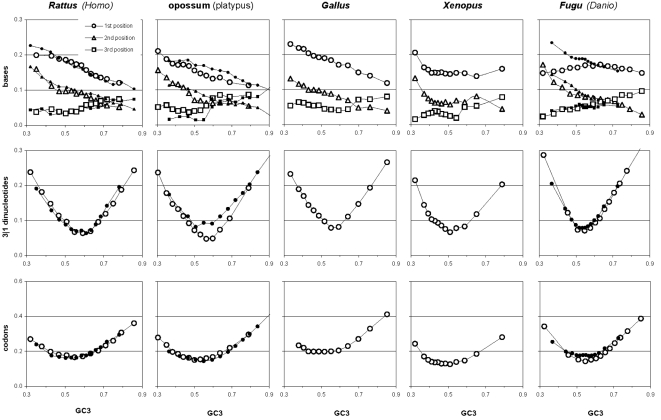
Strand asymmetries of the occurrences in vertebrate coding regions of mono-, di-, and tri-nucleotides as a function of GC3. Plotted is the sum within each GC-sorted gene group of the absolute value of the difference between the occurrence frequencies of each pair of complementary bases, dinucleotides, or trinucleotides (top to bottom). Solid symbols are for *Homo*, platypus, and *Danio*.

**Figure 19:**
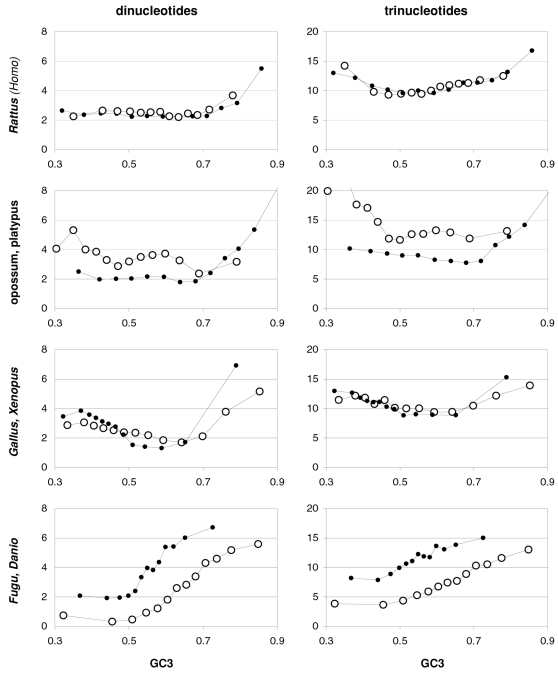
Transcribed-strand asymmetries in vertebrate genomes of motif preferences as a function of increasing GC content. The sum within each GC-sorted gene group of the absolute values of the differences between the motif preferences for complementary across-codon dinucleotides or trinucleotides (left, right). The solid dots are for *Homo*, platypus, *Xenopus*, and *Danio*.

In Figure 18 we show total occurrence asymmetries for the vertebrates considered so far. The general trends are like those shown by human genes if one excepts *Fugu*'s flat 1st-position base occurrence asymmetries which are always high (also found in *Canis*, not shown). A noteworthy trend is that the curves appear conserved across species despite spotty absences of certain GC3-defined groups. Trinucleotide inflection-minima lean generally towards lower GC3 so that the near symmetry of the human pattern is not typical. The comparative picture for motif-preference asymmetries in Figure 19 shows similar patterns for homeotherms and poikilotherms, the main between-group difference being the lower-GC3 inflection points of poikilotherms.

### Motif occurrences and preferences in intronic and non-genic DNA

**Figure 20:**
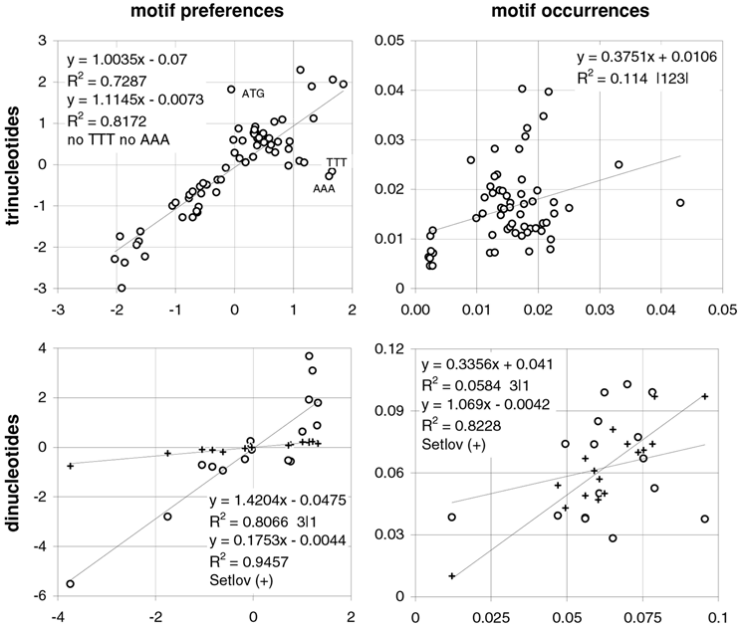
Preferences and occurrences in coding vs. intronic DNA. The correlation of motif preferences or occurrences (left, right) in the human coding-region dataset (vertical axis) with those in the human intronic DNA dataset (horizontal axis). Also shown are the correlations of intronic-DNA values with values obtained from the whole-genome dinucleotide data from human spleen cells tabulated in Setlov (1976; bottom plots, plus signs, vertical axis; see also methods and Figure 21) where Setlov's motif “preferences” are the chi values given the base composition implied by the dinucleotide occurrences.

**Figure 21:**
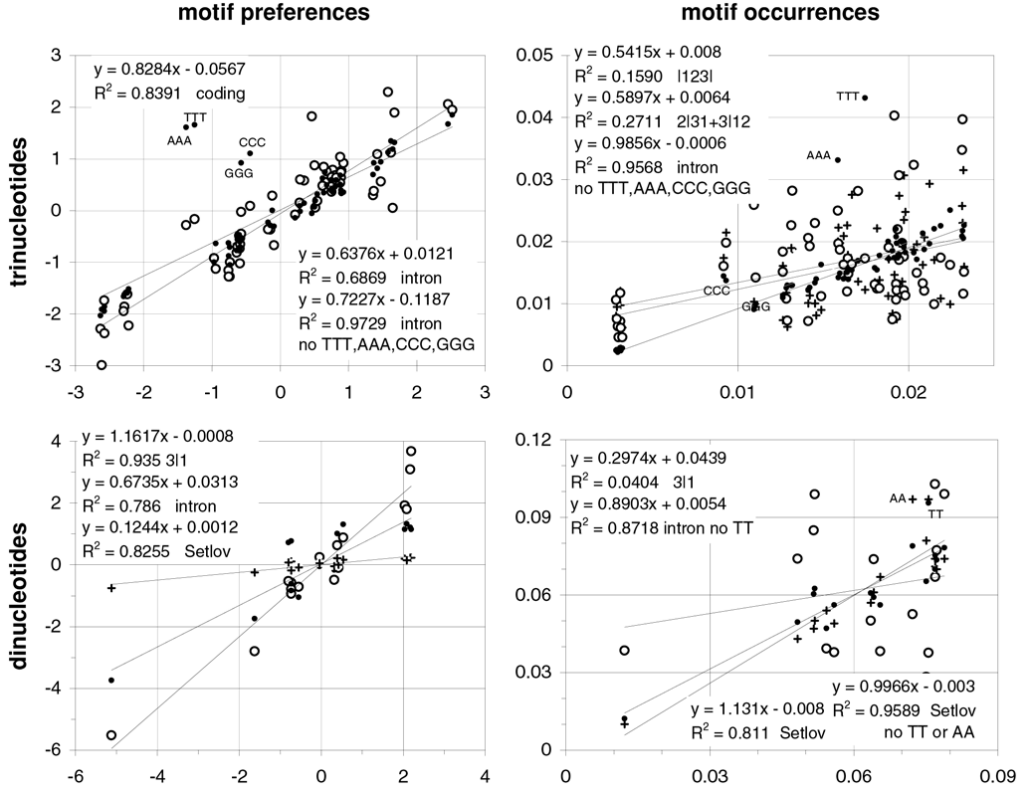
Preferences and occurrences in coding and intronic DNA vs. in non-genic DNA. The correlation between motif preferences and occurrences (left, right) in non-genic DNA (horizontal axis) vs. those in coding and intronic DNA (vertical axis, empty and solid circles). Pluses on the top right are for across-codon motif occurrences (23|1+3|12; vertical axis) and, in the bottom plots, for the dinucleotide occurrences in human spleen cells tabulated in Setlov (1976; left, vertical axis) and for the corresponding chi values derived from Setlov's dinucleotide occurrences (right, vertical axis). See also Methods and Figure 20.

Since primary-structural patterns in non-coding DNA are likely to be due to mutation pressure and genetic drift, we plot coding-region di- and tri-nucleotide occurrences and preferences against those in intronic and non-genic DNA (Figure 20 and Figure 21). Figure 20 shows that the preference R^2^s are much higher than the occurrence R^2^s ranging from 73 to 80%, indicating that similar forces structure intronic and coding-region motif preferences. Note also that both the dinucleotide occurrences tabulated by Setlov [8] and the motif preferences inferrable from these occurrences are highly correlated to the corresponding intronic values, indicating that these 1970s data provided already very accurate information about whole-genome motif preferences. In Figure 21 we plot coding-region and intronic dinucleotide and trinucleotide occurrences and preferences against those in non-genic DNA. Coding-vs.-nongenic occurrence R^2^s are low but preference R^2^s are quite high especially for dinucleotides (94% vs. 84%). Intronic-vs.-nongenic occurrence and preference R^2^s are high and become very high when one excludes outlayer base runs. For instance, the trinucleotide preference-preference R^2^ becomes 97% if one excludes TTT, AAA, CCC, and GGG, indicating that coding-region and intronic motif preferences are structured by the same forces underlying the motif preferences of non-genic DNA, i.e., of most of the genome. The dinucleotide occurrences and preferences in Setlov's data [8] are also very correlated to those in the non-genic DNA dataset, with the occurrence R^2^ reaching 96% when one excludes TT and AA. This is another clear indication that these 1970s data contained very useful information on whole-genome motif preferences.

### Occurrence-preference relationships in non-genic and intronic DNA as a function of GC content

**Figure 22:**
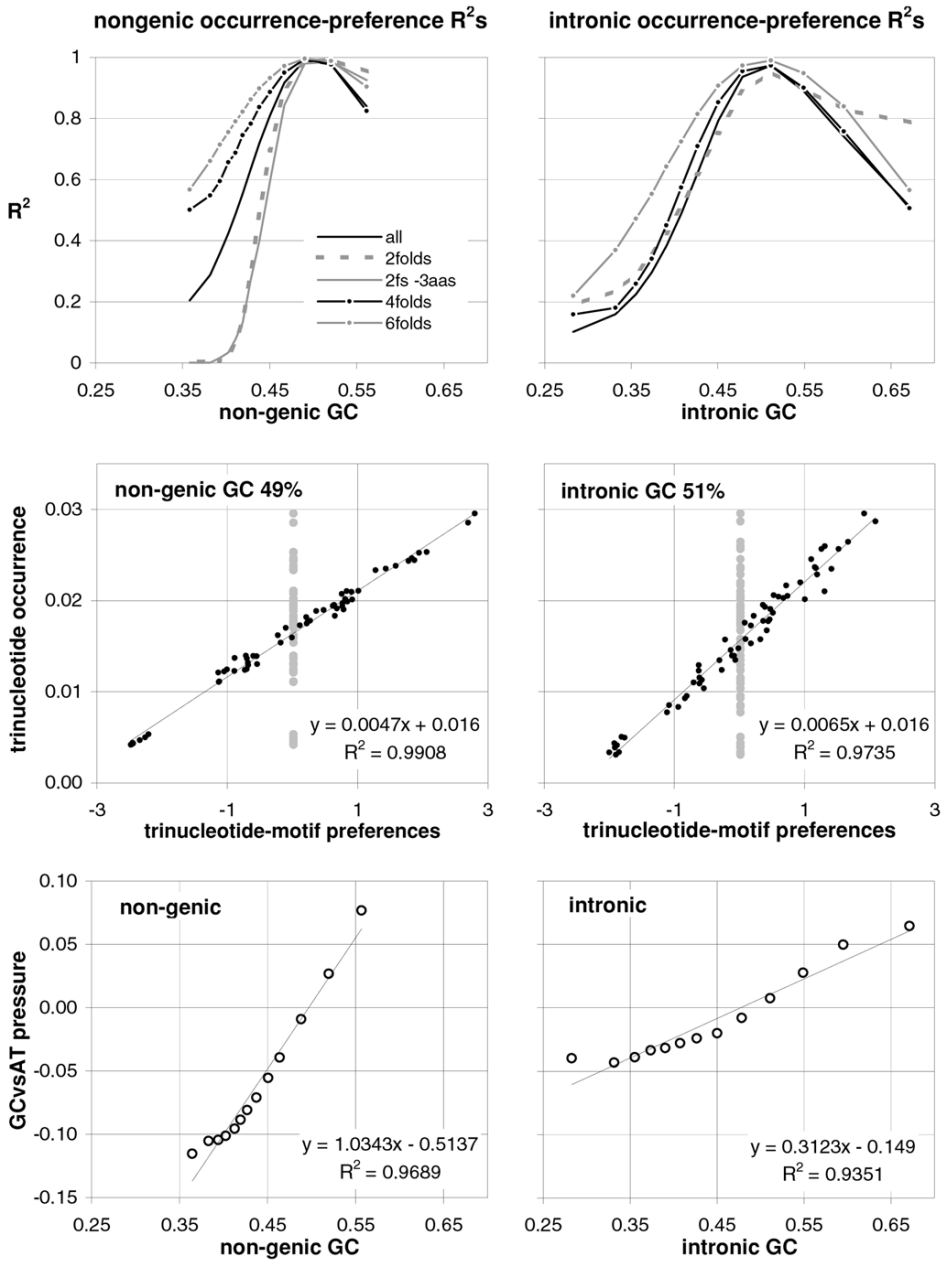
Trinucleotide preference-occurrence correlations in non-genic and intronic DNA vs. GC content. About 4,700 human nongenic DNA sequences and 54,000 human introns, sorted by increasing GC content and subdivided into 13 groups of equal size. On the top are the occurrence-preference R^2^s within each GC-defined group of nongenic and intron sequences. In the middle are plots of trinucleotide occurrence against motif preferences for the GC groups that gave highest occurrence-preference R^2^s (49% non-genic GC and 51% intronic GC). At the bottom are the correlations between the GC content and the difference between the sums of the preferences for trinucleotides containing CorG and TorA (see M&Ms).

The plots in Figure 22 show the GC-content reaction of trinucleotide occurrence-preference R^2^s in intronic and non-genic DNA. Peak occurrence-preference R^2^s of 97%+ are observed when GC is about 0.5 in intronic and non-genic DNA. These R^2^ values, however, can be quite low in either type of non-coding DNA, demonstrating again that occurrence-preference R^2^s do not need to be always high. The plots in the middle for 0.5 GC show that every one of the 64 trinucleotides falls on the regression line without exception, and that the main differences between the reactions to GC of non-genic and intronic occurrence-preference R^2^s are that the nongenic values for 4folds and 6folds sink more slowly with lower GC than the intronic ones and that the 2fold and 2f-3aas non-genic values sink much faster than the intronic ones. These differences make it remarkable that the non-genic and intronic all-motifs trends be nonetheless very similar (e.g., both have an ∼0.2 R^2^ at 0.3 GC; see also simulation results below). The bottom plots show again very strong correlations between the actual GC content and the preference-based GCvsAT pressures for each of the two kinds of DNA sequences. Note that the excursion of the intronic GCvsAT pressure is less extreme than the GC123 pressure in Figure 6, ranging between −0.05 and +0.05 rather than between −0.1 and +0.1, while the GCvsAT pressure of non-genic DNA has a range similar to that of the GC123 pressure.

**Figure 23:**
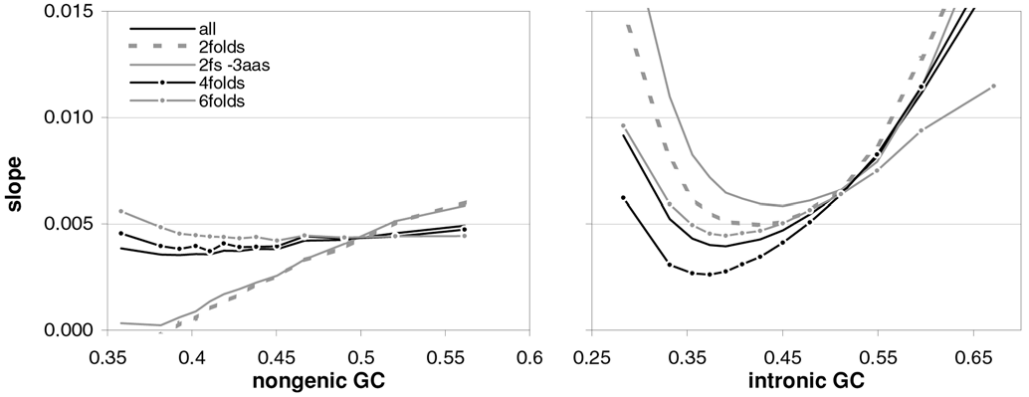
Slopes of the trinucleotide preference-occurrence correlations in non-genic and intronic DNA vs. GC content. From top to bottom: the slopes of the occurrence-preference correlation between trinucleotide-motif preferences and occurrences in groups of nongenic, intronic, and coding DNA of increasing GC (R^2^s are shown in the figure 22 and Figure 3).

**Figure 24:**
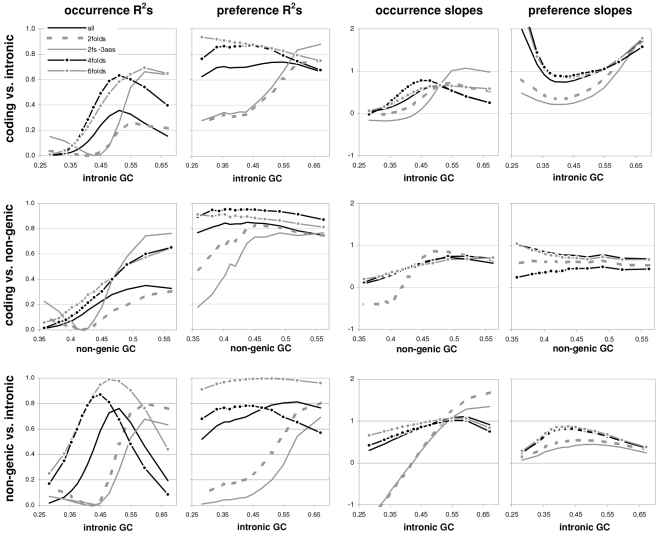
Trinucleotide preferences and occurrences in coding, intronic, and non-genic DNA. The right half of the figure shows from top to bottom the occurrence-occurrence R^2^s for trinucleotides from coding vs. intronic DNA, coding vs. non-genic DNA, and non-genic vs. intronic DNA, as a function of increasing GC content (horizontal axis); flanked to the left by the corresponding preference-preference R^2^s for off-frame trinucleotides. The figure's left half shows the corresponding slopes. For the top two rows we used human whole-genome coding-region values (and codons were used as “coding-region trinucleotides”); and for the bottom row we used values from the 49%-GC non-genic subgroup.

The reaction to GC of the occurrence-preference slopes in the non-coding data is shown in Figure 23. With a minor exception, the slopes are always positive and, surprisingly, the slopes most similar to those from coding regions are the non-genic ones, not the intronic ones. In Figure 24 we present the reaction to GC content of occurrence-occurrence and preference-preference R^2^s for coding vs. intronic, coding vs. nongenic, and non-genic vs. intronic DNA. In general, preference R^2^s reach much higher values than occurrence R2s, which is consistent with what was seen above, and the values are highest for the all-motifs as well as the 4- and 6-folds motif groups, and lowest for the two 2folds groups especially at low GC. Of interest is that when GC is intermediate the preference R^2^s of the various motif families tend to have quite similar high values, with those of 2fold motifs making the biggest upwards transition and those of 4folds and 6folds making slight downward adjustments. Also of possible interest is the “S” shaped reaction of the 2f-3aas occurrence R^2^s to higher GC content, that these R^2^s rise earlier in the coding-vs.-non-genic case, that 6fold R^2^s show a similar but much weaker reaction, and that the horizontally paired patterns of occurrences and preferences are often qualitatively similar. One should, however, not forget that unlike codon occurrences and motif preferences estimated via SC randomization (and like 23|1 and 3|12 trinucleotide occurrences and preferences), average motif preferences estimated from multiple intronic or nongenic sequences should be strongly correlated with overall motif occurrences when the preferences are clearly non-random (i.e., whenever an average preference is clearly different from 0.00).

Hence forces irreducible to base-composition effects govern the primary structure of non-genic and intronic DNA and these forces are very similar to those shaping coding regions. However, also here we want to stress, as we did before for coding-region DNA, that the very high preference-occurrence R2s observed when non-genic and intronic GC content is intermediate do not imply that the forces generating motif preferences do not shape non-genic and intronic DNA whose GC content is clearly higher or lower than 0.5. Indeed the correlation observed in both non-genic and intronic DNA between GC content and GCvsAT pressures estimated from motif preferences indicates clearly that this is not the case.

### Simulated relationship between motif preferences and occurrences

As mentioned in the Materials and Methods, we used 64×4 substitution matrices estimated from substitutions retrieved from genic- and nongenic-DNA alignments previously grouped by GC content, to generate non-genic and coding sequences in which every site was hit at least ten times. From these sequences we estimated motif preferences and occurrences and ascertained whether these quantities relate to each other as they do in real sequences. The 64×4 matrices derived from human-chimp/baboon non-genic DNA were each based on at least 18,000 substitutions but since substitutions of complementary bases were pooled, these matrices are equivalent to full-structure 64×4 ones estimated on the basis of about 36,000 substitutions. Of the 64×4 matrices derived from *Homo*-chimp/macaque intronic DNA, matrices 1 to 3 (from low to high GC) were each based on at least 144,000 substitutions and matrices 4 to 11 on at least 360,000 substitutions, with number 8 being based on 2,9 million substitutions from alignments whose GC ranged from 0.39 to 0.51 (each such GC group gave about 360,000 substitutions or more but the estimated 64×4 matrices generated equilibrium GC of about 0.40±0.005, so that the substitutions were pooled to estimate a single matrix). And matrices 12 to 17 were based on about 248, 206, 141, 80, 60, and 60 thousand substitutions, respectively. The no-strand-effect matrices derived from the same intronic substitutions are equivalent to full-structure 64×4 ones estimated from at least 120,000 substitutions.

**Figure 25:**
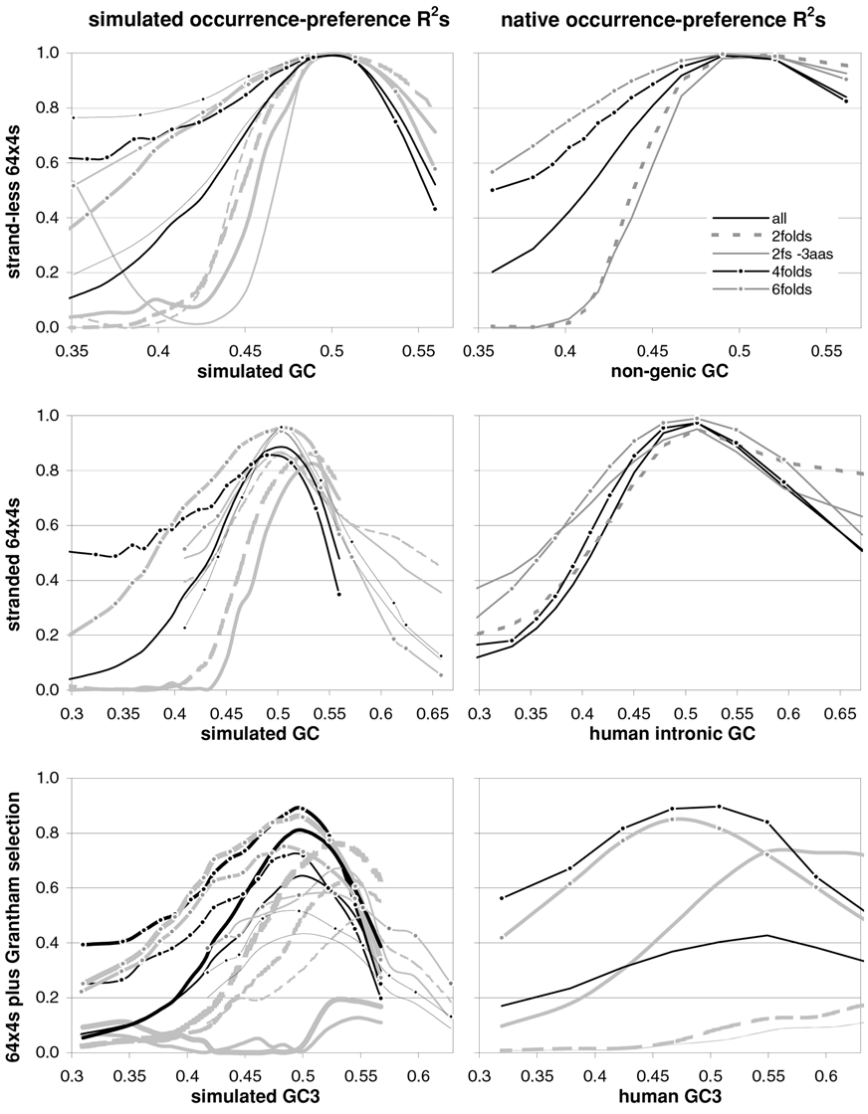
Simulated occurrence-preference R^2^s as a function of GC content. On the left are the occurrence-preference R^2^s (vertical axis) for trinucleotides in groups of 1000 simulated sequences whose every site was hit at least ten times with 64×4 matrices estimated from *Homo*-chimp/macaque intron alignments of increasing GC, plotted against the GC of the simulated sequences (horizontal axis). At the top left are results with 64×4s lacking strand effects (i.e., complementary substitutions were pooled to estimate rates) in absence of selection, flanked by the human non-genic pattern. In the middle on the left are results with 64×4s with full strand effects and no selection, flanked by the pattern of human introns. At the bottom left are results with Grantham non-synonymous selection (see M&Ms) and 64×4s with and without strand effects (thicker and thickest lines), flanked by the pattern of human coding DNA. Additionally, in thinnest lines on the left, are results with 64×4s from human-chimp/baboon non-genic DNA (top; highest GC: 0.48) or with 64×4s from mouse-rat/*Homo* coding-DNA alignments (middle and bottom; bottom is vs. GC123 which had wider GC excursion). The intronic 64×4 matrix that generated the two no-selection GCs of 0.408 and the GC3 of 0.42 was estimated on the basis of 2.9 million substitutions to human or chimp.

Figure 25 shows that in sequences generated by the above 64×4 matrices, motif preferences and occurrences relate to each other in very much the same way as in native sequences. The most striking matches are with the non-genic native pattern and, less so, with the intronic native pattern, and are delivered by the intron-derived 64×4s when their strand effects are erased (through pooling of complementary events) and by the 64×4s from non-genic DNA. These similarities include i) the peaking at about 100% R^2^ of every occurrence-preference R^2^s when GC is about 0.5; ii) higher R^2^s for the two 2fold groups and the 6folds when GC is above 0.5 than for the 4fold and the all-motifs group; iii) higher R^2^s at GC below 0.50 for the all-motifs, 4fold, and 6fold groups than for the two 2fold groups; iv) identical ranking of the R^2^s of 6folds, 4folds, and all-motifs below 0.5 (but for the no-strand-effect intronic 64×4s this is only so when GC is between 0.43 and 0.5); v) identical ranking above 0.5 GC of the R^2^s of 2folds, 2f-3aaas, 6folds, 4folds, and all-motifs.

**Figure 26:**
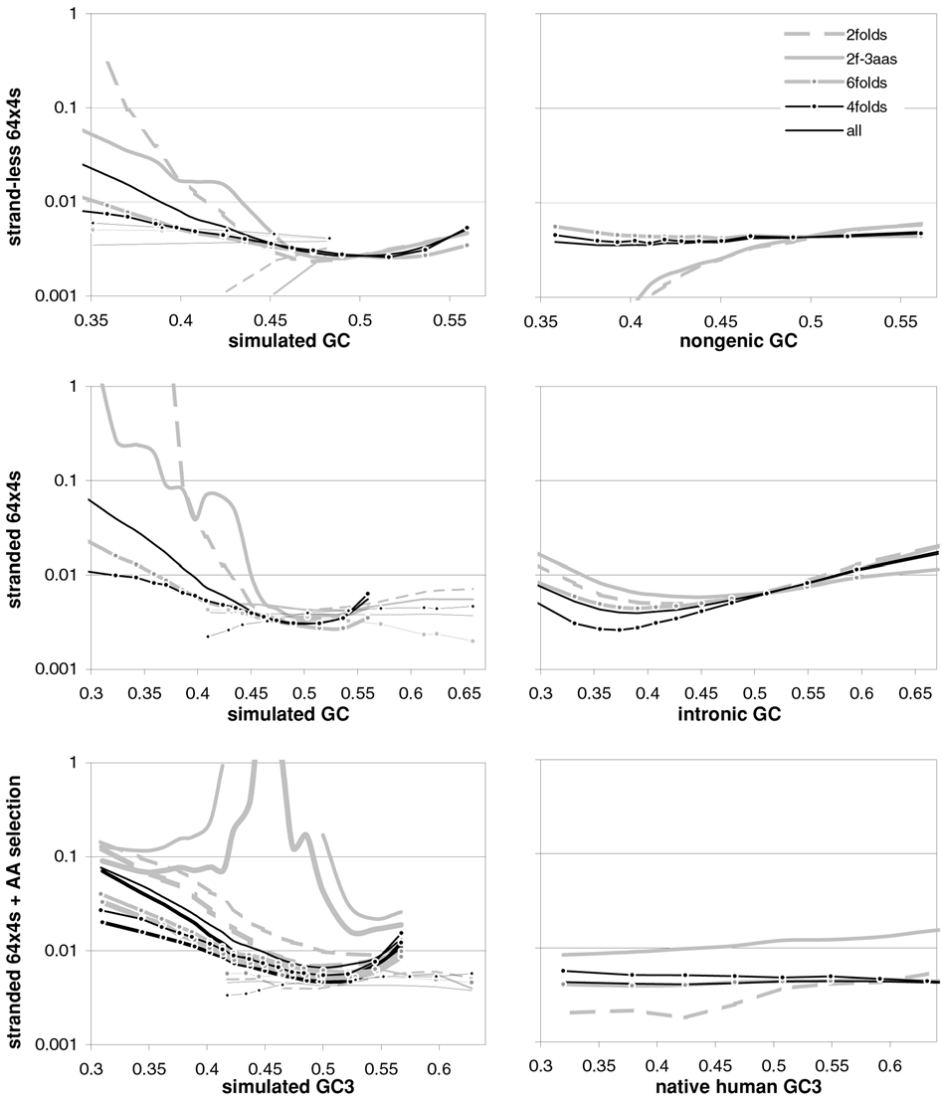
Simulated occurrence-preference slopes as a function of GC content. On the vertical axis is the slope of the correlation between average trinucleotide preferences and total trinucleotide occurrences as a function of increasing GC content (horizontal axis). On the right are the native patterns and on the left are those from simulated data. The data sets are the same as in the previous figure and are labelled identically. In plots with log vertical axis, the missing stretches of curves are due to negative slopes.

Remarkable similarity was also observed between the native intronic pattern and that generated by the intron-derived 64×4s with full strand effects and by the 64×4s estimated from mouse-rat/human coding-region alignments. These include i) higher R^2^s below 0.5 GC for 6folds, 4folds and all-motifs than for the two 2fold groups; ii) higher R^2^s below 0.5 GC for the 6fold and the two 2fold groups than for the 4fold and all-motifs groups; and iii) lower maximum R^2^s for the two 2folds than for the other three groups. However, the shift towards higher GC of the peak R^2^s of the two 2folds, which is observed neither in the native pattern nor in the pattern generated by the no-strand-effect intronic 64×4s nor in the pattern generated by the matrices derived from substitutions to mouse or rat, indicates i) that the strand effects in these 64×4s are typical neither for the long-term mutation regime that generated the native intronic patterns nor for the mouse/rat coding-region substitutions used to estimate the other set of 64×4s. This indicates that the strand effects inferrable from substitutions in human and chimp introns are not typical for the long-term mutational regime that has shaped intron primary structure. However, it is also thinkable that crucial NBDM effects are missing which if estimated accurately would allow say a 1024×4 matrix with full strand effects to reproduce the native intronic pattern (which would make the remarkable fit obtained with the no-strand-effect intronic 64×4s into an (un)felicitous coincidence). The patterning of the simulated slopes is shown in Figure 26 is much less similar to the native pattern than that of the simulated R^2^s, albeit the slopes obtained from mouse/rat coding-region 64×4s do show some agreement with the native slopes. On a related note, it is very likely that the simulated occurrence-preference R^2^s at 0.5 GC are lower than the native ones because –as it was already mentioned in the Materials and Methods– the 64×4 matrices were estimated using a mix of substitutions generated by possibly heterogeneous 64×4 regimes (since the alignments were sorted by GC content rather than by the unknown true rates of the 64×4 regimes that generated the harvested substitutions). This makes the at times striking similarities between simulated and native patterns presented above even more remarkable, as it does those presented below.

The partial recreation of the native intronic occurrence-preference patterns in sequences at equilibrium under 64×4 matrices estimated from coding-region substitutions is also important because it indicates that these coding-region matrices have not been biased by coding-region selection to the point of being unable to recreate the native intronic patterns. Indeed it is hard to imagine that such a biasing could leave unaltered the capability of the matrices to recreate the intronic occurrence-preference relationship (whose mutational underpinnings are themselves very unlikely to have been shaped by selection of a nature that would mimic a selection regime typical for coding regions). Moreover, for such selection against non-synonymous mutations to bias the 64×4 matrices used above, the selection regime would have to be extreme since most of the coding-region substitutions used to estimate the 64×4 matrices were 3^rd^-position substitutions anyway. Thus these results indicate strongly that the pattern of neighbor-base-dependence shown by coding-region substitutions is similar to that of intronic ones when the 64×4 matrices inferred from either type of substitutions generate similar GC content, and they also indicate that these two substitutional regimes reflect the pattern of NBDM that generated over the long term the primary structure of the chromosomal regions that happen to contain the majority of genes (i.e., regions whose GC content falls between 0.4 and 0.6, see Figure 9).

The occurrence-preference patterns from coding regions generated by 64×4 or 4×4 matrices when non-synonymous changes are rejected or accepted stochastically according to the Grantham distance between the original and the new amino acid (bottom plots in Figures 25 and 26; see Materials and Methods), are most similar to the native ones when the strand effects of the generating 64×4s are erased by pooling complementary substitutions (as it was the case when simulating intronic DNA). Among the similarities between native and simulated coding-region occurrence-preference correlations are i) the clear lowering of some R^2^s relative to simulated non-coding ones, ii) the higher R^2^s for 4folds and 6folds than for the other three motif groups, and iii) the shift towards lower GC of the peak R^2^s of 4folds and 6folds. These similarities between simulated and native coding-region patterns are remarkable not only because higher-order NBDM effects could not be simulated although we know them to be substantial, but especially because of the almost certain crudity of the Granthamian amino-acid selection regime that was imposed. The corresponding occurrence-preference slopes in the bottom plots of Figure 26 are ∼0.01 when GC is between 0.43 and 0.55, and are therefore congruent with the native slopes. However, like the slopes generated under no selection, the ones under selection are higher at low GC than at intermediate GC, unlike native coding-region slopes.

As alluded to above, the equilibrium GC content of the alignments from which the intronic 64×4 matrices were estimated is often much higher than the equilibrium GC that the matrices generate. This makes even more remarkable the match discussed above between the preference-occurrence correlations of groups of simulated and native sequences of similar GC content. Moreover, it also indicates that 64×4 syndromes of NBDM shift along chromosomal regions as more or less concerted modules of mutational effects and that therefore also the genome-metabolic features underlying NBDM heterogeneity may shift their location of influence as stereotypical syndromes of concerted factors. This is consistent with the correlations known to exist among the mechanistic factors that we suspect to be causally involved in determining NBDM heterogeneity (see Discussion), and it bodes well for the identification in the near future of the rules governing how 64×4 regimes change along genomic regions in reaction to changes in relevant features of DNA metabolism.

### Simulated relationship between preference-derived GCvsAT pressures and GC content

**Figure 27:**
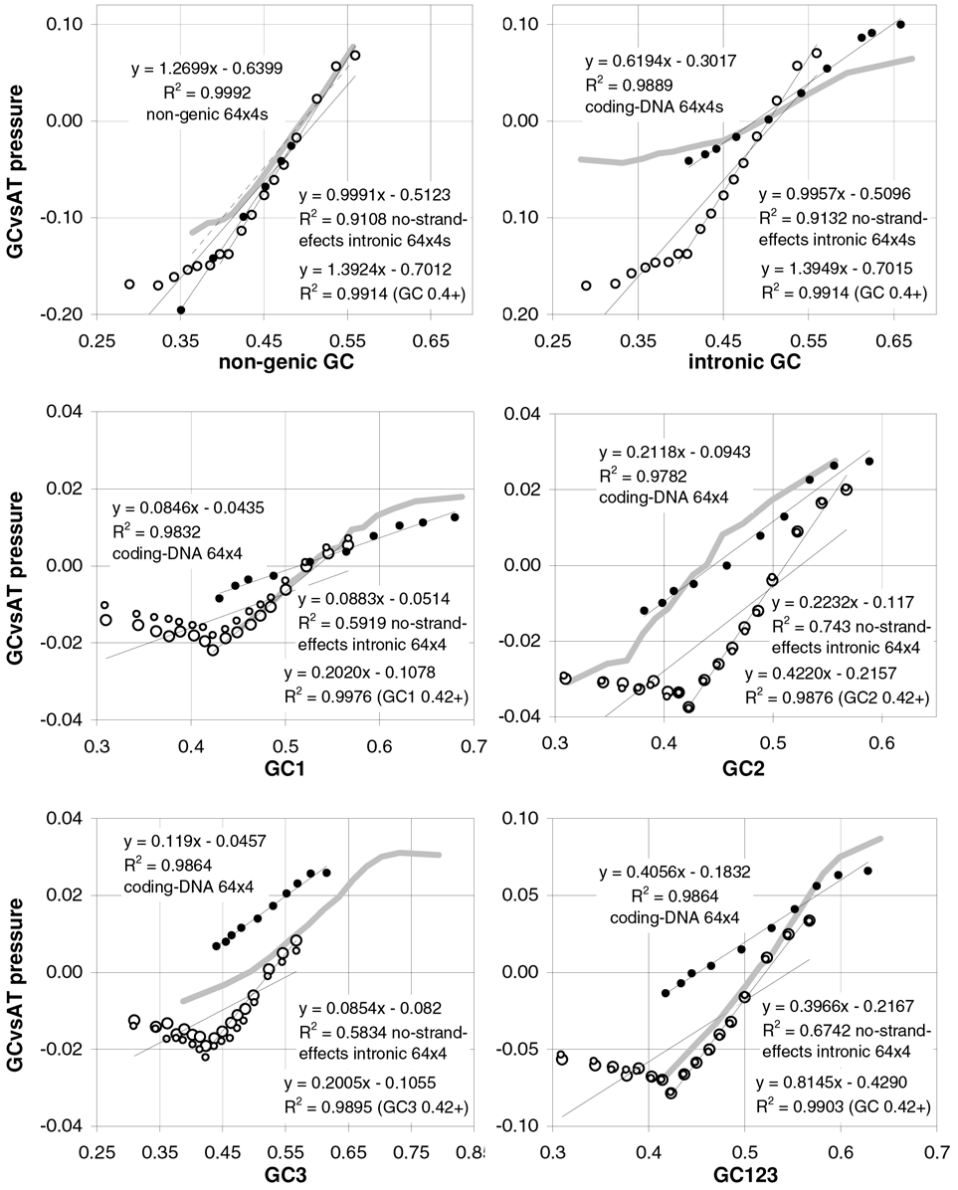
Simulated relationship between GC content and GCvsAT pressures derived from trinucleotide-motif preferences. The patterns were obtained from sequences generated by 64×4 substitution matrices derived from non-genic, coding, and intronic DNA alignments (solid, solid, and empty circles). The top plots show the pattern generated in absence of selection by the 64×4s derived from non-genic and coding-region DNA (left, right, solid circles), and by intronic 64×4s with and without strand effects (left, right, empty circles), the thick grey lines being the native non-genic and intronic human patterns (left, right). The middle and bottom plots show the patterns generated under Grantham non-synonymous selection, where grey lines are the mouse native patterns and smaller and larger empty circles label the results obtained from matrices with and without strand effects, respectively. The simulated data are otherwise the same as in the previous figure (see also Methods).

From the trinucleotide motif preferences measured in the aforementioned simulated sequences we estimated GCvsAT pressures as it was done above using native sequences. In Figure 27 these pressures are plotted against the simulated sequences' GC content, with the relevant native patterns being used as background. The plots show that in sequences simulated under no selection the R^2^ between preference-derived GCvsAT pressures and GC content is always above 98% if one uses generating matrices from non-genic and coding-DNA or the subset of the intron-derived matrices which generate GC contents above 0.41, where again the inclusion or not of strand effects in the NBDM matrices does not affect the patterns. Below 0.41 GC for the coding-region sequences simulated using intron derived matrices, the pressure-vs.-GC slope changes sign (while, perhaps connected to this, the occurrence-preference R^2^ for 2fold sinks strongly; note also that amino-acid selection lowers strongly the occurrence-preference R^2^ of 2f-3aas above 0.45 GC123). This abrupt change of the pressure-vs.-GC slope is not as marked under no selection and almost not noticeable in the native non-genic and intronic patterns. The change in slope therefore appears to be exacerbated by Granthamian amino-acid selection and is not found in native coding regions. This indicates that the native long-term regime of amino-acid selection may be different to some extent from the Granthamian regime at least as the latter was implemented here, and/or that the intron-derived 64×4s generating a GC123 between 0.3 and 0.44 are somewhat different from those that shaped over the long-term the motif-derived GCvsAT pressure and/or the GC content of mammalian coding-regions having similar GC123. Thus, if one puts aside these low-GC exceptions, the empirically derived 64×4 matrices generate motif preferences whose GC-weighted sums correlate almost perfectly with the equilibrium GC content they generate, and can even recreate some of the more subtle details of the native correlations, indicating strongly that the native correlations between GCvsAT pressure and GC content documented further above are in fact due to changes in NBDM. Moreover, the simulated patterns overlap to a great extent with the native ones indicating that the main drivers of the pressure-vs.-GC correlations in native sequences are mutational effects describable at the 64×4 resolution level. The simulated non-genic pattern is in this respect most striking but also the patterns under Granthamian amino-acid selection overlap remarkably well with the native ones (if one excludes the low-GC results from intron-derived 64×4s) and this despite that, as stated above, the regime of non-synonymous selection that we used was not chosen to deliver a close fit to the native patterns.

As a final digression, note that i) the GC contents generated by the 64×4s inferred from non-genic *Homo*-chimp/baboon substitutions and by the 64×4s inferred from *Homo*-chimp/macaque intronic substitutions are generally much lower than those of the source alignments (−0.10 units lower or more; not shown), and that ii) this is the case neither for the 64×4s inferred from mouse-rat/*Homo* coding-region alignments nor for their matched non-genic 4×4s (the intron-derived 4×4s the same GC as do their matched 64×4s). Antezana [21] showed that the non-genic 4×4 matrices that are also used here deliver an equilibrium GC that fits almost perfectly the actual GC of the source alignments, a fit that decreases slightly when the 4×4 rates are modified to correct for the possible bias caused by predicted multiple hits at ancestral CG dinucleotides to which Duret [22] ascribed the fit (although it is highly unlikely and thus debatable that local mutation rates from CG to TGorCA, i.e., of CG methylation, do not change over evolutionary time; see Monroe *et al* [23] and citations therein; and Holmquist and Ashley [20]. Indeed the modified rates deliver an extraordinarily high R^2^ of 99.6% but lower the equilibrium GC slightly by about 0.045 (the mean human-chimp CG occurrence was taken as ancestral and the CtoT and GtoA rates at CGs were set 10× higher than the basal rate as per Duret's pers. comm.; work not shown).

Of direct interest here is that the GCs generated by the plain- and the adjusted-rate non-genic 4×4s are not nearly as low as those generated by their matched 64×4s, indicating that the richness of NBDM effects allowed by the 64×4 framework can at times not suffice for an accurate recreation of the equilibrium GC content. Indeed, 4×4 matrices estimated from equilibrium data must recreate equilibrium GC even when the mutation process is highly neighbor-base-dependent, but this is not guaranteed for NBDM matrices that fail to accommodate essential wider-context features of the actual mutation process. Additionally, the fact that the *Homo*-chimp/macaque intronic 4×4s (and 64×4s), with or without strand effects, generate much lower GC content than those derived from human-chimp/baboon non-genic alignments indicates that in humans and chimp the genomic regions with many genes or close to gene-rich regions may be going through a phase in which mutation rates are quite different from those that shaped over a much longer time the regions' primary structure at least with respect to GC content. This is fully congruent with recent demonstrations that somatic transcription patterns including those of non-scheduled “background” transcription, and thus the patterns of chromatin compaction, “evolve” very rapidly, which for the germline is likely to be even more the case. The likely occurrence of such transient non-equilibrium situations should not prompt conclusions about long-term directional trends, however, since the landscapes of chromatin compaction and of scheduled and non-scheduled (“background”) transcription in the germline, even if only distant resonances of constraints on somatic performance, must oscillate within a functionally tolerable range (which may, however, shift with major metabolic or Bauplan remodellings). Importantly, such oscillations could easily affect the whole genome when caused epiphenomenally by changes in high-level switches of the landscape of somatic chromatin compaction [20]. Molecular evolutionists may have to start estimating “effective substitutional regimes” in the spirit of the multiple-generation “effective population size” of population genetics [24]. Estimating rates from *Homo*-chimp/outgroup coding-DNA alignments could provide additional insight and so would estimating non-genic and genic 64×4 and 4×4 matrices from a variety of internal and terminal branches of the mammalian phylogeny.

### What governs the primary structure of vertebrate DNA?

**Figure 28:**
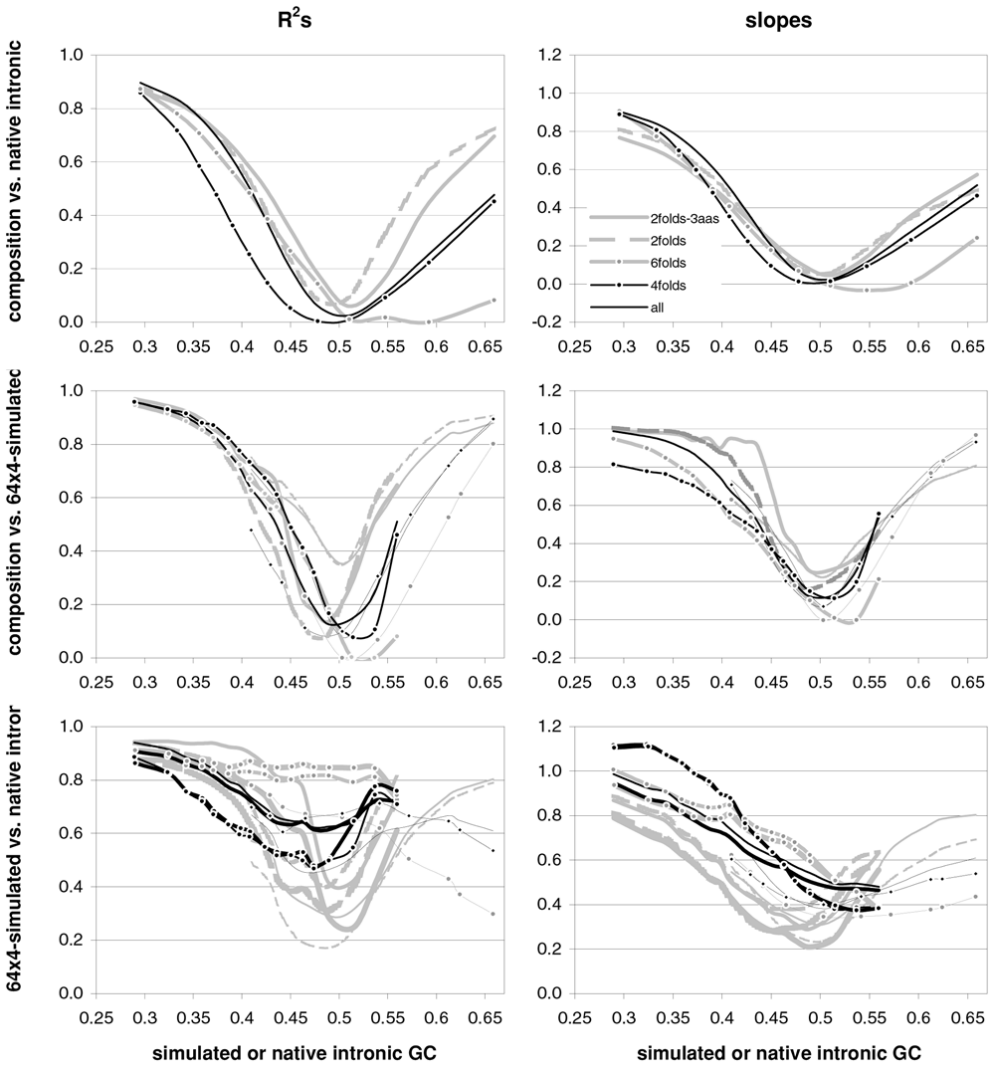
Trinucleotide occurrences in native and simulated intronic DNA vs. base-composition expectations, as a function of GC content. R^2^s and slopes (left, right; vertical axis) of the correlations between native or simulated intronic trinucleotide occurrences (top, middle) and their base-composition expectations, as a function of increasing GC content (horizontal axis; see also Methods). The simulated occurrences come from sequences generated by the intron- and coding-region-derived 64×4 matrices used for the previous figures (thicker and thinner lines, respectively). At the bottom are the R^2^s and slopes between native intronic values and the ones generated by 64×4 matrices. Thickest lines in the bottom plots indicate results with intronic 64×4s lacking strand effects.

The native and simulated patterns presented above indicate clearly that NBDM shapes the primary structure of non-genic, intronic, and coding DNA. However, one could argue that the primary-structural foundations of the patterns and relationships shown so far are only subtle departures from what one would expect under a regime of context-independent mutation that delivers the base composition. This is not the case. In Figure 28 we show, for increasing GC content, the R^2^s and slopes of the correlation between trinucleotide occurrences in native intronic DNA and those in random sequences whose base composition is identical to that of the various GC-sorted groups of native intronic sequences. The R^2^s range from quite high (at most 90%) at low and high GCs, to 0% at intermediate GCs, i.e., they are lowest exactly when the native non-genic and intronic preference-occurrence R^2^s reach extraordinarily high 95%+ values (Fig. 22 and 25). Figure 28 also shows that trinucleotide occurrences in simulated DNA at equilibrium under the 64×4 matrices estimated from mouse-rat/*Homo* coding-DNA and under 64×4s derived from *Homo*-chimp/macaque intronic DNA, relate to the corresponding base-composition expectations in a very similar way as do native intronic occurrences. Finally, the figure shows that the native and simulated occurrences are strongly correlated with each other, especially the all-motifs, 4fold, and 6fold groups. The slopes, moreover, range between 1.0 and 2.0 indicating that the primary-structural features due to 64×4 NBDM are of the same order of magnitude as those observed in native intron DNA. Therefore NBDM-simulated and native intronic patterns are correlated because both sets of occurrences are very similarly structured, while the occurrences expected given the base composition differ strongly from the native occurrences, especially when GC content is intermediate.

**Figure 29:**
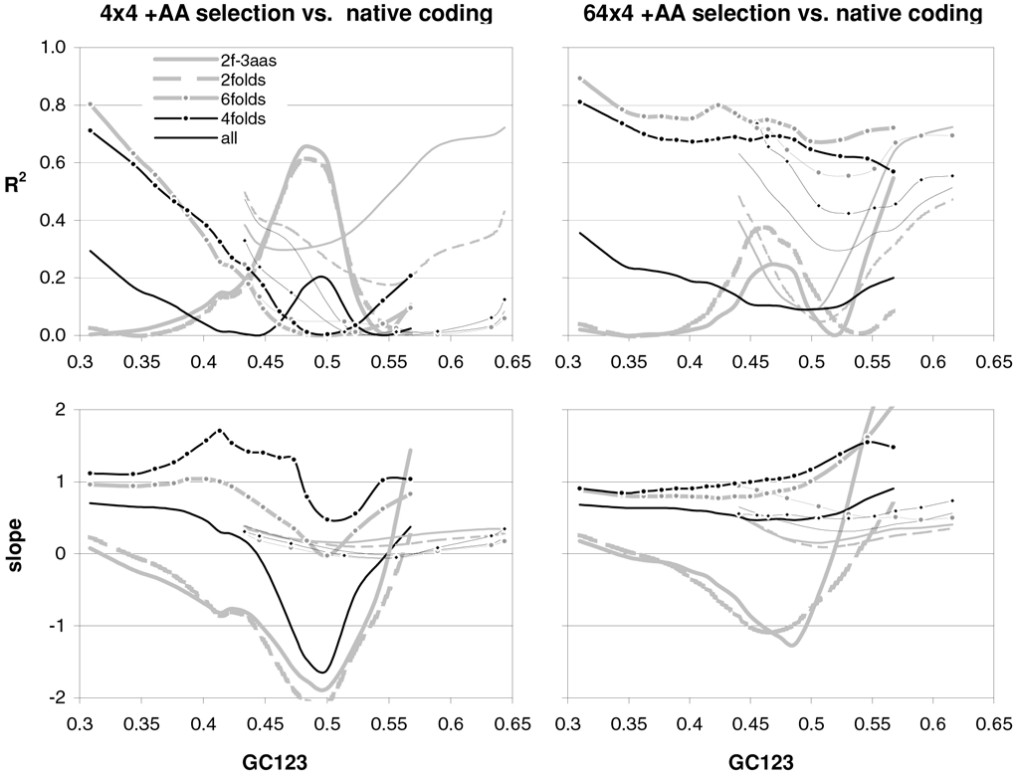
Codon occurrences in native vs. simulated coding DNA, as a function of GC content. The R^2^s and slopes (top, bottom; vertical axis) of the correlations between codon occurrences in native human genes and in simulated coding DNA generated by 4×4 or 64×4 matrices (left, right) with erased strand effects estimated from human-chimp/macaque intron alignments under Grantham selection of non-synonymous changes, as a function of increasing GC total (horizontal axis). Thinner-line patterns are for mouse genes and full-strand-effects 64×4s derived from mouse-rat/human coding-DNA alignments (leaning right). The fit with intronic 64×4s having full strand effects was worse.

The situation for coding regions is shown in Figure 29 which presents, for a range of GC contents, the R^2^s and slopes of the correlation of the codon occurrences in native human CDSs with the occurrences in DNA simulated under Granthamian selection of non-synonymous changes generated by 4×4 and 64×4 matrices from mouse-rat/*Homo* coding-region alignments or from *Homo*-chimp/macaque intron alignments with erased strand effects. Note, incidentally, that i) expectations derived by assuming a single base composition per GC group, delivered very poor fit since a single base composition is not very suitable to fit the effects of amino-acid selection, and that ii) allowing a different base composition at each of the three codon positions delivers better fit but is biologically implausible. Therefore we do not show either set of results. The figure shows that the 4fold and 6fold codon occurrences generated under amino-acid selection by intronic 64×4 matrices correlate with the native occurrences more strongly than do those generated by 4×4 matrices, except at low GC content where they do it similarly. Furthermore, the slopes for 4fold and 6fold codons generated by 64×4s under selection fall remarkably consistently close to 1.0 while those generated by 4×4s tend to be closer to 0.0 (but 4×4-generated 4fold slopes are close to 1.0 at low GC). The 2fold and 2f-3aas occurrences generated by 4×4 or 64×4s intronic matrices correlate weakly and negatively with the native ones (but those from 4×4s do it more strongly, but negatively, between 0.45 and 0.5 GC). The 64×4s derived from coding-DNA substitutions generate only positive slopes, an improved fit for the two 2folds, and a slightly worse fit for 4- and 6-folds relative to the intronic 64×4s; while the corresponding 4×4s deliver much worse fit, except for the slightly better fit of the two 2folds (with near-zero slopes). Therefore in presence of Grantham selection on non-synonymous changes, the empirically estimated 64×4 matrices generate more realistic codon occurrences than do the 4×4 matrices.

**Figure 30:**
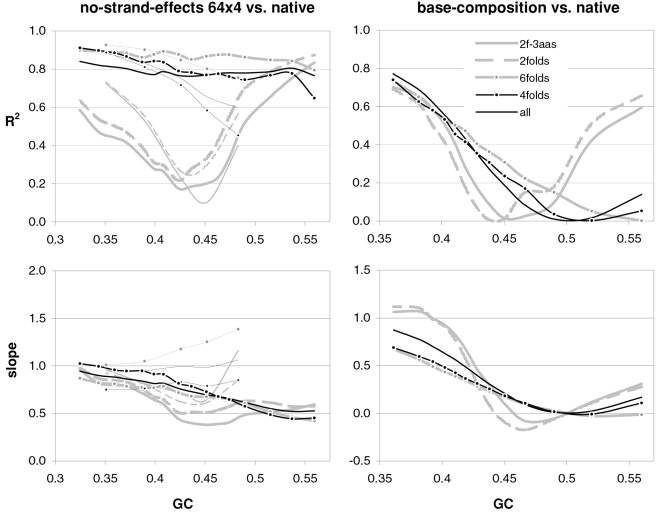
Trinucleotide occurrences in native vs. simulated non-genic DNA as a function of GC content. The R^2^s and slopes (top, bottom; vertical axis) of the correlations between trinucleotide occurrences in human non-genic DNA and the corresponding occurrences in simulated DNA (left) generated by no-strand-effects 64×4 matrices estimated from human-chimp/macaque intron-DNA alignments, as a function of increasing GC total (horizontal axis). The thinner-line patterns were generated by no-strand-effects 64×4 matrices from human-chimp/baboon non-genic DNA alignments. On the left are results with occurrences derived from the base composition that best fits the human non-genic occurrences vs. the native occurrences (the fit with the corresponding 4×4 matrices was slightly worse).

**Figure 31:**
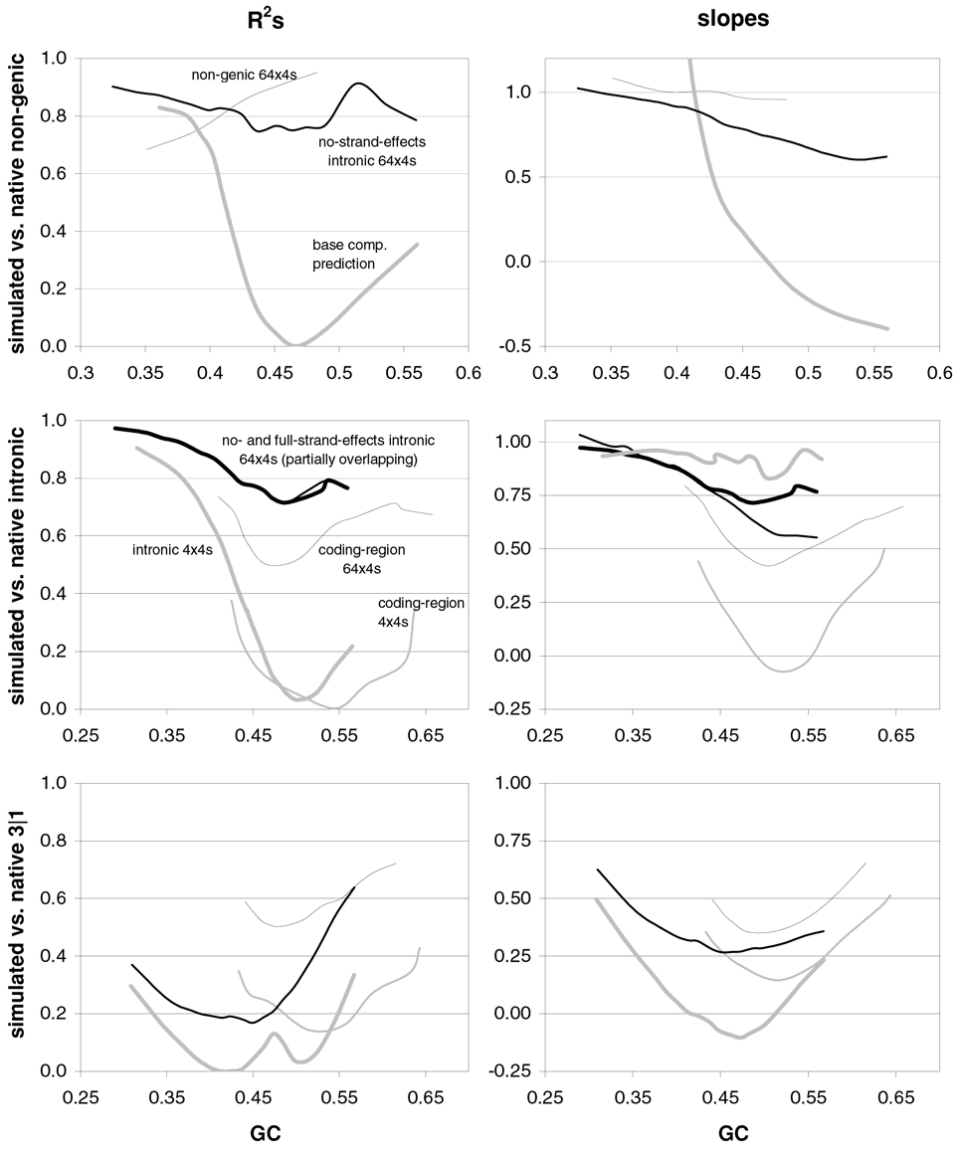
Dinucleotide occurrences in native vs. simulated non-genic, intronic, and coding DNA as a function of GC content. The R^2^s and slopes (left, right, vertical axis) of the correlations of dinucleotide occurrences in native non-genic, intronic, and coding DNA (top to bottom) vs. those in simulated DNA generated by 4×4 (grey lines) or 64×4 matrices (black lines) estimated from non-genic, intronic, and coding-region substitutions, as a function of increasing GC total or GC123 (horizontal axis; but the grey-line results in the top two plots were obtained using base-composition predictions rather than simulated occurrences). Thick lines are results with intron-derived matrices and thinner lines in the top plots are results with non-genic-DNA matrices and –in the middle and bottom plots– results with coding-DNA matrices. The intron-derived 64×4s used for the top and bottom plots had no strand effects and so were those for the middle-plot results highlighted with the thickest black line.

Figure 30 shows how simulated non-genic occurrences correlate with native ones for increasing non-genic GC content. Here again deliver the 64×4 matrices (with erased strand effects) a much closer fit to the native non-genic occurrences for the 4fold, 6fold, and the all-motifs groups than would a well-fitted 4×4 matrix. The fits for the two 2fold groups was best when using the 64×4s derived from non-genic DNA. The slopes are quite close to 1.0 but decrease towards 0.5 with increasing GC. Finally, Figure 31 shows how simulated dinucleotide occurrences correlate with native ones. The fit is best between the native non-genic or intronic dinucleotide occurrences and those generated by the intronic and non-genic 64×4s. However, even when the fit to the 64×4-generated dinucleotide occurrences is not high –e.g., between native and simulated 3|1 dinucleotides in coding DNA– the fit delivered by 64×4 NBDM is nonetheless almost always better than that with 4×4 matrices or base-composition predictions.

### Simulated trinucleotide motif preferences

**Figure 32:**
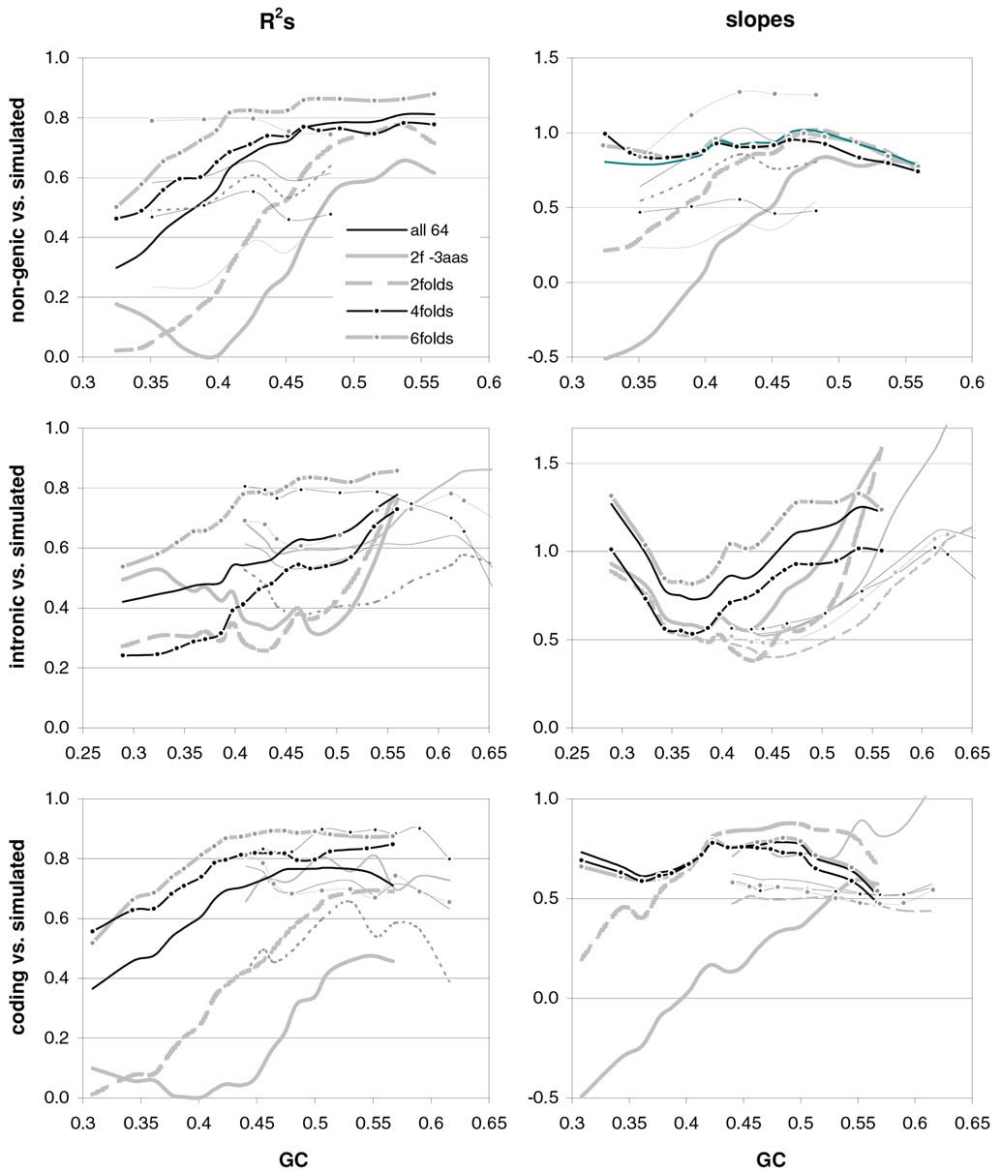
Trinucleotide-motif preferences in native vs. simulated DNA as a function of GC content. R^2^s and slopes (left, right; vertical axis) of the correlations of trinucleotide-motif preferences in native non-genic DNA, intronic DNA, and coding regions (top, middle, bottom) with motif preferences estimated from sequences generated by 64×4 matrices inferred from non-genic *Homo*-chimp/baboon substitutions (top; thin lines), from mouse-rat/*Homo* coding-region substitutions (middle, bottom; thin lines), and from no-strand-effect intronic matrices (top, thicker lines) and full-strand-effects intronic matrices (middle, bottom; thicker lines), as a function of increasing GC total or GC123 (horizontal axis). Simulations for the bottom plots included Grantham selection of generated non-synonymous changes.

**Figure 33:**
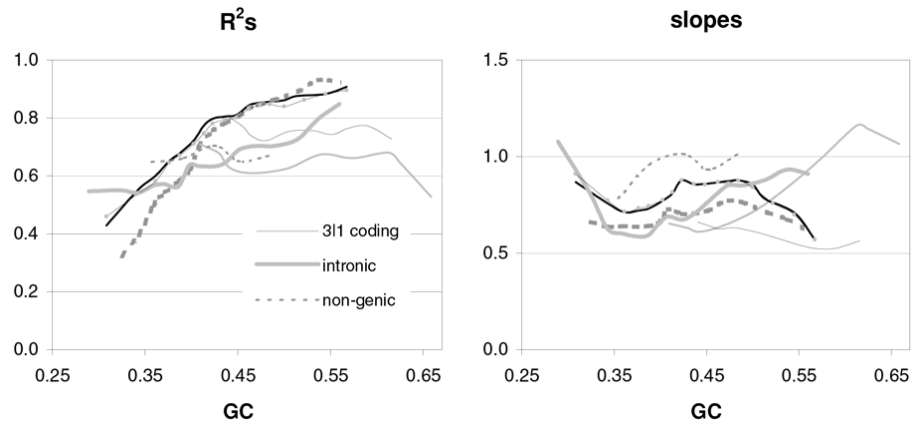
Dinucleotide preferences in native vs. simulated DNA as a function of GC content. The R^2^s and slopes (left, right, vertical axis) of the correlation of dinucleotide preferences in native coding, intronic, and non-genic DNA (black, grey, and segmented grey, respectively) vs. those in DNA simulated using 64×4 matrices estimated from non-genic, coding-region, and intronic substitutions (thin segmented line; solid grey and black thin lines; and all thicker lines, respectively, as a function of increasing GC total (horizontal axis; but the thin black dotted lines are for 3∥1 dinucleotides simulated using intronic 64×4s with erased strand effects; the fit to intronic and non-genic dinucleotides with intronic 64×4s lacking strand effects was almost identical as with full strand effects). Coding-region preferences for 3∥1 dinucleotides were estimated from sequences simulated under Granthamian amino-acid selection.

For the simulation work above we used 64×4 matrices estimated from real substitutions and their immediate one-site upstream and one-site downstream contexts. Native preferences for trinucleotide motifs, however, are certainly influenced by a wider context so we tried to ascertain to which extent the 64×4 resolution of our simulations can reproduce the various native motif preferences. In Figures 32 and 33 we show the correlation between preferences for trinucleotide and dinucleotide motifs estimated from native baboon non-genic DNA, human intronic DNA, and *Rattus* coding regions and the corresponding preferences generated by the 64×4 matrices that were used also above. The figures show that the preferences estimated from simulated intronic and coding-region DNA are highly correlated to the native preferences, with the R^2^s ranging from 60 to 90%, the slopes falling in the proximity of 1.0; and the only exception being the two 2folds groups which tend to have lower R^2^s and slopes more distant from 1.0. The preferences from simulated non-genic DNA are those most cleanly correlated to the native ones but the two 2folds groups are not fit very well here either. These high correlations and slopes close to 1.0 indicate that the 64×4 matrices estimated from coding-region substitutions are capturing most of the neighbor-base-dependence of mutation in and close to genes. The fact that the fit is not complete, however, leaves room for substantial 1024×4 mutation effects (and wider) as well as for biasing effects due to selection that make the neighbor-base-dependence of coding-region substitutions not fully identical to that of mutation. On the other hand, in view of these results it is hard to entertain the possibility that the true pattern of mutation is one of a main 4×4 matrix with only one or very few neighbor-base-dependent effects. Note, finally, that erasing strand effects from the intronic 64×4s does not worsen the correlations (shown only for 3|1 dinucleotides), indicating again that the native preferences have been shaped by strand effects which alternate between the two strands over neutral-evolutionary time, at least in the germline.

### One-base non-synonymous mutations expected from empiric 64×4 and 4×4 mutation regimes

We evaluated and contrasted the mutability under 64×4 and 4×4 mutation of those coding regions in our largest human dataset whose GC123 matches the equilibrium GC123 generated under Granthamian amino-acid selection by the 64×4 and 4×4 mutation matrices derived from intronic and coding-region DNA (a GC content that is very similar for each pair of matched 64×4 and 4×4 matrices; see above). We simply summed the total mutation rate towards any base at every site in every qualifying coding region for each paired matrix, and then divided each paired sum by the total number of bases evaluated. This way we obtained a per-base mutability of the qualifying coding regions given each matrix. By dividing the 64×4 mutability by its 4×4 counterpart, we then obtained the “relative 64×4 mutability” of the qualifying coding regions, and expressed it as a percentage. We also calculated such relative mutabilities for 1^st^, 2^nd^, and 3^rd^ codon positions in each set of coding regions (and results with per-coding-region rather than per-base mutabilities were practically identical).

Additionally, to evaluate the potential deleteriousness of the differences in 64×4 vs. 4×4 mutability, we kept track of the subset of the 20×20 individual mutabilities away from each given amino acid towards another that are caused by single-base mutations, and did so for all codon positions together and for each position separately. This allowed us to multiply each of the 20×20 sums of amino-acid mutabilities by its corresponding amino-acid-to-amino-acid distance in the Grantham, the EX, or the Blosum100 matrices [17], [18], [19]. The sum of these 20×20 products under a 64×4 matrix was then divided by that under its matched 4×4 matrix to obtain the relative “deleteriousness” of the 64×4 matrix, given an amino-acid distance matrix and a set of qualifying coding regions.

**Figure 34:**
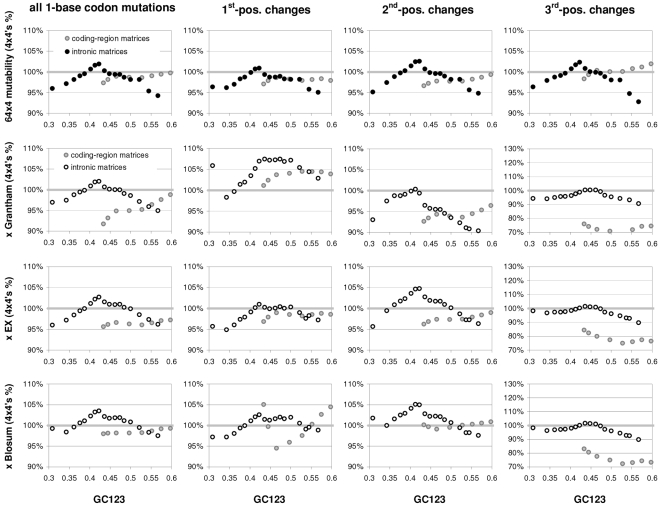
The mutability of human coding regions under empirically estimated 64×4 and 4×4 mutation. The top plots show the sum of the mutation rates at every base in all the human coding regions whose GC123 matches to ±0.01 the GC123 generated by the intronic or coding-region-derived 64×4 matrices used for the previous figures (bigger and smaller symbols), divided by the total number of bases evaluated and then by the corresponding value obtained with each matched 4×4 matrix. From left to right are the sums of the rates of every possible one-base mutation at every codon position and at first, second, and third positions separately. The bottom three rows of plots show the potential deleteriousness of the same 64×4 regimes evaluated according to either the Grantham, the EX, or the Blosum100 amino-acid-distance matrix (from top to bottom), again relative to the values under matched 4×4 mutation. The 20×20 replacement mutabilities were each multiplied by its corresponding value in the 20×20 distance matrix, the 20×20 products were summed, and this sum was then divided by the corresponding 4×4 sum (see Results).

The top four plots in Figure 34 show the relative 64×4 mutability of human coding regions over a range of GC contents. The total and the 1^st^-, 2^nd^-, and 3^rd^-position relative mutabilities are very similar to each other and fall mostly below 100%, the lowest values being ∼95% at both 0.3 and 0.57 GC. Values up to 103%, however, are found between about 0.4 and 0.45 GC, i.e., over the range in which intronic 64×4s do not recreate well the native patterns shown by several motif groups, especially those of the two 2folds groups (see Figure 25 and above). These results indicate that throughout their recent primary-structurally effective evolutionary history, most human coding regions are likely to have mutated less than they would under closely matched 4×4 regimes. However, it is a truism that whenever a mutation regime shapes the primary structure of a DNA sequence for a long time –and the results in the previous pages leave little doubt about this being the case for NBDM in vertebrates– then a different mutation regime is likely to generate more mutations on such DNA than the actual regime which structured the DNA over the long term. Moreover, observing the contrary in the case of the NBDM regimes estimated here would rather indicate that the regimes are not similar to those that generated the primary structure of the sequences used in the evaluation. Similarly, no straightforward prediction seems possible when the evaluated sequences have, e.g., a GC that is very different from the equilibrium GC that the evaluated mutation matrices generate. For instance, we have observed that the relative 64×4 mutabilities are mostly much higher than 100% when they are estimated using coding regions whose GC matches the actual GC of the alignments from which the substitutions used to estimate mutation matrices were retrieved, rather than the equilibrium GC generated by the estimated matrices. For instance, the mutabilities reach about 110% between 0.47 and 0.69 GC (not shown), indicating that in the recent past the 64×4 regimes inferrable from the lineages to *Homo* and *Pan* were seriously maladaptive compared to their matched –but counterfactual– 4×4 regimes, a result that will not be further discussed here. But truism or not truism, it appears very hard to dispute that the total mutability of sequences which have been shaped by a mutation regimen characterized by strong neighbor-base-dependent effects, should be lower under that regime than under a matched regime of 4×4 mutation.

The plots in the second to fourth rows from the top in Figure 34 show that the potential consequences for organismic fitness of differences in mutability under 64×4 vs. 4×4 mutation can be markedly mitigated or exacerbated by the likely highly heterogeneous average impact of the different amino-acid replacements. For instance, multiplying 1^st^-position non-synonymous mutabilities by the corresponding Grantham distances erases the plain-mutability “advantage” of NBDM at first positions even for coding-region-derived matrices (but neither of the other two distance matrices leads to the same result). Other two examples are the fact that the plain-mutability “disadvantage” of NBDM estimated at third positions for intronic matrices in the 0.40–0.45 GC range is erased by multiplying by any of the three distance matrices; and the fact that the mixed plain-mutability picture at 3^rd^-positions given coding-region-derived matrices becomes a deleterious-impact difference very favorable to NBDM after multiplying by any of the 20×20 matrices.

**Figure 35:**
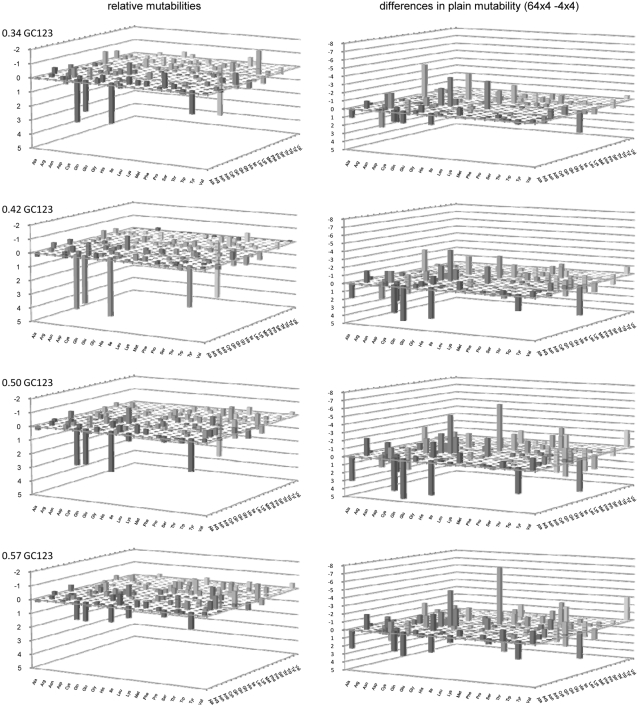
The expected incidence of amino-acid replacements due to one-base codon mutations in human coding regions, under 64×4 relative to 4×4 intronic mutation. Results from top to bottom are for 0.34, 0.42, 0.50, and 0.57 GC123 (i.e., points 2, 8, 14, and 17 from the left in Fig. 34). A positive value on the left indicates that the 64×4 mutability is (value +1.0)-fold higher than it 4×4 counterpart (1.0 was substracted to obtain 0.0 when two rates are identical); and a negative value indicates that the 4×4 value is abs(value −1.0)-fold higher than its 64×4 counterpart. On the right are the differences between each plain replacement mutability under 64×4 mutation and its 4×4 counterpart, to highlight the replacement dominating the patterns in Figure 34. Values on the right were rescaled to make the largest positive difference equal to 5.0 (i.e., the 245.7 Arg –>Gln rate). Therefore, values above the 0.0-plane, both left and right, indicate an advantage for NBDM (i.e., a 64×4 mutability lower than its 4×4 counterpart). Numeric values are shown in Figure 36.

**Figure 36:**
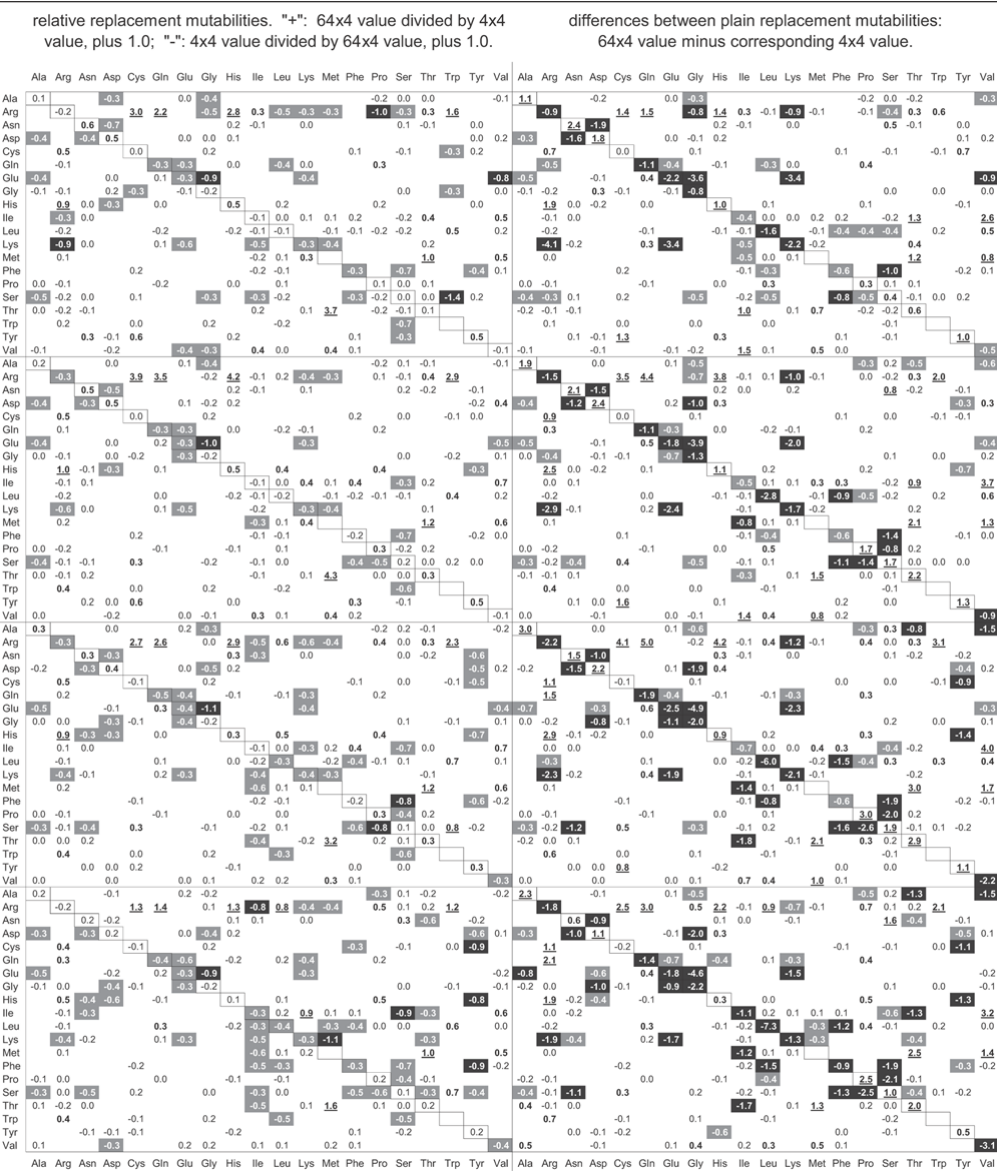
The expected incidence in human coding regions of the amino-acid replacements due to one-base point mutations under intronic 64×4 vs matched 4×4 mutation. Dark cells highlight low 64×4 values and light cells with boldface highlight high 64×4 values (details in Figure 35).

In Figure 35 we contrast the expected occurrence under 64×4 vs. 4×4 mutation of the individual amino-acid changes in the 20×20 matrix that are due to single base changes, for four of the 18 groups of human coding regions and pairs of matrices used to generate Figure 34. The values plotted in Figure 35 are shown in Figure 36 as numbers and the reader is advised to consult both. The four groups represent the GC content closest to 0.5 and those at which 64×4 NBDM delivers the largest overall plain mutability advantage relative to 4×4 mutation (0.55), the largest disadvantage (0.42), and 2^nd^-largest low-GC advantage (0.34). The trend both left and right is that under 4×4 mutation more of the 20×20 replacements would happen more frequently than under 64×4 mutation. In the upper left plot, e.g., 99 values are smaller than 0.0 and 69 are larger, and 67 are below −0.1 and 47 above 0.1; whereas in the top right plot the numbers are 99 to 69 (obviously, given the numbers at the left plot) and 71 to 48, respectively. This trend is also found in the lower plots albeit it is less marked in the second-row plots (from the top), consistent with these being the two plots for the largest 64×4 disadvantage, although the alternative explanation with few large effects determining overall mutability differences should not be forgotten. When the ±boundary is larger, the numbers above and below the boundaries decrease, the trend becoming erratic and switching polarity for the most extreme effects which of course involve the very mutable CG dinucleotide and are erased under 4×4 mutation. All in all, however, the left-side plots indicate that several replacements are moderately less likely under 64×4 mutation than under 4×4 mutation –instead of a few being markedly less likely– but the plots on the right show that several mutability differences favoring 64×4 mutation are nonetheless quite large. This makes it harder to point out important replacements that natural selection may be suppressing or tolerating to greatest extent than if it had been the case that both the left- and right-side plots showed the same performers delivering similarly high notes. We will not discuss further the relationship between the differences in expected replacement generation in Figures 35 and 36 and the underlying differences between individual 64×4 and 4×4 base-mutation rates. This would require examining 64×64 matrices, and would make more sense if one knew the actual 64×4 matrices that generated the native intronic patterns.

There is an aggregation of large positive and large negative values on, and close to, the diagonals (best seen in Figure 36). In particular, the diagonals on the right display a bigger number of large negative values than of similarly large positive ones and, consistent with this, the sums of diagonals are negative, except for the second matrix from the top (−2.3, 2.4, −3.7, and −10.8). This indicates that in human coding regions 64×4 mutation may generate fewer synonymous mutations than would 4×4 mutation regimes that generate the same GC content, a puzzling result unless evolution by natural selection has modified 64×4 mutation rates to optimize third-position base occupancy (which could be functionally important, e.g., for regulating the occurrence of trinucleotides that mutate towards undesired amino acids, or to balance the usage of the 2fold and 4fold subfamilies of each 6fold family). An example is the seven-unit lower mutability under 64×4 mutation of Leu-to-Leu synonymous changes in the bottom-right in Figure 36, which represents ∼10% of the sum of all values favoring 64×4 mutation at 0.55 GC (the percentages for the upper matrices being 3, 6, and 9, from the top). To the very least, the fact that the diagonals are rich in both large negative and large positive values indicates that in human coding regions NBDM generates many fewer and many more synonymous changes across SC families than are expected under matched 4×4 mutation. This disparate behavior does not bode well for molecular-evolutionary calculations that assume 4×4 mutation and “neutral” behavior of SC mutations, and it bodes even worse for calculations assuming that 4×4 mutation rates are the same in constrained and unconstrained DNA of the same or dissimilar GC content (see also Discussion).

Therefore, the major indications provided by Figures 34, 35, and 36 are that i) NBDM regimes similar to those that have shaped mammalian genomes, and the actual native coding-region primary structures which NBDM has contributed to generate, are such that very large increases in both plain and “deleterious” genome-wide mutability would ensue if these coding regions were to start mutating in a context-independent way that delivers the coding regions' GC content, and ii) that differences between the 20×20 amino-acid distances of the order of magnitude entertained in published amino-acid-distance matrices can mitigate or exacerbate strongly the deleterious consequences of such genome-wide mutability differences, because the individual replacement mutabilities under realistic NBDM can differ from their 4×4 counterparts in disparate ways.

## Discussion

We presented above several lines of evidence indicating that basic features of the primary structure of coding, intronic, and non-genic vertebrate DNA at and below the trinucleotide level are phylogenetically highly conserved –if not outright convergent– and appear to be strongly shaped by regimes of neighbor-base-dependent-mutation (NBDM) that must also be highly conserved and that change markedly across genomic regions, generating strongest primary-structural departures from base-composition expectations when GC content is intermediate as well as motif over-/under-representations that increase linearly with GC content. We also showed that 64×4 NBDM effects similar to those that generated over the long term these primary-structural features would generate in human coding regions both fewer mutations than would matched 4×4 regimes and a different spectrum of amino-acid-to-amino-acid mutations, two strong indications that the long-term pattern of vertebrate NBDM may reduce markedly the deleterious non-synonymous mutability relative to context-independent mutation.

The presented evidence leaves little doubt about the major role played by NBDM in shaping the primary structure of vertebrate genic and non-genic DNA at and below the trinucleotide level, making a strong case not only for the capability of such mutation of generating the conspicuous, richly structured, and phylogenetically highly conserved patterning of motif preferences in every major type of vertebrate DNA, but also for the possibility that NBDM has played a central role in the generation of commonly studied features of the mammalian genome's primary structure like GC content and codon occurrences, features that are also highly conserved, even if less so than motif preferences, and are normally accounted-for by invoking an interplay of context-independent mutation effects and constraints related to protein encoding and gene expression. Similarly, the fact that motif preferences are very similar even in mammals and *Gallus* is solid evidence that the empirical pattern of mammalian NBDM has been conserved by natural selection for a long time. (Indeed shuffling the synonymous codons showing 3^rd^-position differences in our mouse-rat/human coding-region alignments suffices to blunt markedly the motif preferences in each aligned sequence; not shown).

It is unclear what the maximal extent is to which NBDM effects can be tuned to minimize the non-synonymous mutation load, and therefore we cannot evaluate the extent to which the vertebrate pattern of NBDM may have been so tuned. And it is also unclear whether a strong minimization of this load, let alone weaker ones, would deliver genome-wide decreases in the non-synonymous mutation load that would actually translate into performance and progeny-quality advantages large enough for selection to detect. This notwithstanding, it would be very surprising if the highly conserved regimes of NBDM that have shaped vertebrate primary structure over the long term will turn out to be neutral or even outrightly deleterious relative to the hypothetical 4×4 ones.

Indeed, the differences in mutability shown in Figures 34, 35, and 36 under 64×4 vs. 4×4 mutation, together with these differences' potential impact on the total deleteriousness of generated non-synonymous mutations shown in Figure 34, indicate that the vertebrate pattern of NBDM has been tuned through evolution by natural selection and has been conserved by natural selection for a long time afterwards, because it lowers the non-synonymous genetic load. These results show indeed that NBDM regimes similar to those that have actually shaped vertebrate genomes are sufficiently structured to bias strongly the quality and the quantity of the non-synonymous mutations which these regimes should generate in mammalian coding regions relative to their 4×4 counterparts, making it very likely that throughout the history of vertebrates the actual regimes of vertebrate NBDM have had a major impact on whole-genome deleterious point-mutation rates and thus on the fitness of vertebrate organisms. This in turn makes it very unlikely that the marked NBDM effects which characterize mutation and substitution patterns in vertebrates may not only have resisted being optimized by natural selection for such a long time –or may have managed to remain invisible to it– but may have also been conserved as rigidly as they have been, merely because they are obligate pleiotropic effects of other functions under direct natural selection.

Furthermore and importantly, we will see below that from a purely molecular-biological perspective, the tuning of NBDM may not be difficult to generate mechanistically and is not likely to entail problematic trade offs with the optimization of other functions. Therefore, it is reasonable to assume that mutants modifying the pattern of NBDM should arise readily and that they should be strongly selected-for when both i) they lower sufficiently the mutation rate from specific motifs to others and ii) the suppressed motif change(s) are those leading to particularly undesirable amino-acid replacements, at least in the most important coding regions of the genome (or to motifs that impair the mRNAs of these genes [25].

The pattern of NBDM has the potential to alter on a genome-wide scale not only the deleteriousness of germline mutation but also, and perhaps even more importantly, that of somatic mutation, and therefore it could impact fitness by affecting not only the genetic quality of one's progeny but also, and perhaps mainly, individual viability. In principle, NBDM should affect every gene in the genome and every cell in the soma, giving favorably patterned NBDM effects extraordinary adaptive resonance, unlike “normal” favorable mutations that are expected to affect individual genes, circumscribed phenotypic cascades, and specific tissues. However, if somatic mutability is truly what has been tuned evolutionarily, then NBDM would be optimized not necessarily over all genes in the genome but rather mainly over those genes that are crucial to somatic performance, perhaps during specific life-history stages or in specific tissues. Under this scenario the overall impact on individual fitness of deleterious mutations would be minimized but the shaping by NBDM of the primary structure of many genes would be epiphenomenal and these genes' deleterious mutability under NBDM could appear much less optimized, if at all, were it to be assessed in isolation (see below).

As intriguing, plausible, and likely as the non-synonymous genetic-load advantage of NBDM may appear, solid evidence will have to be gathered to substantiate its existence. Crucial for this effort will be, in our opinion, that one estimate with reasonable accuracy the 20×20 amino-acid replaceabilities at least in mammals, a feat that we believe only sophisticated retrospective molecular-evolution work is likely to accomplish in the near future. Indeed, experimental methods to measure directly the fitness consequences of the 20×20 amino-acid replacements –in the style of Yampolsky and Stoltzfus [18] but over tens of thousands of proteins and on the basis of hundreds of thousands of amino-acid replacements assayed in a realistic mammalian system that would allow one to evaluate individual fitness rather than mere protein-function correlates– do not appear technologically imminent. And even if such an experimental system were to become available, it is hard to imagine how it could be used to quantify effects of amino-acid replacements that affect fitness by altering in subtle ways the costs entailed by the amino acids needs of the expressed proteome [26], [27], [28] rather than the structure and function of individual proteins.

Estimating amino-acid replaceabilities using molecular-evolutionary approaches will require solving the applied-mathematical challenges that NBDM poses for traditional molecular-evolution calculations, and that appropriate and sufficient data be gathered for one to estimate NBDM rates accurately. Neighbor-base-dependent mutation can indeed be crippling for retrospective and extrapolative molecular-evolution work. Hua Tang and MAA, e.g., have shown (unpublished results) that under a regime of NBDM typical for mammalian introns, Tang's method for estimating amino-acid replaceabilities from diverged coding regions [29], [30] delivers a mere 38% R^2^ between the Grantham acceptabilities used to generate a set of simulated sequences and the acceptabilities the method estimates from the same sequences, an R^2^ that rises only nominally to 42% if one excludes from the analysis every codon and dicodon with one or more CG dinucleotides (whereas the R^2^ and the slope are perfect 100% and 1.0 under context-independent mutation). Furthermore and no less problematic, other preliminary work by MAA shows that i) a phylogeny test whose type I error is correct under context-independent mutation [31] under-estimates *P* values by about 50 to 150% under NBDM if a context-independent evolutionary model is used to calculate crucial likelihoods; and ii) that multiple hits can be underestimated strongly under the same conditions, especially between very diverged sequences. Equally important, as we showed above, NBDM can generate marked primary-structural departures from base-composition expectations that could be ascribed mistakenly to the action of direct selection –e.g., could be thought of as “biases” in the occurrence of SCs and amino acids– a mistake that could then lead to additional misinterpretations regarding the evolutionary and molecular-biological mechanisms and processes shaping the primary structure of DNA. Furthermore NBDM may create sequences with very high or very low total mutation rate while sequences meander in primary-structural space, leading to bursts and lulls in both evolutionary change and polymorphism generation that would affect both SC usage and amino acid composition and could be misinterpreted as being due to directional selection, shifting constraints, etc. (since content-independent mutation and genetic drift cannot account for them). The above not withstanding, the applied-mathematical and retrospectivo-inferential challenges entailed by the reality of NBDM are very likely solvable, and solving them will, among other things, open the way to methods that can estimate accurately amino-acid replaceabilities from coding regions that diverged under NBDM.

Estimating these replaceabilities will certainly require accurate estimates of the empirical NBDM regimes that have hit consistently a given region of DNA in at least two of three aligned sequences during a period of interest. This poses serious problems. In order to estimate NBDM parameters accurately, one will have to pool very large numbers of mutations and/or substitutions generated by NBDM regimes that are similar enough to justify pooling the substitutions they generate. However, we saw above that NBDM regimes change very markedly across the genome and it is moreover clear that for many, if not for most, genomic regions the pattern of germline NBDM along chromosomes is likely to change over evolutionary time at least as rapidly as those of chromatin compaction and of legitimate and “background” transcription. This transiency may be especially pronounced in germline cells since in these cells many genes should be constitutively repressed and should therefore be embedded in chromatin whose compaction undergoes erratic change as a pleiotropic side effect of evolutionary change affecting other features (as one would expect, e.g., for the pattern of “background” transcription; see below). Therefore it will be difficult to group sequences that have shared similar regimes of NBDM mutation over a given period of interest with the goal of gathering from them enough substitutions for parameter estimation. However, and as alluded to above, like most patterns governed by mutation pressure, the pattern of motif preferences takes very long to be substantially modified by a change in mutation, and therefore commonalities in motif preferences may allow one to group aligned genomic regions of presumably similar average long-term mutational past, where attention will have to be given to sampling events evenly from many internal lineages connecting the grouped sequences. One can only hope that local NBDM regimes will not turn out to oscillate constantly through phases that have idiosyncratic substitutional spectra –rather than to depart fitfully but shortly from a modal regime– since otherwise even the best “average” NBDM regime would be a reified statistical artifact with no mechanistic generation-by-generation relevance. And of course the task will become even more daunting if, as it is likely to become necessary, one will have to estimate 1024×4 and larger NBDM matrices, rather than simple 64×4s.

Be the above as it may, when one will be able to estimate NBDM regimes accurately and determine reliably that a regime of NBDM has hit more or less consistently a genomic region of interest during a suitable period of its evolutionary history, methods for estimating amino-acid replaceabilities will be in the position of delivering accurate estimates, notwithstanding the applied-mathematical hurdles created by NBDM. Also here one must hope, however, that amino-acid replaceabilities will not turn out to differ by much across proteins, i.e., that not many proteins have adapted their structure so their function is more tolerant of the amino acid replacements that are most likely to be generated by the regime of NBDM which the encoding genes are most frequently subjected to (as proposed for 4×4 mutation by Stoltzfus [32]). Otherwise the requirements in terms of modelling complexity, retrospective data-mining sophistication, and amounts of data necessary for parameter estimation, will become even more staggering. The fact, however, that motif preferences and their reaction to changes in GC content are very conserved across vertebrates lets this possibility appear as less likely.

The above, however, does not address the fundamental question of what the ultimate factor is that varies along chromosomes and causes the regimes of NBDM to differ across GC contents as it was shown above (differences that, as noted, would be presumably much more marked if one had sorted and grouped genomic regions according to ordering variables that reflect the true heterogeneities of the regimes of NBDM in a less crude way than GC content does it). One would be tempted to postulate that GC content is the independent variable changing along chromosomes for mysterious reasons and compelling NBDM to follow suit, but the fact that in coding and non-coding DNA the preference-derived GCvsAT pressures correlate with GC content with astoundingly high R^2^s, despite these pressures being fully independent from GC content in particular and from the base composition in general, appears to point to the contrary. And in any case, the causation problem is not solved by giving a driving role to GC content, NBDM, or to both, since in that case which process would drive the driver? Several molecular processes will be discussed below that may be interacting to produce the patterns discussed so far, but we are far from believing to be providing a fully compelling scenario.

Four fundamental facts must be considered by any explanation of the primary-structural steady state of vertebrate genomes. These are i) the fact that the number of DNA replication rounds which germline nuclear DNA undergoes is strongly correlated with mutation and substitutions rates [33], [34] (review by Li *et al* [35]); ii) the fact that in the vertebrate germline several rounds of mitotic DNA replication take place between the zygote and the ovule, and even more between the zygote and sperm cells, compared to a single meiotic one; iii) the fact that despite all of this, unexpectedly many mutations are generated during meiosis from *Saccharomyces* to *Drosophila* and *Mus* [36] (later work cited in Drake [37]); and iv) the fact that qualitatively and quantitatively the pattern of mutation in regions with compacted chromatin, low spontaneous DNA damage, and little or only sporadic repair activity must be different from that in regions with decompacted chromatin and high levels of both spontaneous DNA damage and repair activity –a difference likely to be compounded by the concomitance of major repair activities with DNA replication and by the higher incidence of “background” transcription expected in regions with decompacted chromatin, which is relevant because transcription is both mutagenic and recombinogenic (see below). Therefore the mutational determinants of the primary-structure of vertebrate genomes could be found equally well among the events accompanying replication and germline mitotic DNA repair during and before the *S* phase, as among the corresponding meiotic events, or could be a mixture of both.

We will see below that regions with high levels of spontaneous DNA damage and repair activity are likely to have uncompacted chromatin, contain many genes, and replicate early, and that in these regions the non-transcribed strands of genes, and non-genic DNA in general, tend to accumulate lesions which are repaired at the time of DNA replication, triggering non-scheduled double-strand breaks (DSBs) as DNA polymerases stall and replication forks collapse upon encountering such lesions. Furthermore, because their chromatin is often decompacted and because they are rich in transcription-enhancing elements, such regions should experience a higher incidence of “background” RNA synthesis [38] (earlier citations in [39]), which during the *S* phase should lead to DSBs of the type triggered by head-on collisions of a transcription and a replication fork [40] as well as to DSBs triggered by transcribed-strand-to-transcript DNA-RNA hybridization [41], [42], [43], [44], an interaction possibly compounded by the fact that during “background” transcription the potential hybridization partners should lack both the primary-structural safeguards evolved by real genes to inhibit the hybridization (introns?) and the normal surrounding transcriptional syndrome which allows suppression proteins to gather and be active in the proximity of real genes under expression [45].

Not many molecular mechanisms appear capable of generating through NBDM the pattern of motif over-/under-representations documented above, motif preferences that, notably, correlate most strongly with trinucleotide occurrences when GC content is intermediate (which, incidentally, and possibly not coincidentally, is the GC shown by most mammalian genes; Figure 9). There is, e.g., no reason known to us for one to expect that major processive DNA polymerases change the neighbor-base-dependence of their, in any case very low, mutagenicities when they replicate regions of different GC (but different replication factories could be used for early and late replication [20]. The only candidates that come to our mind are lesion-bypass and extension-after-bypass polymerases (LBPs) which are indeed used frequently in gene-rich regions during the mitotic and meiotic stages of the germline. Furthermore, i) the tertiary structure of LBPs [46] appears to be malleable enough for the mutagenicities of the various LBPs to show a diversity of reactions to the bases at the site preceding and that following their target sites (i.e., heterogeneous NBDM tendencies), and ii) the functional diversity and sheer number of LBPs make it plausible that their eventual differential availability and utilization both along chromosomes and at various times during genome expression and DNA replication [47], together with the possibly disparate reactions of their activities to temporal and spatial intranuclear heterogeneities [48] (citations in [20]), may generate heterogeneities in the average neighbor-base-dependence of mutation along chromosomes when genes are expressed and repaired and when chromosomes are replicated and repaired, all of which could happen without compromising the central lesion-bypass and/or extension-after-bypass functions.

Lesion-bypass polymerases are known to be involved in DNA repair. Many lesions in the transcribed strand of genes are repaired immediately through transcription-coupled repair (TCR) [49], [50] but when the lesions affect the non-transcribed strand and non-transcribed DNA in general, they are repaired at the time of DNA replication [51], [52], [53]. Heddle and Bielas [54] have indeed proposed that much of DNA repair requires that damage be flagged by a polymerase stalled in front of the damage (e.g., a polymerase that was generating legitimate or illegitimate transcripts, or was replicating the genome); which is consistent with the existence of special repair mechanisms for the non-transcribed strand of active genes in terminally differentiated and thus non-dividing neurons [55]. This is important because a stalled DNA polymerase can trigger the intervention of LBPs during *S* phase repair with or without a non-scheduled DSB and with or without the involvement of recombinational repair (Fig. 3 in Symington [56]). During the mitotic and meiotic *S* phases, DSBs are often generated when replication forks collapse upon encountering a nick or a tract of ssDNA [57], and are repaired mainly through recombinational repair with LBP participation [58], [59], [60], [61], [62], [63]. Stalled RNA polymerases, on the other hand, are also very common events in active mammalian cells and trigger TCR [49], [64] which requires that a few dozen bases be hydrolyzed to expose the blocking lesion [65], creating a gap that later is filled by a high-accuracy DNA polymerase but which could be filled by an LBP accidentally. Similarly, the advancing replication fork often leaves behind stretches of ssDNA centered around DNA damage that are later filled through a combination of LBP-mediated and normal high-accuracy DNA synthesis [57], [66], [67] where, as was noted above, the specific LBPs deployed could vary depending on availability, cell-cycle stage, etc.

Above we wrote that several types of DNA lesions tend to occur more frequently in gene-rich DNA regions whose chromatin is decompacted [68] and in which many genes are being expressed (but which being wide can contain punctual presences of inactive or very highly active genes [69], [70]. These regions replicate early [71], [72], have higher GC than gene-poor regions [73], [74], [75], and tend to experience most rearrangements and non-scheduled DSBs [76], [77]. Interestingly, the number of mitotic S-phase DSBs is about fifty per *S* phase per mammalian genome [78], which over the several mitotic divisions of the germline gives a number fully in the order of magnitude of the number of DSBs introduced by the *Spo*11 protein allegedly to facilitate chromosomal segregation (reviewed in [79]). This suggests strongly that sister-chromatid recombination and LBP usage during mitotic growth may have major mutagenic consequences in mammalian cells in general [80] and in the mammalian germline in particular, with or without involvement of classic DSBs [81], although the targeted apoptosis of mutated germline cells should lower these numbers somewhat [82], Van Valen, pers.com; Drake pers.com. These mitotic events do not have to occur at the same locations in which both endogenous *S*-phase DSBs [83] and *Spo*11-generated DSBs tend to occur during meiosis, which may explain the weakness of the correlations documented so far between GC content and recombination intensities measured transmission-genetically [84] (recent citations in [85]), together of course with the often overlooked facts that an insignificant number of *Spo*11-generated DSBs are resolved via crossing over rather than via gene conversion (e.g., one in ten in humans) and that the factors steering the resolution either way are known to be downstream of those determining where *Spo*11 cuts the DNA [86]. Indeed the total mutagenic input may be contributed not only by meiotic crossing over but also by meiotic conversion, mitotic mutation, mitotic recombination and conversion, and by meiotic *S* phase recombination repair. Finally, processive DNA polymerases do not extend well from some mismatches and have to pause until the complex's exonuclease is mobilized (citations in Jiricny [87]), which can lead to complex dissociation and could allow partial accidental extension by LBPs that would intervene at different rates depending on a variety of factors, e.g., dNTP levels [57], levels that can vary between early and late replication [89, and citations therein].

In the above narrative, the main distinction lies between gene-rich and gene-poor regions which we argued should have a tendency to evolve and maintain different primary structures because on average they tend to have different syndromes of DNA metabolism. But the various molecular processes involved in generating these average between-group primary-structural differences could also account for the primary-structural differences observed within gene-rich regions and within gene-poor regions. Indeed it is highly unlikely that, e.g., the patterns of chromatin compaction, the timing and location of scheduled and non-scheduled transcription and of DNA replication and repair, and the landscape of DSB incidence, are rigidly homogenous within gene-rich regions and within gene-poor regions. Such within-region heterogeneity could very well be involved in determining the smaller-scale heterogeneities shown by NBDM regimes within gene-rich and within gene-poor regions. For instance DSBs hot spots should be found there where forks of nonscheduled transcription initiated regularly during the mitotic and/or meiotic S phase at a given distance from a replication origin, tend to collide head-to-head with a replication fork initiated at a nearby replication origin [40]. These collisions would generate DSBs in a narrowly delimited area and therefore lead to a high incidence of recombinational DNA repair, LBP activity, etc, which would affect local primary structure despite the absence of any local features that “attract” DSBs or recombinational repair. And similar scenarios but with inverted polarities would result in lower incidence of such head-on collisions, DSB generation, etc. Note, as a curiosity, that whenever background transcription bubbles will be found to be involved in determining such spots of very low or very high DSB incidence, it can be expected that the spots will be as unstable evolutionarily as the pattern of background transcription.

We favor the above scenario in which DSB incidence is coupled to LBP activity and LBP diversity in tracking the background heterogeneities ensuing from the spatio-temporally changing landscapes of replication and of scheduled and non-scheduled transcription. Much in this scenario is indeed evolvable to some extent both neutrally and through natural selection. However, we already mentioned above that different factories could be used for early and late DNA replication [20]. Furthermore, John Drake and Gerald Holmquist (pers.comm.) have proposed to us additional mechanisms that can deliver NBDM and may react to the same background heterogeneities, and that therefore could also contribute to the NBDM-generated primary-structural gradients studied in this paper. Drake pointed out that both processive DNA polymerases (and their proofreading functions) as well as DNA mismatch repair (MMR) are sensitive to the immediate and even the non-so-immediate nucleotide context. Holmquist mentioned unpublished *in vitro* experiments of his demonstrating that the stacking energies of the two non-disjunct dinucleotides in a trinucleotide (e.g., AT and TG in ATG) determine very strongly the probability that a damaged middle nucleotide be repaired by MMR. Drake mentioned that polymerases touch DNA at roughly 20–50 places scattered upstream and downstream from the insertion site, where the contacts change their relative position considerably as a polymerase negotiates new primary structure; and also that MMR efficiency varies between lagging and leading strands and with increasing distance from the replication origin [89], [90]. These effects deliver or could deliver NBDM but it remains to be seen to which extent they –and the background heterogeneities to which they may react– can both be conserved and diverge evolutionarily and so account for the general conservation, the short-term oscillations, and the few lineage-typical differentiations of motif preferences that we saw above (e.g., between vertebrates and *Ciona*).

One could argue that the detailed molecular-biological foundations of the phylogenetically highly conserved primary-structural patterns presented above are not that important since the patterns' evolutionary generation and population-genetic preservation require only that the neighbor-base dependence of mutation be evolvable through natural selection, which in turn only requires that enough genetic variance with large enough additive phenotypic impact be available initially and that mutations impairing the adaptation be selectable-against afterwards, two requirements that are supervenient to the specifics of the molecular interactions underlying the genetic and phenotypic variation of interest. It is becoming clear, however, that DNA repair intensity and DNA repair mechanisms in general, and the containment of genomic instability ensuing from gene expression and DNA replication and from the interaction between these two processes in particular, are not only under strong selection but may also be –possibly because costly metabolically [55] and detrimental to cell-division speed [91]– among the ultimate bioengineering quantities responsible for the evolutionary trade-offs known to exist between major life-history traits. Evidence already exists that mouse soma cells are less efficient at global DNA repair than human ones [92], which is in accordance with mice being fast-reproducing, shorter-lived *r*-strategists; and it is also known that cells with DSBs in fast-growing *Drosophila* embryos are removed from the embryo so they do not slow down development [93, J.Spofford pers.comm.]. Therefore, a detailed understanding of the ultimate molecular foundations of DNA repair mechanisms and of their phylogenetic diversification is not only of obvious biomedical importance but also likely to provide crucial indications about the ultimate boundaries imposed on the diversity of life histories on earth. However, and unfortunately for molecular evolutionists, it is already clear that somatic cells can differ drastically from each other in their repair abilities [55] and that so can stages of the germline [94], so that major adaptive changes in somatic-repair allocation could very well have been evolved without concomitant changes in germline mutability, i.e., without leaving traces in the pattern of evolutionary divergence. If striking, such differences between soma and germline should, among other things, make attempts at evaluating somatic-mutation advantages on the basis of germline mutation rates estimated from inferred evolutionary substitutions into problematic, if not directly hopeless, undertakings. Fortunately, however, the genes that by mutating can modulate global DNA repair are not likely to be many so that major changes in somatic and germline repair may have often been triggered by single-gene mutations (possibly with ulterior oligogenic fine-tuning), making the neontological study of the evolutionary differentiation of somatic repair in general, and within vertebrates in particular, into a possibly very rewarding endeavor. Furthermore, high-throughput single-molecule sequencing techniques are becoming powerful enough to characterize directly and at the whole-genome level the regimes of somatic mutation in different tissues and individuals, so also here is the neontological approach about to provide exciting answers.
