# Correction: Highly Conserved Regimes of Neighbor-Base-Dependent Mutation Generated the Background Primary-Structural Heterogeneities along Vertebrate Chromosomes

**DOI:** 10.1371/annotation/bc789a4f-9fb7-41df-bc6f-d26203d09dbe

**Published:** 2013-09-19

**Authors:** Marcos A. Antezana, I. King Jordan

There was an error in the legend for Figure 23, "Slopes of the trinucleotide preference-occurrence correlations in non-genic and intronic DNA vs. GC content." 

The correct figure legend is: 

The slopes of the occurrence-preference correlation between trinucleotide-motif preferences and occurrences in groups of nongenic and intronic DNA of increasing GC (left, right; R^2^s are shown in the figure 22 and Figure 3). 

